# Interaction between DLC-1 and SAO-1 facilitates CED-4 translocation during apoptosis in the *Caenorhabditis elegans* germline

**DOI:** 10.1038/s41420-022-01233-9

**Published:** 2022-11-03

**Authors:** Dandan Zhang, Haibin Yang, Ling Jiang, Chan Zhao, Mengjun Wang, Boyi Hu, Cong Yu, Zhiyi Wei, Yu Chung Tse

**Affiliations:** 1 School of Life Science and Technology, Harbin Institute of Technology, Harbin, 150001 China; 2 School of Life Sciences, Department of Biology, Southern University of Science and Technology, Shenzhen, 518055 China; 3 Guangdong Provincial Key Laboratory of Cell Microenvironment and Disease Research, Southern University of Science and Technology, Shenzhen, 518055 China; 4 School of Biological Sciences, Faculty of Science, The University of Hong Kong, Hong Kong, China; 5 Department of Biology, State Key Laboratory of Environmental and Biological Analysis, Hong Kong Baptist University, Hong Kong, China; 6 Core Research Facilities, Southern University of Science and Technology, Shenzhen, 518055 China

**Keywords:** Apoptosis, Developmental biology

## Abstract

Apoptosis is one of the major forms of programmed cell death, and it serves vital biological functions in multicellular animal and plant cells. The core mechanism of apoptosis is highly conserved in metazoans, where the translocation of CED-4/Apaf-1 from mitochondria to the nuclear membrane is required to initiate and execute apoptosis. However, the underlying molecular mechanisms of this translocation are poorly understood. In this study, we showed that SAO-1 binds DLC-1 and prevents its degradation to promote apoptosis in *C. elegans* germ cells. We demonstrated that SAO-1 and DLC-1 regulate CED-4/Apaf-1 nuclear membrane accumulation during apoptosis. Isothermal titration calorimetry-based assay and high-resolution crystal structure analysis further revealed that SAO-1 interacted with DLC-1 to form a 2:4 complex: each of the two β-sheets in the SAO-1 peptide interacted with two DLC-1 dimers. Point mutations at the SAO-1-DLC-1 binding interface significantly inhibited apoptotic corpse formation and CED-4 nuclear membrane accumulation within *C. elegans* germ cells. In conclusion, our study provides a new perspective on the regulation of CED-4-mediated apoptosis.

## Introduction

Apoptosis is a biological intracellular programmed cell death that eliminates cells that are under developmental or exposed to environmental stimuli within multicellular organisms [1, 2]. The genetics and molecular mechanisms have been extensively studied for decades using model organisms such as *Caenorhabditis elegans* (*C. elegans*), *Drosophila melanogaster* (drosophila), and *Danio rerio* (zebrafish). In *C. elegans*, apoptosis occurs during two developmental stages and in two different types of tissues. During embryonic and postembryonic development of the soma, 131 cells are eliminated by programmed cell death [3]; 113 of these cells die during embryonic development, and 18 die during postembryonic development [4, 5]. In contrast, approximately one-half of the germ cells are eliminated from the germline of adult hermaphrodites [6]. This process is thought to maintain the size of the germline by maintaining a balance between the number of new cells produced via mitosis at the distal end of the gonad and the quality of the oocytes by removing damaged or unwanted germ cells. Alternatively, viral infection, DNA damage, and environmental stress can induce apoptosis [7–11].

The core mechanism of apoptosis is highly conserved in nematode and mammalian cells. In living cells, before apoptosis is triggered, CED-9 (*C. elegans*)/BCL-2 (human) is located on the outer mitochondrial membrane and binds to CED-4 (*C. elegans*)/Apaf-1 (human) to inhibit CED-4/Apaf-1 activity. In addition, CED-3 (*C. elegans*)/Caspase (human) is tethered to the nuclear membrane. When apoptosis is activated, the upstream BH3 protein EGL-1 binds to CED-9/BCL-2. The resultant conformational change in CED-9/BCL-2 leads to the release of CED-4/Apaf-1 from mitochondria and its subsequent translocation to the nuclear membrane [12]. Finally, the CED-3/Caspase protein is activated, and cell death ensues [13–19]. An increasing number of studies have suggested that the translocation of CED-4/Apaf-1 from mitochondria to the nuclear membrane is required to initiate and execute apoptosis [20–22]. However, the protein and molecular mechanisms involved in CED-4/Apaf-1 translocation remain unclear.

Some hints into how CED-4/Apaf-1 is translocated to the nuclear membrane can be found in a study on *C. elegans* dynein light chain 1 (DLC-1). *C. elegans* DLC-1 is highly conserved with homologous DYNLL2 (dynein light chain LC8-type 2) in mammalian cells. Despite a previous theory suggesting that DLC-1 functions as a cargo adapter, DLC-1 has been reported to participate in apoptosis within human, drosophila and *C. elegans* cells [22–24]. Recent research showed that DLC-1 regulated CED-4 nuclear accumulation during apoptosis in *C. elegans* germ cells [22].

The GYF (glycine-tyrosine-phenylalanine) domain is highly conserved (W-X-Y-X6-11-GPF-X4-M-X2-W-X3-GYF) in metazoans. It binds to proteins with a conserved proline-rich sequence [25–28]. Studies have reported that GYF domain proteins play critical roles in a variety of intracellular processes and regulatory downstream signaling [29]. In *C. elegans*, the GYF domain-containing protein SAO-1 is a suppressor of APH-1, a component of γ-secretase, that may interact with the E3 ubiquitin ligase subunit SEL-10 to antagonize Notch signaling [30]. However, the cellular and molecular functions of SAO-1 remain largely unclear.

Here, we report on the reevaluation of the function of SAO-1 in *C. elegans* development. We found that SAO-1 is specifically expressed in primordial germ cells (Z2 and Z3) during the early developmental stages and is highly expressed in the germline throughout the entire *C. elegans* life cycle. Although SAO-1 is expressed in germ cells, *sao-1* depletion does not influence germline development. Moreover, we observed a reduced number of apoptotic corpses, the remains of cells after undergoing programmed cell death, within the germlines of both *sao-1* mutant and double *sao-1* and *ced-9(n1653)* mutant worms with accelerated apoptosis. Performing mass spectrometry analysis, we identified DLC-1 as an interacting partner of SAO-1. Indeed, depletion of *dlc-1* led to the acquisition of phenotypes observed in *sao-1*-mutant worms, and SAO-1 inhibited the degradation of DLC-1. Furthermore, we demonstrated that SAO-1 and DLC-1 regulated CED-4 nuclear membrane localization in germ cells. These results indicate that SAO-1 and DLC-1 play critical roles in the core apoptotic pathway. Biochemical and mutational analyses further revealed that SAO-1 directly interacted with DLC-1. Surprisingly, the N-terminal GYF domain in SAO-1 did not play a role in this interaction. DLC-1 bound the C-terminal β-sheet structures of SAO-1. Notably, strains with point mutations in this region showed a reduced apoptosis rate. A Crystallography analysis further predicted that each of these β-sheets binds to one DLC-1. Overall, our results suggest that SAO-1 binds to DLC-1 and then promotes the translocation of CED-4 from the mitochondrial outer nuclear membrane to the nuclear membrane in the *C. elegans* germline during apoptosis.

## Results

### SAO-1 is specifically expressed in *C. elegans* primordial germ cells and germline

**Fig. 1:**
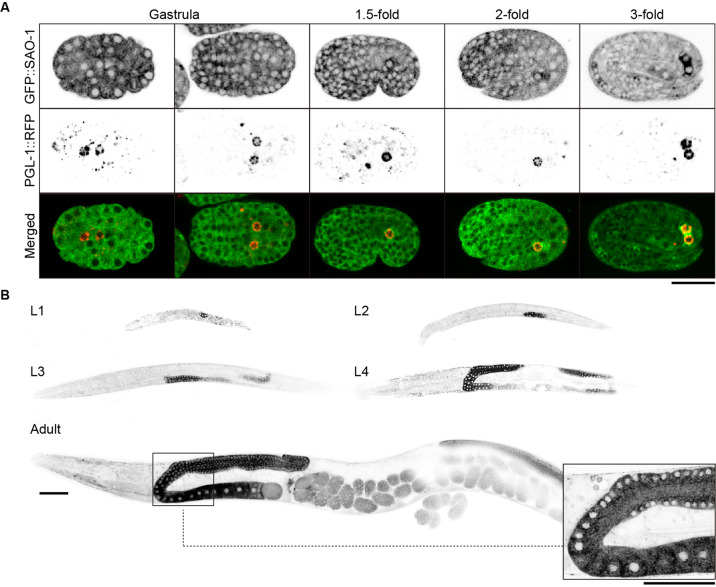
The expression of SAO-1 in *C. elegans* primordial germ cells and germline. **A** Representative confocal images of the expression of GFP::SAO-1 in the embryos at the gastrula stage, 1.5-fold stage, 2-fold stage and 3-fold stage. Scale bar, 20 μm. **B** Representative confocal images of the expression of GFP::SAO-1 in worms at L1, L2, L3, L4 and adult stage. Scale bar, 50 μm.

To investigate the expression pattern of SAO-1 during embryonic development, we generated a transgenic *C. elegans* strain expressing GFP::SAO-1 or SAO::RFP. The CRISPR/Cas9 genome-editing technique [31] was employed to insert the GFP-coding DNA sequence into the 5ʹ-end or RFP-coding DNA sequence at the 3’-end of endogenous *sao-1* on chromosome V. During early development, GFP::SAO-1 was expressed in all of the embryonic cells and diffused throughout the cytoplasm (Fig. 1A). During development, GFP::SAO-1 gradually accumulated in PGL-1::RFP-labeled primordial germ cells from the 1.5-fold stage to the L1 stage (Fig. 1A). PGL-1 is a predicted RNA-binding protein that is associated with P granules in germ cells at all stages of development [32] Notably, GFP::SAO-1 was specifically expressed in germ cell cytoplasm from the L2 to the adult stage (Fig. 1B). Moreover, SAO-1::RFP exhibited an expression pattern identical to that of GFP::SAO-1 (Fig. S1).

### SAO-1 facilitates apoptosis in the *C. elegans* germline

Because SAO-1 is expressed explicitly in the germline, we wondered whether SAO-1 is required for germline development and oocyte production. Thus, we depleted SAO-1 by RNA interference (RNAi) to examine the germline morphology and analyze the embryonic viability. Differential interference contrast microscopy imaging revealed no significant differences in germline morphology between control (*L4440 (RNAi)*) and *sao-1(RNAi)*-treated worms (Fig. S2A, B). In addition, we generated a *sao-1*-deletion mutant (*sao-1(ΔFL*)) via CRISPR/Cas9 and replaced the entire endogenous *sao-1* with a blue fluorescent protein (BFP) gene. Thus, BFP was a fluorescent marker for the *sao-1*-deletion mutant worms. Consistent with the RNAi depletion analysis, the morphology of germline and embryonic viability of *sao-1(ΔFL*)-mutant worms were indistinguishable from that of the control and *sao-1(RNAi)*-treated worms (Fig. S2A, B). These results indicate that SAO-1 is dispensable for germline development and progeny viability.

**Fig. 2:**
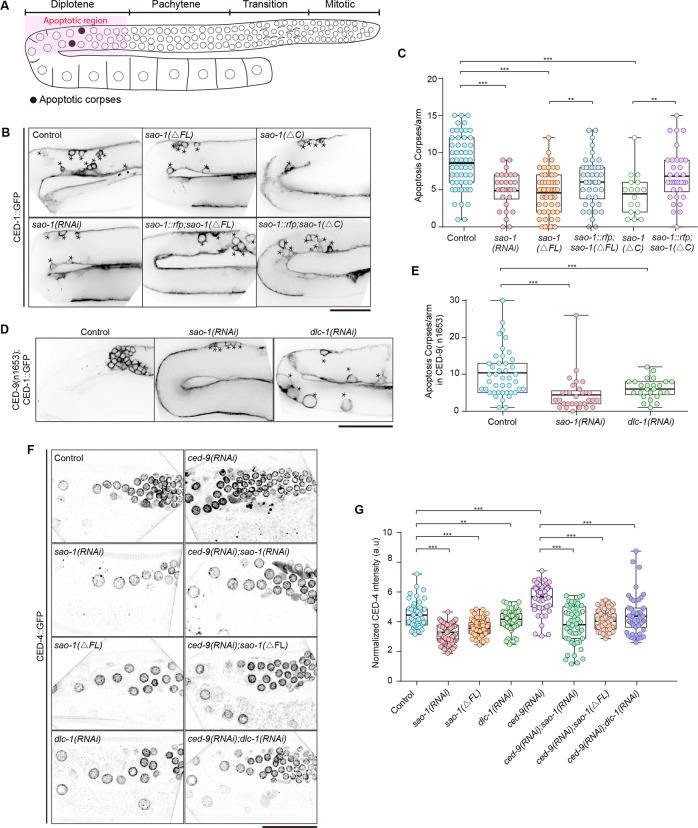
SAO-1 and DLC-1 promote *C. elegans* germ cell apoptosis. **A** Schematic representation of the adult *C. elegans* hermaphrodite germline. **B** Representative confocal images of the apoptotic corpses labeled by CED-1::GFP (black asterisk) in control, *sao-1 (RNAi)*, *sao-1(ΔFL)*, *sao-1(ΔC), Ex[sao-1::rfp]*;*sao-1(ΔFL)* and E*x[sao-1::rfp]*;*sao-1(ΔC)*. Worms were imaged at 48 h after L4 molt. Asterisks: apoptotic corpses. The schematic representation of *sao-1(ΔC)* please refer to Fig. 4C. **C** Quantification of the number of apoptotic corpses in **B**, each dot in the chart represents a single worm. **D** Representative confocal images of the apoptotic corpses in *ced-9(n1653)* in control, *sao-1(RNAi)*, and *dlc-1(RNAi)*. **E** Quantification of the number of apoptotic corpses in **D**, each dot in the chart represents a single worm. **F** Representative confocal images of CED-4::GFP in control, *sao-1(RNAi), sao-1(ΔFL), dlc-1(RNAi)*, *ced-9(RNAi)*, *sao-1(RNAi);ced-9(RNAi)*, *sao-1(ΔFL);ced-9 (RNAi)*, and *dlc-1(RNAi);ced-9(RNAi)*. **G** Quantification of CED-4::GFP intensity in **F**, each dot in the chart represents a single nuclear. Data were analyzed by two-tailed Student’s *t* test; Error bars, ±SEM; **p < 0.01, ***p < 0.001. a.u., arbitrary unit. Scale bar, 50 μm.

Apoptosis is another well-known cellular process that occurs in the germline. Therefore, we examined whether SAO-1 plays a role in the apoptosis pathway of germ cells. We imaged the apoptotic region of the germline expressing CED-1::GFP, an early apoptotic marker, in control, *sao-1(RNAi)*-treated and *sao-1(ΔFL*)-mutant worms, and counted the number of apoptotic corpses 48 h after L4 molt (Fig. 2A). In *sao-1(RNAi)-*treated and *sao-1(ΔFL*)-mutant worms, the number of apoptotic corpses in the apoptotic region was significantly reduced (Fig. 2B, C). In addition, we expressed SAO-1::RFP in *sao-1(ΔFL*)-mutant worms using the extrachromosomal array method, and observed that the number of apoptotic corpses was partially restored (Fig. 2B, C). These results suggest that SAO-1 regulates germ cell apoptosis.

Because the absence of SAO-1 inhibits apoptosis of the germ cells, we speculated that SAO-1 may regulate the core apoptosis pathway. If SAO-1 promotes apoptosis through the core pathway, then its depletion should inhibit the accelerated apoptosis rate in *ced-9(n1653)*-mutant worms. To test this hypothesis, we depleted SAO-1 via RNAi in *ced-9(n1653)*-mutant worms and found that the number of apoptotic corpses was significantly reduced compared to that in the control (Fig. 2D, E). This result indicates that SAO-1 facilitates apoptosis through the core apoptotic pathway in the *C. elegans* germline.

### SAO-1 depletion inhibits CED-9-mediated nuclear membrane accumulation of CED-4

Next, we investigated whether SAO-1 regulates core apoptotic executioner proteins, such as CED-9 and CED-4. We began by imaging the apoptotic region of the germline expressing RFP::CED-9. We did not observe significant intensity changes in control and *sao-1(RNAi)*-treated worms (Fig. S3A, B). In turn, depletion of CED-9 did not affect the GFP::SAO-1 intensity (Fig. S3C, D). However, CED-4::GFP intensity was markedly reduced in *sao-1(RNAi)*-treated, and the *sao-1(ΔFL)-*mutant (Fig. 2F, G) compared to control nuclear membrane. Furthermore, it has been reported that loss of CED-9 leads to an increased accumulation of CED-4 in the nuclear membrane with concomitant apoptosis acceleration [12]. Thus, we speculated that loss of SAO-1 might inhibit the accumulation of CED-4 on the nuclear membrane after CED-9 depletion. We imaged and analyzed the intensity of CED-4::GFP in *ced-9(RNAi), ced-9(RNAi);sao-1(RNAi)* and *ced-9(RNAi);sao-1(ΔFL)* worms. As expected, nuclear membrane-localized-CED-4::GFP increased in the *ced-9(RNAi)* germ cells (Fig. 2F, G). However, the intensity of CED-4::GFP was significantly reduced in the *ced-9(RNAi);sao-1(RNAi)* and *ced-9(RNAi);sao-1(ΔFL)* germ cells (Fig. 2F, G), suggesting that SAO-1 facilitates the nuclear membrane accumulation of CED-4.

### SAO-1 stabilizes the cytoplasmic pool of DLC-1

**Fig. 3:**
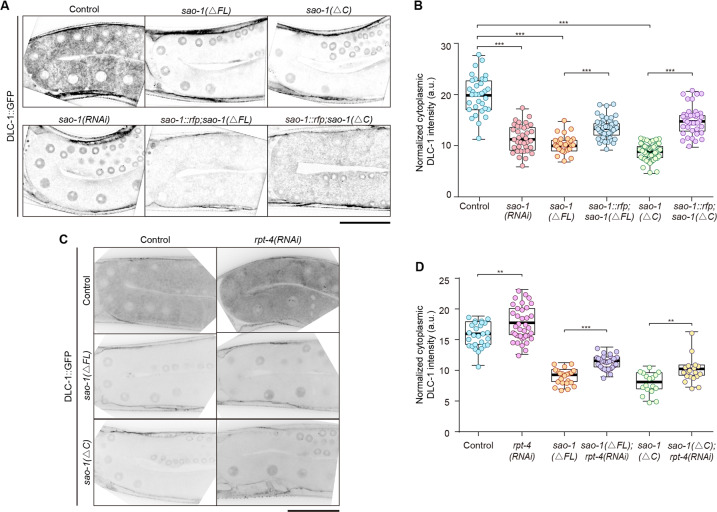
SAO-1 regulates DLC-1 in the cytoplasm of the *C. elegans* germline. **A** Representative confocal images of DLC-1::GFP in control, *sao-1(RNAi)*, *sao-1(*Δ*FL)*, *sao-1(ΔC), Ex[sao-1::rfp]*;*sao-1(ΔFL)* and *Ex[sao-1::rfp]*;*sao-1(ΔC)*. The schematic representation of *sao-1(ΔC)* please refer to Fig. 4C. **B** Quantification of normalized DLC-1::GFP intensity in **A**. **C** Representative confocal images of DLC-1::GFP in control, *rpt-4 (RNAi), sao-1(ΔFL)*, *sao-1(ΔFL);rpt-4(RNAi)*, *sao-1(ΔC)* and *sao-1(ΔC);rpt-4(RNAi)*. **D** Quantification of cytoplasmic DLC-1::GFP intensity in **C**. Each dot in the chart represents a single worm. Data were analyzed by two-tailed Student’s *t* test; Error bars, ±SEM; **p < 0.01, ***p < 0.001. a.u. arbitrary unit. Scale bar, 50 μm.

Considering that our results showed that SAO-1 affects apoptosis and contains a GYF domain known to interact with proteins carrying minimal consensus proline-rich sequences [25–28], we sought to determine SAO-1 binding targets. A mass spectrometry analysis led to the identification of 734 proteins (Table S1). Interestingly, one of these candidates, dynein light chain 1 (DLC-1), has been reported to promote CED-4 accumulation at the nuclear membrane [22] (similar to our results Fig. 2F, G), and to decrease the number of apoptotic corpses in *ced-9(n1653)*-mutant worms (Fig. 2D, E). Therefore, we speculated that SAO-1 and DLC-1 function via the same molecular pathway to regulate CED-4 activity. Thus, we determined the subcellular localization of DLC-1::GFP in control, *sao-1(RNAi)-*treated, and *sao-1(ΔFL)*-mutant germ cells. In control worms, DLC-1::GFP was diffused throughout the germ cell cytoplasm (Fig. 3A). In *sao-1(RNAi)-*treated and *sao-1(ΔFL)*-mutant worms, the intensity of DLC-1::GFP in the germ cell cytoplasm was markedly diminished (Fig. 3A, B), but no differences were observed in germ cell nuclei (Fig. S4A, B) or muscle cells (Fig. S4A, C). Furthermore, the expression of SAO-1::RFP in *sao-1(ΔFL*)-mutant worms partially rescued DLC-1::GFP intensity in germ cell cytoplasm, as determined using an extrachromosomal array (Fig. 3A, B). These results indicate that SAO-1 may stabilize the cytoplasmic pool of DLC-1 in the germline. One possible explanation for these results is that SAO-1 may inhibit the degradation of DLC-1. To test this possibility, we depleted RPT-4, a proteasome subunit, to decrease proteasome-mediated degradation and measured DLC-1::GFP intensity in control, *rpt-4(RNAi)*, *sao-1(ΔFL)* and *rpt-4(RNAi);sao-1(ΔFL)* worms. The germ cell cytoplasm of the worms treated with *rpt-4(RNAi)* had significantly greater DLC-1::GFP intensity than the control worms (Fig. 3C, D), as predicted. Interestingly, the DLC-1::GFP intensity in *rpt-4(RNAi);sao-1(ΔFL)* worms was also greater than that in *sao-1(ΔFL)*-mutant worms (Fig. 3C, D). These results indicate that SAO-1 can protect DLC-1 from proteasome-mediated degradation.

### SAO-1 directly interacts with DLC-1

**Fig. 4:**
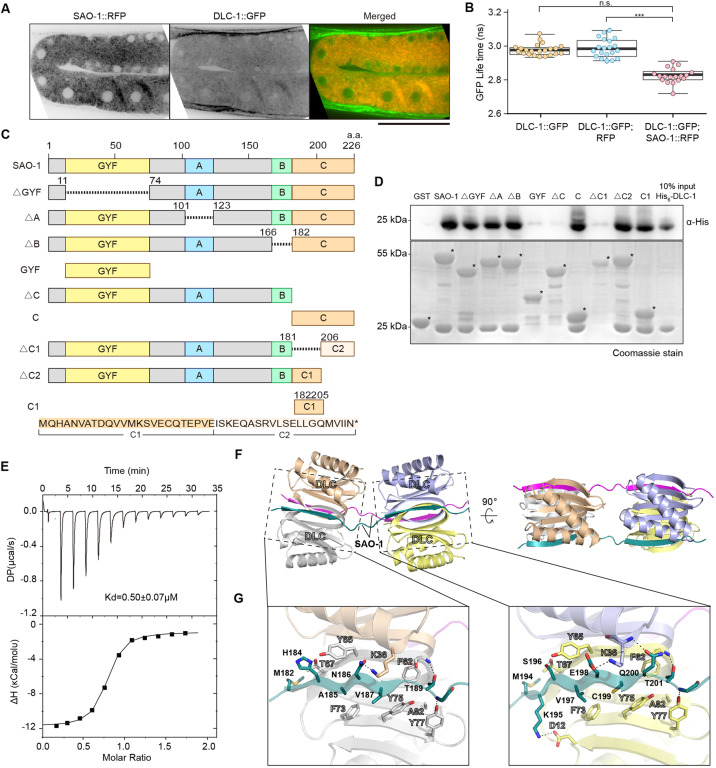
SAO-1 interacts with DLC-1. **A** Representative confocal images of DLC-1::GFP and SAO-1::RFP. Scale bar, 50 μm. **B** Quantification of DLC-1::GFP fluorescent lifetime of in worms expressing DLC-1::GFP, DLC-1::GFP;RFP, and DLC-1::GFP;SAO-1::RFP. Each dot in the chart represents a single worm. Data were analyzed by unpaired Student’s *t* test; Error bars, ±SEM; n.s. not significant. ***p < 0.001. **C** Schematic of SAO-1 mutants: full-length SAO-1, *Δ*GYF(*Δ*12–73), *Δ*A(*Δ*102–122), *Δ*B(*Δ*167–181), GYF(1–73), *Δ*C(*Δ*182–226), C(182–226), *Δ*C1(*Δ*182–205), *Δ*C2(*Δ*206–226), C1 (182–205). **D** Western blot analysis of DLC-1 binding to purified GST-tag fusion proteins (GST, GST-SAO-1, GST-*Δ*GYF(*Δ*12–73), GST-*Δ*A(*Δ*102–122), GST-*Δ*B(*Δ*167–181), GST-GYF(1–73), GST-*Δ*C(*Δ*182–226), GST-C(182–226), GST-*Δ*C1(*Δ*182–205), GST-*Δ*C2(*Δ*206–226), GST-C1 (182–205)) using His antibody (top). GST-tag fusion proteins were visualized by Coomassie blue staining (bottom). Asterisks denote GST-tag fusion proteins. **E** Representative ITC curve showing the dissociation constant (Kd) and enthalpy change (*Δ*H) of SAO-1-DLC-1 protein-protein interaction. **F** Overall structure of the DLC-1-SAO-1C1(182-205) complex presented in two views. The complex contains four DLC-1 molecules that form two dimers and two SAO-1C1 peptides. **G** The molecular details of two binding interfaces. Hydrogen bonds and salt bridges were indicated by dashed lines.

Since mass spectrometry analysis revealed DLC-1 as a candidate binding partner of SAO-1, we tested its ability to interact SAO-1 at the cellular and biochemical levels. For an in vivo analysis, we co-expressed SAO-1::RFP and DLC-1::GFP and imaged germlines via spinning disk confocal microscopy. We found that both fluorescent fusion proteins were diffused within the germ cell cytoplasm and appeared to be colocalized (Fig. 4A). A Pearson’s correlation coefficient of SAO-1::RFP and DLC-1::GFP was 0.7109 (±0.01239), indicating that they were partially colocalized in the cytoplasm.

To further test the apparent colocalization of SAO-1 and DLC-1, we employed fluorescence lifetime image microscopy. Three transgenic fluorescent worm lines were generated for this analysis: DLC-1::GFP, DLC-1::GFP;RFP, and DLC-1::GFP;SAO-1::RFP. The mean fluorescent lifetime of GFP in the worms expressing either DLC-1::GFP (τ is 2.976 ± 0.005457 ns) or DLC-1::GFP;RFP (τ is 2.983 ± 0.009466 ns) was not significantly different (Fig. 4B). Importantly, the GFP mean lifetime in worms co-expressing DLC-1::GFP;SAO-1::RFP was significantly shorter (τ = 2.851 ± 0.007849 ns) compared with that in worms expressing DLC-1::GFP and DLC-1::GFP;RFP (Fig. 4B). Thus, the FRET interactions between SAO-1 and DLC-1 specifically corroborated the colocalization data and supported a direct interaction between SAO-1 and DLC-1 at the cellular level.

We also investigated the physical interaction between DLC-1 and SAO-1 in vitro. We expressed and purified GST-SAO-1 and Trx-His_6_-DLC-1 for use in pull-down assays. We found that GST-SAO-1 interacted with Trx-His6-DLC-1 (Figs. 4D and S5A), further strengthening our hypothesis of a direct interaction between SAO-1 and DLC-1.

### The SAO-1 C-terminus interacts with DLC-1 and facilitates apoptosis

Since the cellular and biochemical data demonstrated that DLC-1 directly interacted with SAO-1, we next examined the DLC-1-binding interface with SAO-1. Six different truncated forms of SAO-1 were constructed for pull-down experiments: (1) *Δ*GYF, which was missing the GYF domain (residues 12–73); (2) *Δ*A, which was missing the low-complexity domain A (residues 102–122); (3) *Δ*B, which was missing the low-complexity domain B (residues 167–181); (4) GYF, which consisted of only the GYF domain (residues 1–73); (5) *Δ*C, which was missing the C-terminal region (residues 182–226); and (6) C, which was the SAO-1 C-terminal fragment (Fig. 4C). Surprisingly, among these six truncated SAO-1 proteins, only GYF and *Δ*C could not bound DLC-1 (Figs. 4D and S5A). In contrast, all the truncated SAO-1 proteins with C-terminal fragment interacted with DLC-1 (Figs. 4D and S5A). These results demonstrated that the C-terminal region of SAO-1 but not the GYF domain is essential for DLC-1 interaction.

Considering these results, we next examined the physiological role of the SAO-1 C-terminal region in apoptosis. A transgenic mutant expressing SAO-1 without the C-terminal region (residues 182-226), *sao-1(ΔC*), was generated via CRISPR/Cas9. The number of apoptotic corpses in the *sao-1(ΔC*) worms was comparable to that in the *sao-1(RNAi)*-treated and *sao-1(ΔFL)*-mutant worms but significantly reduced compared with that in the control worms (Fig. 2B, C). On the other hand, overexpression of SAO-1::RFP in *sao-1(ΔC*)-mutant worms partially rescued the number of apoptotic cells (Fig. 2B, C). In addition, the intensity of cytoplasmic DLC-1::GFP in the *sao-1(ΔC*) worms was reduced, similar to that in the *sao-1(RNAi)* and *sao-1(ΔFL)* mutant worms (Fig. 3A, B). The reduction in signal intensity was partially restored by the expression of the extrachromosomal array SAO-1::RFP (Fig. 3A, B) and by *rpt-4(RNAi)* (Fig. 3C, D). These findings indicate that the SAO-1 C-terminal region is required for normal apoptosis in *C. elegans* germ cells.

To further dissect the putative DLC-1-binding region in SAO-1, we divided the C-terminal region into two parts and constructed two new truncated forms of SAO-1: (1) *Δ*C1, which was missing residues from 182 to 205 in the C-terminus, and (2) *Δ*C2, which was missing residues from 206 to 226 in the C-terminus (Fig. 4C). Remarkably, *Δ*C2 pulled down a significant amount of DLC-1, while *Δ*C1 lost its ability to bind DLC-1, as observed with SAO-1(*Δ*C) (Figs. 4D and S5A). Moreover, protein fragments of C1 bound and pulled down DLC-1 (Figs. 4D and S5A). Altogether, these results demonstrate that the C1 half of the C-terminal region of SAO-1 is required for DLC-1 stability and normal apoptosis.

### The overall structure of the SAO-1-DLC-1 complex

To assess the SAO-1-DLC-1 binding thermodynamics, we subjected the purified C-terminal region (residues 182–226) of SAO-1 and full-length DLC-1 to isothermal titration calorimetry (ITC) and measured their binding affinity. We found that SAO-1 bound to DLC-1 with a *K*_d_ of 0.5 μM (Fig. 4E). We further resolved the complex structure of SAO-1 (residues 182–205) and DLC-1 at 2.4 Å resolution via crystallography (Table S2). Surprisingly, the SAO-1 peptide and DLC-1 formed a 2:4 complex in the crystal structure (Fig. 4F). Each SAO-1 peptide adopted an extended conformation to interact with two DLC-1 dimers and thereby produced two similar DLC-1-binding sites in SAO-1. At each site, the DLC-1-binding sequence in SAO-1 was folded into a β-strand and was pack within a groove formed in the DLC-1 dimerization interface (Fig. 4G), which closely resembled the target-binding modes of other dynein light chain proteins, such as LC8 [33, 34].

### 187VAT189 and 199CQT201 of SAO-1 are required for DLC-1-mediated apoptosis

**Fig. 5:**
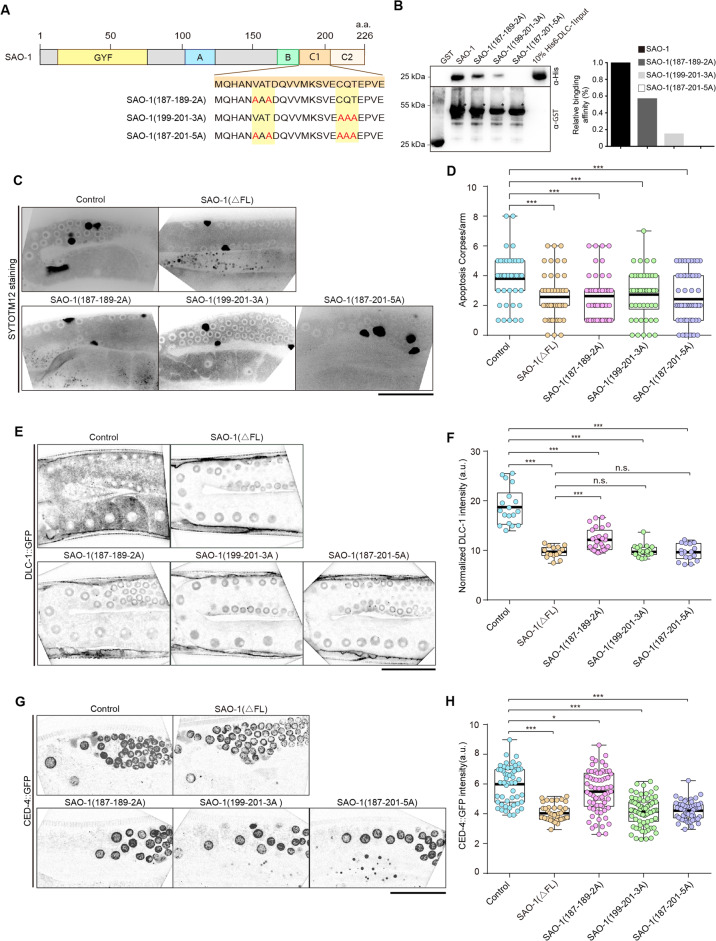
SAO-1 C1 region interacts with DLC-1 and regulates germ cell apoptosis. **A** Schematic of SAO-1 protein domains. Below the protein residue alignment represents the point mutations in mutants. The mutated residue is highlighted with red color. **B** Western blot analysis of DLC-1 binding to purified GST, GST-SAO-1, GST-SAO-1(187-189-2A), GST-SAO-1(199-201-3A), GST-SAO-1(187-201-5A) using His antibody (top). GST-tag fusion proteins were visualized using GST antibody (bottom). Asterisks denote GST-tag fusion proteins. **C** Representative confocal images of apoptotic corpses in control, SAO-1(*Δ*FL), SAO-1(187-189-2A), SAO-1(199-201-3A), and SAO-1(187-201-5A). **D** Quantification of the number of apoptotic corpses in **C**, each dot in the chart represents a single worm. **E** Representative confocal images of DLC-1::GFP in control, SAO-1(*Δ*FL), SAO-1(187-189-2A), SAO-1(199-201-3A), and SAO-1(187-201-5A). **F** Quantification of normalized DLC-1::GFP intensity in **E**, each dot in the chart represents a single worm. **G** Representative confocal images of CED-4::GFP in control, SAO-1(*Δ*FL), SAO-1(187-189-2A), SAO-1(199-201-3A), and SAO-1(187-201-5A). **H** Quantification of normalized CED-4::GFP intensity in **G**, each dot in the chart represents a single nuclear. Data were analyzed by two-tailed Student’s *t* test; Error bars, ±SEM; n.s. not significant. *p < 0.05, ***p < 0.001. a.u. arbitrary unit. Scale bar, 50 μm.

As the complexed structure suggested that SAO-1 bound to DLC-1 at two binding sites, we further investigated whether the two DLC-1-binding sites in SAO-1 are collaborative and necessary for DLC-1 interaction. First, we constructed three GST-labeled SAO-1 proteins with different point mutations in DLC-1-binding sites, SAO-1(187-189-2A), SAO-1(199-201-3A) and SAO-1(187-201-5A) (Fig. 5A) and examined their DLC-1-binding ability via pull-down assay. The DLC-1-binding ability of SAO-1(187-189-2A) and SAO-1(199-201-3A) was partially diminished, while the DLC-1 interaction with SAO-1(187-201-5A) was completed abrogated (Figs. 5B and S5B). These results suggest that the two DLC-1-binding sites in SAO-1 (187VAT189 and 199CQT201) bind each DLC-1 molecule independently.

To further investigate the role of SAO-1 187VAT189 and 199CQT201 at the cellular level, we generated three transgenic strains expressing the following SAO-1 mutations: SAO-1(187-189-2A)::RFP, SAO-1(199-201-3A)::RFP and SAO-1(187-201-5A)::RFP. We first examined the apoptotic behavior of worms expressing each of these three mutants. We found that the number of apoptotic corpses in the germline was significantly reduced in all the mutants (Fig. 5C, D). Next, we examined whether the SAO-1-mutant proteins regulate DLC-1 activity. We co-expressed DLC-1::GFP in the three mutant worm strains and found that the GFP intensities in all the mutant strains were reduced (Fig. 5E, F). The GFP intensity in the SAO-1(187-189-2A)-mutant worms was partially diminished, but the GFP intensity in both the SAO-1(199-201-3A)- and SAO-1(187-201-5A)-mutant worms was suppressed to a level similar to that in the *sao-1(ΔFL)*-mutant worms (Fig. 5E, F). Since nuclear membrane-localized CED-4 was diminished when DLC-1 or SAO-1 was depleted (Fig. 2F, G), we speculated that nuclear membrane-localized CED-4 would also be impacted in SAO-1-mutant proteins with DLC-1-binding site mutations. Consistent with our previous results, the intensity of CED-4::GFP at the nuclear membrane was markedly reduced in SAO-1(187-189-2A)::RFP, SAO-1(199-201-3A)::RFP and SAO-1(187-201-5A)::RFP strains (Fig. 5G, H). Thus, our results indicate that SAO-1 residues 187VAT189 and 199CQT201 are also necessary for DLC-1 interaction in vivo and that normal apoptosis in the *C. elegans* germline requires the SAO-1–DLC-1 complex.

## Discussion

Apoptosis is a highly regulated cell death program in metazoans. The core apoptotic proteins are highly conserved, and their roles have been extensively studied. Once apoptosis activated, several caspase precursors, localized initially in mitochondria, are translocated to the nucleus where they execute their apoptotic functions [2, 35–37]. Specifically, among the core apoptotic proteins, CED-4 translocated from the mitochondrial membrane to the nuclear membrane to activate CED-3 pro-caspase, and induce apoptosis [12, 19, 20]. This translocation requires the binding of CED-4 with CED-9 at the outer mitochondrial membrane. The *ced-4* missense mutant *ced-4(n3040)*, lacking the ability to bind CED-9, displayed diffuse subcellular localization in the cytoplasm and lost the ability to execute apoptosis [12]. However, how CED-4 is recruited to the nucleus remains unclear. A recent report demonstrated that DLC-1 regulated CED-4 nuclear membrane localization in the germ cells of *C. elegans*, and loss of DLC-1 inhibited apoptosis execution [22]. We obtained similar results with the worms with SAO-1 depleted by the standard RNAi-feeding method, as well as those expressing SAO-1 loss-of-function mutations. The number of apoptotic corpses in worms depleted of SAO-1 activity was reduced, and CED-4 nuclear membrane localization was compromised. Furthermore, the enhanced nuclear membrane localization of CED-4 in *ced-9(RNAi)*, with accelerated apoptosis, was partially restored by the loss of SAO-1. These results indicate that SAO-1 functions in the apoptotic pathway. Importantly, the molecular interaction of SAO-1 and DLC-1 was demonstrated by pull-down assays and found to bind in a 2:4 stoichiometry (SAO-1:DLC-1) in an isothermal titration calorimetry analysis.

SAO-1, a suppressor of *aph-1*, contains an N-terminal GYF domain and two low-complexity domains in its mid-region. Although the GYF domain is an established motif known to recognize and bind to target proteins with proline-rich sequences, it was dispensable for the SAO-1-DLC-1 interaction. The GYF domain in SAO-1 is believed to facilitate an intramolecular interaction [38], probably for autoinhibition. However, we determined that a C-terminal region in SAO-1 (C1, residues 182-205) was essential for binding DLC-1. A similar observation has been reported regarding the SAO-1-SEL-10 interaction, in which the SAO-1 C-terminus was necessary for binding SEL-10 [30].

Although dynein light chain proteins are highly conserved across species from human to worms, given the promiscuous interaction between dynein light chain and diverse motifs in DLC-binding partners [39], it is difficult to predict the DLC-1-binding sequence in SAO-1. Nevertheless, in this study, we employed crystallographic tools to resolve the DLC-1-SAO-1 complex structure, and we unexpectedly found two DLC-1-binding sites in SAO-1. Our findings suggest that many dynein light chain-mediated interactions remain to be discovered and that structural biology tool will be very useful in these discoveries.

**Fig. 6:**
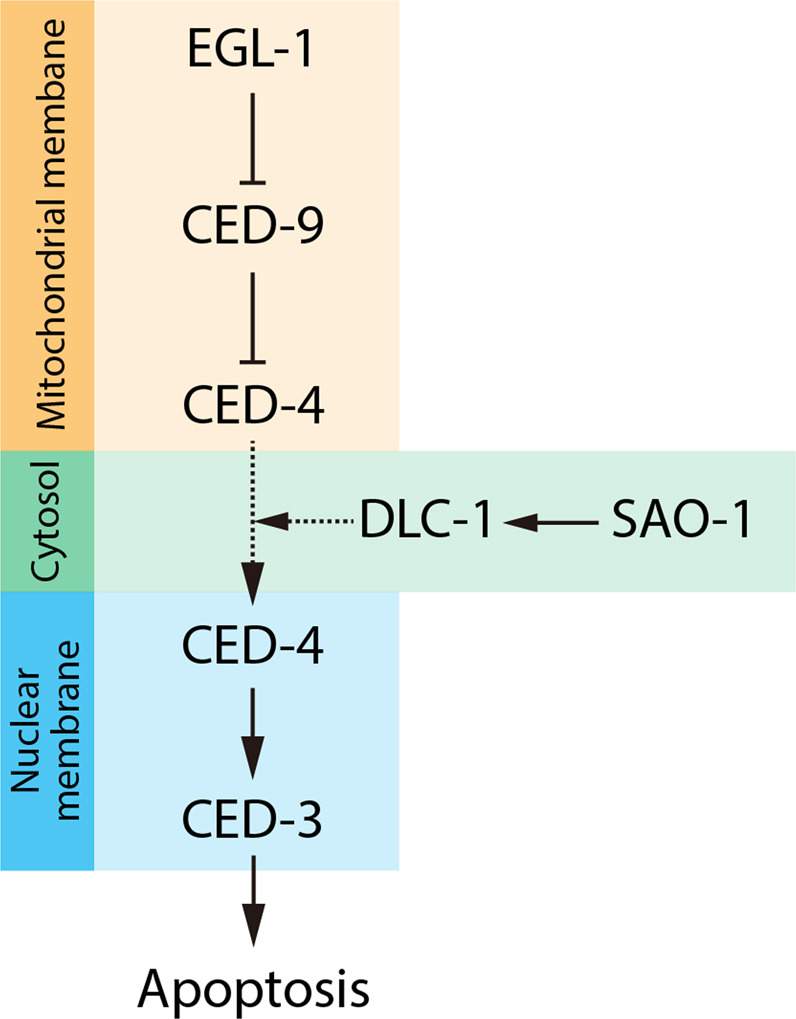
A proposed model: SAO-1 and DLC-1 regulate apoptosis in the germline of *C. elegans*. In non-apoptotic cells, CED-9 is located on the outer mitochondrial membrane and binds to CED-4 [12]. When apoptosis is stimulated, the upstream EGL-1 binds to CED-9 and leads to the release of CED-4 from the mitochondria [12, 17]. In this study, we further demonstrated that SAO-1 interacts with DLC-1 to prevent DLC-1 degradation. Then, DLC-1 promotes the translocation of CED-4 from mitochondria to the nuclear membrane [22]. Finally, CED-4 activates CED-3 and leads to apoptosis [15, 18].

The SAO-1-DLC-1 complex regulates CED-4 nuclear membrane localization. However, there is no evidence to show an association between CED-4 and the SAO-1-DLC-1 complex facilitates CED-4 translocation from mitochondria to the nucleus. Indeed, we obtained results that showed a substantial reduction in cytoplasmic DLC-1 following SAO-1 depletion and in animals that expressed an *sao-1*-mutant lacking the DLC-1 binding region. Furthermore, inhibition of proteasome-mediated degradation via depletion of the 26S proteasomal protein RPT-4 [40] partially rescued DLC-1 levels in *sao-1* mutants. Therefore, we suggest that SAO-1 binds to DLC-1 to prevent DLC-1 degradation and thus maintains an adequate level of DLC-1 for the translocation of CED-4 during apoptosis (Fig. 6). Our in vitro experiments did not indicate an interaction between CED-4 and either SAO-1 or DLC-1. Therefore, the translocation of CED-4 may be indirectly mediated by the SAO-1-DLC-1 complex or DLC-1 alone; further investigation is needed to determine the precise mechanism.

## Materials and methods

### Strains

All *C. elegans* strains were cultured at 22 °C or 25 °C on Nematode Growth Medium (NGM) plates seeded with *Escherichia coli* strain OP50. All strains used in this study are listed in Table S3.

### Transgenic strain construction

The transgenic strains of endogenous *sao-1* (GFP::sao-1::3xFlag, *sao-1*::TagRFP::3xFlag, *sao-1p*::GFP::3xFlag, *sao-1p*::TagRFP::3xFlag, *sao-1p*::BFP::3xFlag, sao-1(187-189-2A)::TagRFP::3xFlag, sao-1(199-201-3A)::TagRFP::3xFlag and sao-1(187-201-5A)::TagRFP::3xFlag) were generated using CRISPR/Cas9-triggered homologous recombination as described previously [31]. For CRISPR/Cas9 knock-in/out, the Cas9–sgRNA was generated by inserting the target sequence into pDD162. The homologous repair template was generated via insertion of the 5’ and 3’ homology arms into pDD282 or pDD284 by recombination; The C-terminal GFP insertion at an endogenous site in cox-5a was generated by SunyBiotech Company [strain name and genotype: PHX969. cox-5A (syb969)]. Both arSi1[*dlc-1p*::dlc-1::GFP cb-unc-119(+)] and opIs219[ced-4p::ced-4::GFP unc-119(+)] stains were kind of gifts form professor Anders Olsen (Department of Chemistry and Biosciences, Aalborg University, Aalborg, Denmark).

The extrachromosomal array strain (*pie-1p*::sao-1::RFP::3Xflag::sao-1 3’UTR) was created by microinjection as follows: The appropriate DNA fragment from N2 genomic DNA was amplified and inserted into modified pCFJ90. The plasmid (50 ng/μl) was then injected into the gonads of young adults. The transgenic worms were screened under the Olympus MVX10 fluorescent microscope. All primers used in this study are listed in Table S4.

### RNA interference studies

RNAi experiments were performed using the feeding method [41]. RNAi plasmids were obtained from the RNAi library (Source Bioscience). HT115 bacteria carrying RNAi plasmids were grown in LB with 100 mg/ml ampicillin at 37 °C overnight and then seeded on the RNAi plates at room temperature for 8 h. For *sao-1, dlc-1* and *ced-9* RNAi, L4-stage worms were placed on the RNAi-feeding plates for 48 h prior to microscopy. For *rpt-4* RNAi, L4-stage worms were placed on the RNAi-feeding plates for 24 h prior to microscopy. To deplete the two genes *(sao-1*and *ced-9, dlc-1* and *ced-9*), the corresponding bacterial strains were mixed at a 1:1 ratio for the combined RNAi experiments.

### Embryonic viability assays

Single day 1-adult hermaphrodite was picked to the new NGM plate and cultured at 22 °C. Then, the number of eggs on the surface of the plate was counted after 12 and 24 h. Embryonic viability = Number of eggs (12 h – 24 h)/12 h. Nine hermaphrodites in each condition were analyzed.

### Live imaging

For germline morphology analysis, young adult worms were immobilized in M9 containing 1% tetramisole, mounted on a 2% agarose pad, covered with a coverslip and sealed with petroleum jelly. They were visualized using Differential Interference Contrast (DIC) microscope (Olympus BX53) equipped with a ×40/1.4 NA objective lens (Olympus) and DP21 camera. Images were acquired using Olympus cellSens Dimension software.

For fluorescent signal imaging, worms were immobilized in M9 containing 1% tetramisole, mounted on a 2% agarose pad, covered with a coverslip and sealed with petroleum jelly. GFP was visualized using 488 nm excitation and 525–550 nm emission and RFP was visualized using 561 nm excitation and 607–636 nm emission filter sets. Images were acquired with either a spinning-disk confocal microscope (Yokogawa CSU-X1) equipped with a ×60/1.4 NA objective lens (Olympus) and a charge-coupled device (CCD) camera, or a laser scanning confocal microscope (Nikon A1R + Symp64) equipped with a ×40/1.4 NA water immersion objective lens (Nikon) and photomultiplier tube (PMT) detector. Images were acquired using MetaMorph software or NLS-Elements AR software respectively.

### Fluorescent intensity analysis

For quantification of CED-4::GFP intensity, germline regions were projected into a single image using the batch z-projection process function in ImageJ (NIH, Bethesda, MD). Then, the intensity was measured by average pixel intensity. Five to eight nuclei with the highest fluorescence intensity in each worm were used to measure the nuclear intensity. The following formula was used to calculate the normalized intensity of nuclear CED-4::GFP: *I*_Normalized_ = (*I*_Nucleus_)/(*I*_Background_).

For quantification of cytoplasmic DLC-1::GFP, the intensity within the ROI (20 × 200 pixels) of the middle plane of the loop region was measured. The following formula was used to calculate the normalized intensity of DLC-1::GFP in cytoplasm: *I*_Normalized_ = (*I*_Cytoplasmic_)/(*I*_Background_).

Nuclear DLC-1::GFP intensity was quantified in nucelei located in the middle plane of the loop region. The following formula was used to calculate the normalized nuclear DLC-1::GFP intensity: *I*_Normalized_ = (*I*_Nucleus_)/(*I*_Background_).

For quantification of muscular DLC-1::GFP intensity, projections of 5 planes maximum at 0.5 μm intervals within muscle cells were captured. The following formula was used to calculate the normalized intensity of muscular DLC-1::GFP: *I*_Normalized_ = (*I*_Muscle_)/(*I*_Background_).

The images in these experiments were analyzed using ImageJ software (National Institutes of Health). The statistical significance was determined using an unpaired two-tailed Student’s *t* test to calculate the *p* values (GraphPad software).

### Programmed cell death assays

For germ cells undergoing apoptosis labeled by CED-1::GFP, L4 worms were grown at 25 °C for 48 h and anesthetized with M9 containing 1% tetramisole. For germ cells undergoing apoptosis stained by SYTO^TM^12, L4 worms were grown at 25 °C for 36 h, and then incubated with 33 µM SYTO^TM^12 green-fluorescent nucleic acid stain (Invitrogen) in M9 for 3 to 4 h. The worms were then transferred to new NGM or RNAi plates to recover for 1 h and then immobilized in M9 containing 1% tetramisole. Afterward, the worms were mounted on a 2% agarose, covered with a coverslip and sealed with petroleum jelly. Images were acquired by spinning-disk confocal microscope (Yokogawa CSU-X1) equipped with a 60x/1.4 NA objective lens (Olympus) and a charge-coupled device (CCD) camera.

### Protein sample preparation

DNA encoding the SAO-1 and the DLC-1 region was PCR-amplified from *C. elegans* N2 cDNA. The DLC-1 coding sequence was cloned into pET32a modified with an N-terminal thioredoxin Trx-His_6_ tag for protein expression. The full-length SAO-1 and SAO-1 mutants were cloned into pGEX-4T-1 vectors containing a GST tag. All point mutations were created using site-directed mutagenesis by PCR and confirmed by sequencing. Protein expression plasmids were transformed into BL21 (DE3) competent cells, and the bacteria were grown in LB with ampicillin (100 μg/ml) at 37 °C. Protein expression was induced by the addition of 0.1 mM IPTG and the bacteria were incubated for an additional 4 to 5 h at 37 °C. GST-tagged proteins were subsequently purified using GST beads. Trx-His_6_-tagged proteins were purified by nickel-affinity chromatography. *C. elegans* protein extracts were prepared from synchronized hermaphrodites (~48 h after the L4 stage) by sonication in PBS buffer (137 mM NaCl, 2.7 mM KCl, 10 mM Na_2_HPO_4_, 2 mM KH_2_PO_4_) supplemented with protease inhibitors Cocktail (Roche). Homogenates were centrifuged at 12,000 × *g* for 10 min at 4 °C. The supernatant was either used immediately for western blotting or snap-froze in liquid nitrogen and stored at –80 °C.

### Pull down assays

Purified Trx-His_6_-tagged protein lysates/worm lysates were incubated with GST-SAO-1, SAO-1 mutant-GST or control GST. The mixture was then incubated at 4 °C for 3 h, and subsequently centrifuged at 500 × *g* for 5 min. The pellet was washed with PBS 3-6 times and then boiled in SDS loading buffer. Western blotting with His antibodies (1:3000, #MA1-21315, Invitrogen) or GST antibodies (1:3000, YM3021, Immuno Way) was conducted according to standard procedures.

### Mass spectroscopy assays

LC-MS/MS analysis was carried out as described previously [42]. Briefly, Wild type N2 worm lysates were immunoprecipitated with GST-SAO-1 or control GST. Each immunoprecipitated sample was separation by SDS-PAGE gel and staining with Brilliant Blue G-250, then destaining with 50 mM ammonium bicarbonate (ABC) and 50% (vol/vol) acetonitrile (ACN) and rehydrated in 100% ACN. Disulfide bonds were reduced with 10 mM dithiothreitol (DTT). Proteins were then alkylated with 100 mM iodoacetamide and 50 mM ammonium bicarbonate and the mixture was incubated with 1 to 2 μl trypsin (Promega) at 37 °C for 16 h. The resulting peptides were desalted with 25 mM ABC, 5% formic acid (FA), 50% ACN and homemade C18-StageTips. The peptides were redissolved in separated 0.1% FA (in water).

Peptide mixtures were analyzed by an Orbitrap Fusion mass spectrometer (Thermo Fisher Scientific) coupled with a nanospray ion source and an EASY-nLC 1000 system (Thermo Fisher Scientific) as described [43]. Full scan MS spectra (*m*/*z* 350–1550) were acquired in the Orbitrap for a maximum injection time of 100 ms at 120,000 resolutions and an AGC target value of 2E5 charges.

### FLIM assays

Adult worms were transferred from a petri dish to a cover slide, immobilized in M9 containing 1% tetramisole, then mounted on a 5% agarose pad and sealed with petroleum jelly. GFP was visualized using a Nikon A1R confocal microscope (Nikon A1R + Symp64) equipped with a ×40/1.4 NA water-immersion lens. The fluorescence lifetime imaging capability was provided by PicoHarp time-correlated single-photon counting (TCSPC) electronics. The donor was excited with a 510 nm laser at 20 MHz pulse frequency, and the fluorescence emission of GFP fusion proteins was collected using a 550/49 nm filter. Fluorescence lifetimes were analyzed by SymphoTime64 v2.0 (PicoQuant). The value was considered valid when the chi-squared values were 0.9–1.5.

### Isothermal titration calorimetry (ITC) assays

ITC experiments were performed on a PEAQ-ITC Microcal calorimeter (Malvern) at 25 °C. Protein samples (in 50 mM Tris-Cl, pH 7.5, 100 mM NaCl) were prepared for titrations. Each measurement included 13 titrations. In total, 400 μM DLC-1 was injected into the sample well containing 40 μM SAO-1 protein with a titration speed of 0.5 ml/s.

A time interval of 150 s between injections was used to ensure that the titration peak returned to the baseline. The titration data were processed by MicroCal PEAQ-ITC analysis software and fit by the one-site binding model.

### Crystallography

Crystals of SAO-1/DLC-1 complex was obtained by the sitting drop vapor diffusion method at 16 °C. To set up a sitting drop, concentrated protein solution (1 μl, ~15.5 mg/ml,) was mixed with crystallization solution (1 μl, 0.2 M Potassium chloride, 0.05 M HEPES pH 7.5, 35% (v/v) Pentaerythritol propoxylate). Glycerol (30%) was added into the crystallization solution as the cryo-protectant before X-ray diffraction. The X-ray diffraction data were collected at Shanghai Synchrotron Radiation Facility beamlines BL17U1 [44], BL18U1 and BL19U1 [45], and indexed and scaled by the HKL2000 software package [46].

The complex structure was determined by molecular replacement in PHASER [47] using the human dynein light chain structure (PDB id: 3P8M) as the search model. The structural model was refined in PHENIX [48] and adjusted in COOT [49]. The model quality was checked by MolProbity [50]. Final refinement statistics are listed in Table S2. Figures for all structure were prepared using PyMOL (https://www.pymol.org).

## Supplementary information

Supplemental Tables

Supplemental Figures

Original Data File

## Data Availability

All the data are available in the article and Supplementary Files.
