# Supplementary material for: Interaction between DLC-1 and SAO-1 facilitates CED-4 translocation during apoptosis in the Caenorhabditis elegans germline
Source: Cell Death Discov. 2022 Nov 3;8:441. doi: 10.1038/s41420-022-01233-9 (PMC9630320; doi:10.1038/s41420-022-01233-9)
Supplement: Supplementary file 1 — Supplemental Tables [file 41420_2022_1233_MOESM1_ESM.pdf]

Table S1. Mass spectrometry analysis data of SAO-1.

Accession	Description	Score	Coverage	Protein	Unique Peptic	Peptide	# PSMs	# AAs	MW [kDa]	calc. pI
P18947	Vitellogenin-4 OS=Caenorhabditis elegans GN=vit-4 PE=1 SV=3 - [VIT4_CAEEL]	166.65	34.25	4	3	57	66	1603	186.2	7.01
P50880	60S ribosomal protein L3 OS=Caenorhabditis elegans GN=rpl-3 PE=2 SV=1 - [RL3_CAEEL]	80.72	35.41	3	17	17	30	401	45.6	10.40
Q9U302	Polyadenylate-binding protein OS=Caenorhabditis elegans GN=pab-1 PE=1 SV=1 - [Q9U302_CAEEL]	72.95	34.06	3	17	21	25	646	71.6	9.03
Q8MXJ1	Uncharacterized protein OS=Caenorhabditis elegans GN=CELE_T23E7.2 PE=1 SV=1 - [Q8MXJ1_CAEEL]	61.75	27.09	2	1	18	21	908	98.2	4.34
Q17832	ViGiLN homolog OS=Caenorhabditis elegans GN=vgln-1 PE=1 SV=2 - [Q17832_CAEEL]	60.66	21.31	2	21	22	24	1220	134.3	6.80
P91374	60S ribosomal protein L15 OS=Caenorhabditis elegans GN=rpl-15 PE=3 SV=1 - [RL15_CAEEL]	53.93	43.14	1	11	11	18	204	24.1	11.63
O18688	Tubulin alpha chain OS=Caenorhabditis elegans GN=tba-1 PE=1 SV=1 - [O18688_CAEEL]	53.89	35.19	7	1	14	20	449	50.0	5.05
P40614	Phosphate carrier protein, mitochondrial OS=Caenorhabditis elegans GN=F01G4.6 PE=2 SV=1 - [MPCP_CAEEL]	48.60	39.41	2	12	12	17	340	36.6	8.95
P27604	Adenosylhomocysteinase OS=Caenorhabditis elegans GN=ahcy-1 PE=3 SV=1 - [SAHH_CAEEL]	47.22	33.64	1	14	14	16	437	47.5	6.25
Q9XU13	Calcium-transporting ATPase OS=Caenorhabditis elegans GN=sca-1 PE=1 SV=1 - [Q9XU13_CAEEL]	45.14	21.61	3	18	18	18	1004	109.6	5.12
Q22866	Tropomyosin isoforms a/b/d/f OS=Caenorhabditis elegans GN=lev-11 PE=1 SV=1 - [TPM1_CAEEL]	43.31	40.14	15	10	10	15	284	33.0	4.69
Q19020	Dynein heavy chain, cytoplasmic OS=Caenorhabditis elegans GN=dhc-1 PE=1 SV=1 - [DYHC_CAEEL]	41.83	5.17	2	22	22	22	4568	521.2	6.27
P12456	Tubulin beta-1 chain OS=Caenorhabditis elegans GN=mec-7 PE=2 SV=1 - [TBB1_CAEEL]	41.47	20.63	1	2	9	18	441	49.2	5.01
Q94300	60S ribosomal protein L11-1 OS=Caenorhabditis elegans GN=rpl-11.1 PE=3 SV=1 - [RL111_CAEEL]	40.50	27.55	2	1	5	14	196	22.7	9.99
O17680	Probable S-adenosylmethionine synthase 1 OS=Caenorhabditis elegans GN=sams-1 PE=1 SV=1 - [METK1_CAEEL]	40.03	33.75	1	10	12	16	403	43.6	6.48
Q17770	Protein disulfide-isomerase 2 OS=Caenorhabditis elegans GN=pdi-2 PE=1 SV=1 - [PDI2_CAEEL]	39.54	28.40	3	12	12	15	493	55.1	4.79
Q21355	Glutathione S-transferase 4 OS=Caenorhabditis elegans GN=gst-4 PE=2 SV=1 - [GST4_CAEEL]	37.30	47.34	1	10	10	12	207	23.9	5.73
H2L2J6	Prion-like-(Q/N-rich)-domain-bearing protein OS=Caenorhabditis elegans GN=pqn-27 PE=1 SV=1 - [H2L2J6_CAEEL]	36.66	26.01	3	8	8	12	373	42.0	4.68
P49180	60S ribosomal protein L35a OS=Caenorhabditis elegans GN=rpl-33 PE=1 SV=3 - [RL35A_CAEEL]	36.09	48.39	1	8	8	10	124	13.8	11.25
G5EFU2	Adenine Nucleotide Translocator OS=Caenorhabditis elegans GN=ant-1.4 PE=1 SV=1 - [G5EFU2_CAEEL]	31.44	24.60	3	1	8	11	313	34.3	9.77
P48150	40S ribosomal protein S14 OS=Caenorhabditis elegans GN=rps-14 PE=3 SV=1 - [RS14_CAEEL]	30.14	47.37	1	7	7	9	152	16.2	10.40
Q9XW17	Cytokinesis, Apoptosis, RNA-associated OS=Caenorhabditis elegans GN=car-1 PE=1 SV=1 - [Q9XW17_CAEEL]	29.10	29.41	1	9	9	9	340	37.6	9.17
Q9TYV5	NucleOLar protein OS=Caenorhabditis elegans GN=nol-1 PE=1 SV=2 - [Q9TYV5_CAEEL]	28.32	16.72	2	9	9	12	664	73.9	9.13
Q9N4I4	60S ribosomal protein L10a OS=Caenorhabditis elegans GN=rpl-10a PE=3 SV=1 - [RL10A_CAEEL]	27.34	35.19	2	6	6	8	216	24.1	9.92
D0IMZ7	FiLamiN (Actin binding protein) homolog OS=Caenorhabditis elegans GN=fln-1 PE=1 SV=1 - [D0IMZ7_CAEEL]	27.23	6.40	19	11	11	11	2204	240.4	6.34
Q9TZL8	ATP-dependent 6-phosphofructokinase 1 OS=Caenorhabditis elegans GN=pfk-1.1 PE=3 SV=2 - [PFKA1_CAEEL]	26.63	12.16	1	7	7	9	814	89.7	6.68
O18650	40S ribosomal protein S19 OS=Caenorhabditis elegans GN=rps-19 PE=2 SV=1 - [RS19_CAEEL]	25.62	38.36	1	6	6	9	146	16.3	10.29
O76360	cGMP-dependent protein kinase egl-4 OS=Caenorhabditis elegans GN=egl-4 PE=1 SV=2 - [EGL4_CAEEL]	25.55	14.87	8	10	10	10	780	86.7	7.81
P91128	60S ribosomal protein L13 OS=Caenorhabditis elegans GN=rpl-13 PE=3 SV=1 - [RL13_CAEEL]	25.49	38.65	1	8	8	10	207	23.7	11.09
Q95XP6	Calcium-transporting ATPase OS=Caenorhabditis elegans GN=mca-3 PE=1 SV=1 - [Q95XP6_CAEEL]	25.43	10.60	5	10	10	10	1160	126.9	5.78
P91917	Obg-like ATPase 1 OS=Caenorhabditis elegans GN=ola-1 PE=3 SV=1 - [OLA1_CAEEL]	25.24	33.16	2	11	11	11	395	44.3	6.89
Q95XJ0	Uncharacterized protein OS=Caenorhabditis elegans GN=CELE_Y69A2AR.18 PE=1 SV=1 - [Q95XJ0_CAEEL]	24.61	27.09	3	7	7	9	299	32.4	9.04
Q27389	60S ribosomal protein L13a OS=Caenorhabditis elegans GN=rpl-16 PE=2 SV=1 - [RL13A_CAEEL]	24.35	32.67	1	7	7	9	202	23.0	10.76
Q19264	Putative deoxyribose-phosphate aldolase OS=Caenorhabditis elegans GN=F09E5.3 PE=3 SV=1 - [DEOC_CAEEL]	23.61	37.95	1	10	10	10	303	33.1	7.40
Q19579	Polyadenylate-binding protein OS=Caenorhabditis elegans GN=pab-2 PE=1 SV=1 - [Q19579_CAEEL]	22.84	15.90	2	4	8	8	692	75.9	9.07
Q9NAB2	Glutathione S-Transferase OS=Caenorhabditis elegans GN=gst-28 PE=1 SV=1 - [Q9NAB2_CAEEL]	22.42	19.62	3	1	4	10	209	23.6	5.68
P34255	Uncharacterized protein B0303.3 OS=Caenorhabditis elegans GN=B0303.3 PE=3 SV=1 - [YKA3_CAEEL]	22.31	20.76	1	9	9	9	448	47.8	9.07
Q9NEN6	40S ribosomal protein S6 OS=Caenorhabditis elegans GN=rps-6 PE=1 SV=1 - [RS6_CAEEL]	21.07	30.08	3	7	7	10	246	28.1	10.26
H9G333	CathePsin L family OS=Caenorhabditis elegans GN=cpl-1 PE=1 SV=1 - [H9G333_CAEEL]	20.89	41.41	2	6	6	8	198	21.8	5.74
Q565D2	Uncharacterized protein OS=Caenorhabditis elegans GN=CELE_F49E2.5 PE=1 SV=1 - [Q565D2_CAEEL]	20.80	12.14	11	8	8	8	1030	113.6	8.56
Q21086	Guanine nucleotide-binding protein-like 3 homolog OS=Caenorhabditis elegans GN=nst-1 PE=3 SV=1 - [GNL3_CAEEL]	18.61	16.19	1	9	9	9	556	62.3	9.36
Q9NA98	Actin-Related Proteins OS=Caenorhabditis elegans GN=arp-1 PE=1 SV=2 - [Q9NA98_CAEEL]	18.44	20.59	2	6	6	7	374	42.0	6.74
G5EDH9	Plant Late Embryo Abundant (LEA) related OS=Caenorhabditis elegans GN=lea-1 PE=1 SV=1 - [G5EDH9_CAEEL]	18.22	7.64	16	8	8	8	1126	118.4	4.86
Q09450	uccinyl-CoA:3-ketoacid coenzyme A transferase, mitochondrial OS=Caenorhabditis elegans GN=C05C10.3 PE=3 SV=1 - [SCC	17.89	15.36	1	7	7	7	521	56.1	8.22
Q22137	Uncharacterized protein OS=Caenorhabditis elegans GN=CELE_T04A8.7 PE=1 SV=1 - [Q22137_CAEEL]	17.76	15.71	2	8	8	12	681	78.6	6.15
Q27481	UBA (Human ubiquitin) related OS=Caenorhabditis elegans GN=uba-1 PE=1 SV=1 - [Q27481_CAEEL]	17.14	10.24	3	11	11	11	1113	124.0	5.21
Q9BKU5	Uncharacterized protein OS=Caenorhabditis elegans GN=CELE_Y37E3.8 PE=1 SV=1 - [Q9BKU5_CAEEL]	16.97	34.48	2	5	5	6	145	16.2	10.55
P91910	Tubulin alpha-3 chain OS=Caenorhabditis elegans GN=mec-12 PE=1 SV=1 - [TBA3_CAEEL]	16.93	13.56	5	1	7	9	450	50.1	5.15
Q23629	utarate dehydrogenase E1 component DHKTD1 homolog, mitochondrial OS=Caenorhabditis elegans GN=ogdh-2 PE=3 SV=4 -	16.81	7.57	2	5	5	6	911	102.7	6.46
Q19680	Phosphoacetylglucosamine mutase OS=Caenorhabditis elegans GN=CELE_F21D5.1 PE=1 SV=1 - [Q19680_CAEEL]	16.33	12.73	1	5	5	6	550	61.0	5.48
Q21000	MYOsin heavy chain structural genes OS=Caenorhabditis elegans GN=myo-5 PE=1 SV=1 - [Q21000_CAEEL]	16.24	2.48	1	2	5	8	1974	226.9	6.21
B5BM23	Initiation Factor 4G (eIF4G) family OS=Caenorhabditis elegans GN=ifg-1 PE=1 SV=1 - [B5BM23_CAEEL]	16.19	9.79	5	6	6	8	1042	116.0	6.74
Q21902	DNA replication licensing factor mcm-5 OS=Caenorhabditis elegans GN=mcm-5 PE=3 SV=1 - [MCM5_CAEEL]	16.00	12.12	1	7	7	7	759	84.9	8.81
Q23089	EXPOrtin (Nuclear export receptor) OS=Caenorhabditis elegans GN=xpo-1 PE=1 SV=2 - [Q23089_CAEEL]	15.86	4.91	2	4	4	5	1080	123.8	5.25
Q22099	Lysine--tRNA ligase OS=Caenorhabditis elegans GN=krs-1 PE=3 SV=1 - [SYK_CAEEL]	15.55	12.76	3	6	6	6	572	65.1	6.06

G5EBJ7	Fructose-1,6-BiPhosphatase OS=Caenorhabditis elegans GN=fbp-1 PE=1 SV=1 - [G5EBJ7_CAEEL]	15.55	18.48	1	5	5	5	341	37.2	5.59
Q19877	40S ribosomal protein S23 OS=Caenorhabditis elegans GN=rps-23 PE=3 SV=1 - [RS23_CAEEL]	15.24	23.78	1	3	3	5	143	15.9	10.48
Q95QM8	tamine-Fructose 6-phosphate AminoTransferase homolog OS=Caenorhabditis elegans GN=gfat-1 PE=1 SV=1 - [Q95QM8_CAE	14.94	8.71	2	1	4	5	712	79.5	6.38
Q18066	Disorganized muscle protein 1 OS=Caenorhabditis elegans GN=dim-1 PE=1 SV=3 - [DIM_CAEEL]	14.67	8.91	1	4	4	5	640	71.8	7.96
Q20363	Stress-induced protein 1 OS=Caenorhabditis elegans GN=sip-1 PE=1 SV=1 - [SIP1_CAEEL]	14.65	26.42	1	3	3	4	159	17.8	8.09
P54811	ransitional endoplasmic reticulum ATPase homolog 1 OS=Caenorhabditis elegans GN=cdc-48.1 PE=1 SV=1 - [TERA1_CAEEL	14.26	7.29	1	1	4	5	809	89.7	5.33
P46563	Fructose-bisphosphate aldolase 2 OS=Caenorhabditis elegans GN=aldo-2 PE=2 SV=1 - [ALF2_CAEEL]	14.19	19.40	2	5	5	5	366	38.8	7.88
Q93619	anched Chain Keto acid Dehydrogenase e1 (E1) subunit OS=Caenorhabditis elegans GN=bckd-1b PE=1 SV=1 - [Q93619_CAE	14.14	20.77	1	4	4	4	366	40.0	6.68
Q19328	Tudor Staphylococcal Nuclease homolog OS=Caenorhabditis elegans GN=tsn-1 PE=1 SV=1 - [Q19328_CAEEL]	13.82	9.85	1	7	7	7	914	100.7	7.75
Q9BI73	Uncharacterized protein OS=Caenorhabditis elegans GN=CELE_R151.2 PE=1 SV=3 - [Q9BI73_CAEEL]	13.76	19.75	4	5	5	5	319	34.8	6.86
G5EDE8	ERGIC-53-like protein OS=Caenorhabditis elegans GN=ile-1 PE=1 SV=1 - [G5EDE8_CAEEL]	13.66	15.04	1	6	6	6	492	54.9	6.55
Q18074	Uncharacterized protein OS=Caenorhabditis elegans GN=C18B2.3 PE=1 SV=2 - [Q18074_CAEEL]	13.61	16.95	1	3	3	4	236	26.6	10.14
P34540	Kinesin heavy chain OS=Caenorhabditis elegans GN=unc-116 PE=2 SV=2 - [KINH_CAEEL]	13.02	10.43	2	7	7	7	815	91.8	6.40
O01504	60S acidic ribosomal protein P2 OS=Caenorhabditis elegans GN=C37A2.7 PE=3 SV=2 - [RLA2_CAEEL]	12.87	30.84	1	2	2	3	107	10.8	4.68
O17570	60S ribosomal protein L38 OS=Caenorhabditis elegans GN=rpl-38 PE=3 SV=2 - [RL38_CAEEL]	12.81	35.71	1	2	2	4	70	8.1	10.05
O17694	RNAi-Induced Longevity OS=Caenorhabditis elegans GN=ril-1 PE=1 SV=1 - [O17694_CAEEL]	12.80	25.90	1	3	3	4	139	16.5	9.54
Q22021	Putative ATP synthase subunit f, mitochondrial OS=Caenorhabditis elegans GN=R53.4 PE=3 SV=1 - [ATPK_CAEEL]	12.77	34.64	1	5	5	6	153	18.7	10.04
Q86S66	Nascent polypeptide-associated complex subunit alpha OS=Caenorhabditis elegans GN=icd-2 PE=1 SV=1 - [NACA_CAEEL]	12.74	20.30	2	3	3	4	197	22.1	5.06
G5EEP7	Uncharacterized protein OS=Caenorhabditis elegans GN=CELE_T01D3.6 PE=1 SV=1 - [G5EEP7_CAEEL]	12.45	8.52	2	5	5	5	915	102.2	5.07
Q27493	DNA-directed RNA polymerase subunit beta OS=Caenorhabditis elegans GN=rpoa-2 PE=1 SV=1 - [Q27493_CAEEL]	12.43	5.68	1	5	5	5	1127	127.0	8.16
O16297	DNA helicase OS=Caenorhabditis elegans GN=mcm-7 PE=1 SV=1 - [O16297_CAEEL]	12.40	7.95	1	5	5	6	730	81.6	6.38
O45430	MethylCrotonoyl-Coenzyme A Carboxylase (Alpha) OS=Caenorhabditis elegans GN=mccc-1 PE=1 SV=2 - [O45430_CAEEL]	12.37	8.20	1	4	4	5	671	73.7	6.47
G5EFG0	SEL-1 OS=Caenorhabditis elegans GN=sel-1 PE=1 SV=1 - [G5EFG0_CAEEL]	12.33	11.53	1	5	5	5	685	76.2	8.09
A7LPE6	Glycerol-3-phosphate dehydrogenase [NAD(+)] OS=Caenorhabditis elegans GN=gpdh-2 PE=1 SV=1 - [A7LPE6_CAEEL]	12.21	16.67	4	4	4	4	360	39.3	6.46
G5ED21	DNA-directed RNA polymerase subunit OS=Caenorhabditis elegans GN=rpoa-1 PE=1 SV=1 - [G5ED21_CAEEL]	12.20	4.72	1	6	6	6	1737	195.0	7.99
Q86NB3	Phosphoethanolamine MethylTransferase OS=Caenorhabditis elegans GN=pmt-1 PE=1 SV=1 - [Q86NB3_CAEEL]	12.16	11.30	3	4	4	4	460	53.5	5.64
G5EBP5	Uncharacterized protein OS=Caenorhabditis elegans GN=CELE_ZC247.1 PE=1 SV=1 - [G5EBP5_CAEEL]	12.11	20.60	1	3	3	4	4389	496.1	5.17
Q22799	Dynein light chain 1, cytoplasmic OS=Caenorhabditis elegans GN=dlc-1 PE=1 SV=1 - [DYL1_CAEEL]	12.04	24.72	1	2	2	4	89	10.3	7.37
V6CLP2	Uncharacterized protein OS=Caenorhabditis elegans GN=CELE_Y48G8AL.5 PE=1 SV=1 - [V6CLP2_CAEEL]	11.99	10.54	4	4	4	4	484	55.3	6.64
S5ZDJ9	NTL-1b OS=Caenorhabditis elegans GN=let-711 PE=1 SV=1 - [S5ZDJ9_CAEEL]	11.94	5.23	5	7	7	8	1875	208.5	7.75
Q9TYJ7	Dolichol Phosphate Mannosyltransferase OS=Caenorhabditis elegans GN=dpm-1 PE=1 SV=2 - [Q9TYJ7_CAEEL]	11.67	23.01	2	4	4	4	239	26.9	8.88
Q23050	CaLponIn-liKe proteins OS=Caenorhabditis elegans GN=clik-1 PE=1 SV=1 - [Q23050_CAEEL]	11.62	13.30	2	4	4	4	406	44.3	5.39
Q9U2H9	Probable elongation factor 1-beta/1-delta 2 OS=Caenorhabditis elegans GN=eef-1B.2 PE=1 SV=4 - [EF1B2_CAEEL]	11.60	20.53	1	2	4	4	263	28.2	5.05
P46550	T-complex protein 1 subunit zeta OS=Caenorhabditis elegans GN=cct-6 PE=1 SV=1 - [TCPZ_CAEEL]	11.57	10.95	2	5	5	5	539	58.9	6.28
G5EDZ2	Uridine 5'-monophosphate synthase OS=Caenorhabditis elegans GN=umps-1 PE=1 SV=1 - [UMPS_CAEEL]	11.31	11.07	1	4	4	4	497	54.8	5.99
Q9XXN2	MICOS complex subunit MIC60-2 OS=Caenorhabditis elegans GN=immt-2 PE=2 SV=2 - [IMMT2_CAEEL]	10.84	9.48	1	5	5	5	654	72.6	9.36
G4SPY1	Uncharacterized protein OS=Caenorhabditis elegans GN=CELE_Y71G10AL.1 PE=1 SV=2 - [G4SPY1_CAEEL]	10.73	8.14	2	5	5	5	676	75.6	5.78
A0A061AL89	Uncharacterized protein OS=Caenorhabditis elegans GN=CELE_R11A8.7 PE=1 SV=1 - [A0A061AL89_CAEEL]	10.63	3.54	3	6	6	6	2602	285.2	6.80
G5EE96	Oxoglutarate/malate carrier protein OS=Caenorhabditis elegans GN=slc-25a10 PE=1 SV=1 - [G5EE96_CAEEL]	10.59	16.21	1	5	5	5	290	32.0	9.63
Q23670	Probable DNA topoisomerase 2 OS=Caenorhabditis elegans GN=K12D12.1 PE=3 SV=2 - [TOP2_CAEEL]	10.59	4.41	2	6	6	6	1520	172.2	8.51
Q966G8	Glutathione S-Transferase OS=Caenorhabditis elegans GN=gst-41 PE=1 SV=1 - [Q966G8_CAEEL]	10.49	16.19	1	4	4	4	210	25.0	6.57
Q9TZ33	Ubiquinol-Cytochrome c oxidoReductase complex OS=Caenorhabditis elegans GN=ucr-2.3 PE=1 SV=1 - [Q9TZ33_CAEEL]	10.45	10.07	1	3	3	4	427	45.5	8.88
O45502	DNaJ domain (Prokaryotic heat shock protein) OS=Caenorhabditis elegans GN=dnj-12 PE=1 SV=1 - [O45502_CAEEL]	10.32	11.94	1	4	4	4	402	44.3	6.48
P53014	Myosin, essential light chain OS=Caenorhabditis elegans GN=mlc-3 PE=4 SV=1 - [MLE_CAEEL]	10.28	16.99	1	2	2	3	153	17.1	4.70
P41988	T-complex protein 1 subunit alpha OS=Caenorhabditis elegans GN=cct-1 PE=3 SV=2 - [TCPA_CAEEL]	10.24	8.74	1	4	4	4	549	58.8	6.49
C9IY22	Uncharacterized protein OS=Caenorhabditis elegans GN=CELE_ZK1058.9 PE=1 SV=1 - [C9IY22_CAEEL]	9.98	22.77	2	4	4	4	224	26.7	9.74
Q65ZB8	Acetyl-coenzyme A synthetase OS=Caenorhabditis elegans GN=acs-19 PE=1 SV=1 - [Q65ZB8_CAEEL]	9.91	7.46	2	4	4	4	670	73.3	6.18
Q8IG31	PeRoxireDoXin OS=Caenorhabditis elegans GN=prdx-2 PE=1 SV=1 - [Q8IG31_CAEEL]	9.88	15.90	4	3	3	4	195	21.8	5.83
G5EEM5	Zygote defective protein 9 OS=Caenorhabditis elegans GN=zyg-9 PE=1 SV=1 - [ZYG9_CAEEL]	9.83	6.43	1	7	7	7	1415	156.1	7.56
Q09517	Very-long-chain 3-oxooacyl-coA reductase let-767 OS=Caenorhabditis elegans GN=let-767 PE=1 SV=2 - [LE767_CAEEL]	9.80	12.34	5	3	3	3	316	34.3	9.51
P48166	Ribosomal protein rpl-41 OS=Caenorhabditis elegans GN=rpl-41 PE=3 SV=2 - [RL44_CAEEL]	9.79	21.90	1	3	3	3	105	12.4	10.76
Q95XT5	TRanslocon-Associated Protein OS=Caenorhabditis elegans GN=trap-1 PE=1 SV=1 - [Q95XT5_CAEEL]	9.70	15.95	1	4	4	4	257	28.6	6.16
Q93873	ADH dehydrogenase [ubiquinone] iron-sulfur protein 2, mitochondrial OS=Caenorhabditis elegans GN=gas-1 PE=3 SV=2 - [NDU	9.65	8.51	2	4	4	4	482	54.5	6.49
Q95Y89	Pescadillo homolog OS=Caenorhabditis elegans GN=lpd-7 PE=3 SV=1 - [PESC_CAEEL]	9.45	9.60	1	4	4	4	531	61.6	9.42
O62512	Uncharacterized protein OS=Caenorhabditis elegans GN=CELE_ZK550.3 PE=1 SV=3 - [O62512_CAEEL]	9.45	5.83	1	4	4	4	772	90.3	6.57
G5EBF3	Protein Up-regulated in Daf-2(Gf) OS=Caenorhabditis elegans GN=pud-2.1 PE=1 SV=1 - [G5EBF3_CAEEL]	9.43	25.97	1	4	4	4	154	17.4	6.52
Q9U2D9	Glycogen [starch] synthase OS=Caenorhabditis elegans GN=gsy-1 PE=1 SV=1 - [GYS_CAEEL]	9.41	5.51	1	4	4	5	672	76.4	6.25

Q95008	Proteasome subunit alpha type-5 OS=Caenorhabditis elegans GN=pas-5 PE=1 SV=1 - [PSA5_CAEEL]	9.24	17.74	1	4	4	4	248	27.2	5.40
Q9BMU4	ATLastiN (Endoplasmic reticulum GTPase) related OS=Caenorhabditis elegans GN=atln-1 PE=1 SV=1 - [Q9BMU4_CAEEL]	9.23	6.98	4	3	3	3	573	64.7	5.57
S6EZK7	Uncharacterized protein OS=Caenorhabditis elegans GN=CELE_F11C1.5 PE=1 SV=1 - [S6EZK7_CAEEL]	8.96	4.52	5	4	4	4	974	110.5	7.39
Q27257	Protein dif-1 OS=Caenorhabditis elegans GN=dif-1 PE=2 SV=1 - [DIF1_CAEEL]	8.96	17.63	1	3	3	3	312	33.1	8.90
P53489	Actin-related protein 2 OS=Caenorhabditis elegans GN=arx-2 PE=3 SV=1 - [ARP2_CAEEL]	8.95	13.16	1	4	4	4	395	44.8	6.61
P34525	Probable signal peptidase complex subunit 3 OS=Caenorhabditis elegans GN=K12H4.4 PE=1 SV=1 - [SPCS3_CAEEL]	8.85	21.11	1	3	3	3	180	20.7	8.57
A7DT40	Uncharacterized protein OS=Caenorhabditis elegans GN=CELE_F49D11.10 PE=1 SV=2 - [A7DT40_CAEEL]	8.80	7.84	1	5	5	5	727	79.9	7.14
Q9GUF2	ACid Phosphatase family OS=Caenorhabditis elegans GN=acp-6 PE=1 SV=2 - [Q9GUF2_CAEEL]	8.75	10.24	1	4	4	4	381	43.0	8.38
Q09657	Uncharacterized protein OS=Caenorhabditis elegans GN=CELE_ZK1320.9 PE=1 SV=1 - [Q09657_CAEEL]	8.67	9.11	1	3	3	3	472	51.3	8.62
P34556	Cyclin-dependent kinase 1 OS=Caenorhabditis elegans GN=cdk-1 PE=1 SV=1 - [CDK1_CAEEL]	8.59	16.27	21	5	5	5	332	38.3	6.27
Q18036	Uncharacterized protein OS=Caenorhabditis elegans GN=C16A3.5 PE=1 SV=1 - [Q18036_CAEEL]	8.54	33.13	1	4	4	4	163	19.3	8.27
G4SRS5	ABC Transporter, Mitochondrial OS=Caenorhabditis elegans GN=abtm-1 PE=1 SV=1 - [G4SRS5_CAEEL]	8.50	5.68	2	4	4	4	704	77.4	9.16
Q9NAH6	Ribosomal protein S6 kinase beta OS=Caenorhabditis elegans GN=rsks-1 PE=1 SV=2 - [KS6B_CAEEL]	8.49	6.91	1	3	3	3	550	60.4	5.94
Q9BPN6	Mitochondrial Ribosomal Protein, Large OS=Caenorhabditis elegans GN=mrpl-15 PE=1 SV=1 - [Q9BPN6_CAEEL]	8.44	13.09	1	4	4	4	298	33.8	9.22
Q86NJ8	Piwi-like protein OS=Caenorhabditis elegans GN=ppw-1 PE=1 SV=1 - [Q86NJ8_CAEEL]	8.40	5.86	5	4	4	4	887	99.5	9.14
Q18598	Peroxisomal Membrane Protein related OS=Caenorhabditis elegans GN=pmp-2 PE=1 SV=1 - [Q18598_CAEEL]	8.32	5.90	1	3	3	3	661	74.8	9.07
Q09545	te dehydrogenase [ubiquinone] iron-sulfur subunit, mitochondrial OS=Caenorhabditis elegans GN=sdhb-1 PE=2 SV=1 - [SDHB_	8.27	11.41	1	3	3	4	298	32.9	8.24
Q20306	ABC transporter, class F OS=Caenorhabditis elegans GN=abcf-3 PE=1 SV=1 - [Q20306_CAEEL]	8.13	4.21	1	3	3	3	712	80.2	6.43
O02267	Uncharacterized protein OS=Caenorhabditis elegans GN=CELE_F45H10.3 PE=1 SV=1 - [O02267_CAEEL]	8.11	30.95	1	4	4	4	168	18.4	9.77
P91027	Calponin Homology Domain containing Protein OS=Caenorhabditis elegans GN=chdp-1 PE=1 SV=1 - [P91027_CAEEL]	8.09	14.20	1	3	3	3	338	35.9	4.83
G5EGP8	CathePsin Z OS=Caenorhabditis elegans GN=cpz-1 PE=1 SV=1 - [G5EGP8_CAEEL]	8.04	7.52	2	2	2	3	306	34.7	6.87
Q9N584	Uncharacterized protein OS=Caenorhabditis elegans GN=let-630 PE=1 SV=1 - [Q9N584_CAEEL]	8.01	5.76	1	3	3	4	660	74.8	7.05
H2L001	Heat Shock Protein OS=Caenorhabditis elegans GN=hsp-75 PE=1 SV=1 - [H2L001_CAEEL]	7.98	7.72	2	3	3	3	479	54.2	8.97
O61199	2-oxoglutarate dehydrogenase, mitochondrial OS=Caenorhabditis elegans GN=ogdh-1 PE=1 SV=2 - [ODO1_CAEEL]	7.87	5.73	2	4	4	4	1029	115.6	6.80
D0PV95	ATP-dependent RNA helicase laf-1 OS=Caenorhabditis elegans GN=laf-1 PE=1 SV=1 - [DDX3_CAEEL]	7.83	5.08	3	2	3	3	708	76.3	7.06
P50306	Probable S-adenosylmethionine synthase 4 OS=Caenorhabditis elegans GN=sams-4 PE=1 SV=1 - [METK4_CAEEL]	7.78	12.13	3	2	4	4	404	43.9	6.32
Q21554	DNA damage-binding protein 1 OS=Caenorhabditis elegans GN=ddb-1 PE=1 SV=2 - [DDB1_CAEEL]	7.69	3.17	1	3	3	3	1134	125.6	5.30
Q09936	Uncharacterized protein C53C9.2 OS=Caenorhabditis elegans GN=C53C9.2 PE=3 SV=2 - [YSE2_CAEEL]	7.68	9.32	1	3	3	3	397	44.8	4.65
Q23368	Yeast SEC homolog OS=Caenorhabditis elegans GN=sec-24.2 PE=1 SV=3 - [Q23368_CAEEL]	7.65	3.86	1	3	3	3	984	109.3	7.81
Q09582	Probable methionine synthase OS=Caenorhabditis elegans GN=metr-1 PE=3 SV=1 - [METH_CAEEL]	7.57	3.36	1	4	4	4	1249	138.8	5.12
Q21436	Nuclear Pore complex Protein OS=Caenorhabditis elegans GN=npp-3 PE=1 SV=1 - [Q21436_CAEEL]	7.56	1.95	1	3	3	3	1696	191.2	6.24
P34519	Putative tricarboxylate transport protein, mitochondrial OS=Caenorhabditis elegans GN=K11H3.3 PE=3 SV=1 - [TXTP_CAEEL]	7.55	8.65	2	3	3	3	312	34.2	9.33
Q20049	Isocitrate dehydrogenase [NAD] subunit, mitochondrial OS=Caenorhabditis elegans GN=idhg-1 PE=1 SV=2 - [Q20049_CAEEL	7.50	6.82	1	2	3	3	396	43.6	7.68
P37806	Protein unc-87 OS=Caenorhabditis elegans GN=unc-87 PE=1 SV=3 - [UNC87_CAEEL]	7.43	5.49	1	3	3	3	565	62.7	9.07
Q18359	ADH dehydrogenase [ubiquinone] 1 alpha subcomplex subunit 5 OS=Caenorhabditis elegans GN=C33A12.1 PE=3 SV=1 - [NDU	7.43	19.33	1	3	3	3	150	17.3	9.33
Q10578	DNA-directed RNA polymerase II subunit RPB2 OS=Caenorhabditis elegans GN=rpb-2 PE=2 SV=2 - [RPB2_CAEEL]	7.41	3.52	1	3	3	3	1193	134.8	6.84
Q9U329	Uncharacterized protein OS=Caenorhabditis elegans GN=CELE_W09C5.8 PE=1 SV=1 - [Q9U329_CAEEL]	7.41	17.14	1	2	2	3	175	20.2	8.32
Q93573	Translationally-controlled tumor protein homolog OS=Caenorhabditis elegans GN=tct-1 PE=1 SV=1 - [TCTP_CAEEL]	7.41	24.31	1	2	2	2	181	20.5	4.91
G5EEE5	Elongation of very long chain fatty acids protein OS=Caenorhabditis elegans GN=elo-1 PE=1 SV=1 - [G5EEE5_CAEEL]	7.31	12.50	2	4	4	4	288	33.5	9.69
O44183	Uncharacterized protein OS=Caenorhabditis elegans GN=CELE_ZC416.6 PE=1 SV=2 - [O44183_CAEEL]	7.28	6.88	2	3	3	3	625	71.3	6.23
P24894	Cytochrome c oxidase subunit 2 OS=Caenorhabditis elegans GN=ctc-2 PE=3 SV=2 - [COX2_CAEEL]	7.28	12.12	1	3	3	3	231	26.5	4.96
P34647	DNA replication licensing factor mcm-6 OS=Caenorhabditis elegans GN=mcm-6 PE=1 SV=1 - [MCM6_CAEEL]	7.27	4.94	1	4	4	4	810	91.1	5.33
G5EGN2	Delta(9)-fatty-acid desaturase fat-6 OS=Caenorhabditis elegans GN=fat-6 PE=2 SV=1 - [FAT6_CAEEL]	7.20	5.90	1	2	2	3	339	39.0	8.47
P16356	DNA-directed RNA polymerase II subunit RPB1 OS=Caenorhabditis elegans GN=ama-1 PE=1 SV=3 - [RPB1_CAEEL]	7.13	3.23	1	5	5	5	1856	204.4	6.74
Q7JMQ6	AQuaPorin or aquaglyceroporin related OS=Caenorhabditis elegans GN=aqp-2 PE=1 SV=1 - [Q7JMQ6_CAEEL]	7.04	5.61	2	1	1	2	285	30.8	8.66
Q93425	Putative 28S ribosomal protein S5, mitochondrial OS=Caenorhabditis elegans GN=mrps-5 PE=3 SV=3 - [RT05_CAEEL]	7.03	7.57	1	3	3	3	436	49.6	9.92
O76371	Proteasome Regulatory Particle, ATPase-like OS=Caenorhabditis elegans GN=rpt-5 PE=1 SV=1 - [O76371_CAEEL]	6.92	6.74	1	3	3	3	430	48.1	5.29
Q18486	Atypical kinase coq-8, mitochondrial OS=Caenorhabditis elegans GN=coq-8 PE=3 SV=2 - [COQ8_CAEEL]	6.92	5.70	1	3	3	3	755	83.6	7.28
O61792	Proteasome Regulatory Particle, Non-ATPase-like OS=Caenorhabditis elegans GN=rpn-8 PE=1 SV=1 - [O61792_CAEEL]	6.91	9.94	1	3	3	3	362	40.7	6.48
G5ECS9	Carnitine Palmitoyl Transferase OS=Caenorhabditis elegans GN=cpt-2 PE=1 SV=2 - [G5ECS9_CAEEL]	6.86	7.89	2	4	4	4	646	72.5	7.91
P90860	Uncharacterized protein OS=Caenorhabditis elegans GN=CELE_F36A2.7 PE=1 SV=1 - [P90860_CAEEL]	6.84	29.34	1	4	4	4	167	19.5	9.63
Q22438	39S ribosomal protein L51, mitochondrial OS=Caenorhabditis elegans GN=mrpl-51 PE=3 SV=1 - [RM51_CAEEL]	6.82	12.56	1	2	2	3	199	23.6	9.55
Q19901	Heavy chain, Unconventional Myosin OS=Caenorhabditis elegans GN=hum-1 PE=1 SV=1 - [Q19901_CAEEL]	6.80	3.27	1	3	3	3	1100	124.8	8.95
Q93540	Uncharacterized protein OS=Caenorhabditis elegans GN=CELE_F20D1.9 PE=1 SV=2 - [Q93540_CAEEL]	6.74	9.58	2	3	3	3	313	33.5	9.61
G5EBH7	CALUmenin (Calcium-binding protein) homolog OS=Caenorhabditis elegans GN=calu-1 PE=1 SV=1 - [G5EBH7_CAEEL]	6.72	13.38	2	3	3	3	314	36.1	4.82
O17157	Uncharacterized protein OS=Caenorhabditis elegans GN=C24H12.4 PE=1 SV=2 - [O17157_CAEEL]	6.69	4.89	3	3	3	3	634	71.9	9.28
G5EDS2	POD-1 OS=Caenorhabditis elegans GN=pod-1 PE=1 SV=1 - [G5EDS2_CAEEL]	6.67	3.12	4	3	3	3	1057	115.6	7.01

G5EDY8	Dynamin-Related Protein OS=Caenorhabditis elegans GN=drp-1 PE=1 SV=1 - [G5EDY8_CAEEL]	6.66	4.40	2	3	3	3	705	79.2	6.64
Q20203	Calsequestrin OS=Caenorhabditis elegans GN=csq-1 PE=1 SV=1 - [Q20203_CAEEL]	6.65	6.71	1	3	3	3	417	48.3	4.35
Q9UAQ6	RAB family OS=Caenorhabditis elegans GN=rab-1 PE=1 SV=1 - [Q9UAQ6_CAEEL]	6.62	15.61	1	3	3	3	205	22.5	5.71
G5EFY6	Yeast SEC homolog OS=Caenorhabditis elegans GN=sec-31 PE=1 SV=1 - [G5EFY6_CAEEL]	6.59	4.16	1	3	3	3	1083	119.5	6.35
V6CK48	Fatty Acid CoA Synthetase family OS=Caenorhabditis elegans GN=acs-13 PE=1 SV=1 - [V6CK48_CAEEL]	6.56	5.33	3	3	3	3	676	74.5	6.84
Q09374	Uncharacterized protein ZK177.8 OS=Caenorhabditis elegans GN=ZK177.8 PE=4 SV=2 - [YS48_CAEEL]	6.54	7.50	1	3	3	3	587	66.5	6.68
Q9GZH2	RuvB-like 2 OS=Caenorhabditis elegans GN=ruvb-2 PE=1 SV=1 - [RUVB2_CAEEL]	6.52	8.71	1	3	3	3	448	49.3	5.81
Q93831	Uncharacterized protein OS=Caenorhabditis elegans GN=CELE_F59C6.5 PE=1 SV=1 - [Q93831_CAEEL]	6.46	8.85	1	2	2	2	260	31.2	6.57
O01757	RNA cytidine acetyltransferase OS=Caenorhabditis elegans GN=nath-10 PE=3 SV=1 - [NAT10_CAEEL]	6.45	3.93	1	4	4	4	1043	116.9	8.22
Q9N3F7	Uncharacterized protein OS=Caenorhabditis elegans GN=CELE_Y53G8AR.9 PE=1 SV=1 - [Q9N3F7_CAEEL]	6.30	11.75	1	3	3	3	315	35.1	7.71
Q94360	DH dehydrogenase [ubiquinone] iron-sulfur protein 7, mitochondrial OS=Caenorhabditis elegans GN=nduf-7 PE=3 SV=1 - [NDU	6.28	12.56	1	2	2	2	199	21.9	9.60
A0A1N7SYP7	Eukaryotic translation initiation factor 3 subunit L OS=Caenorhabditis elegans GN=eif-3.L PE=1 SV=1 - [A0A1N7SYP7_CAEEL]	6.27	9.96	4	4	4	4	492	57.2	5.57
Q95XS1	TRanslocon-Associated Protein OS=Caenorhabditis elegans GN=trap-3 PE=1 SV=1 - [Q95XS1_CAEEL]	6.23	16.85	1	3	3	3	178	19.7	8.68
K7ZUH9	Histone H3 OS=Caenorhabditis elegans GN=his-40 PE=1 SV=1 - [K7ZUH9_CAEEL]	6.19	16.91	6	3	3	3	136	15.3	11.37
A0A061AD41	Proline dehydrogenase OS=Caenorhabditis elegans GN=B0513.5 PE=1 SV=1 - [A0A061AD41_CAEEL]	6.18	5.44	2	2	2	2	515	58.5	8.19
Q95ZT1	Fourteen-Three-Three family OS=Caenorhabditis elegans GN=ftt-2 PE=1 SV=2 - [Q95ZT1_CAEEL]	6.18	14.65	3	1	3	3	198	22.6	7.09
G5EFS5	Uncharacterized protein OS=Caenorhabditis elegans GN=CELE_F45D11.15 PE=4 SV=1 - [G5EFS5_CAEEL]	6.14	7.39	2	4	4	4	501	58.5	5.66
Q19853	Uncharacterized protein OS=Caenorhabditis elegans GN=CELE_F28B4.3 PE=1 SV=1 - [Q19853_CAEEL]	6.06	1.79	1	2	3	3	2229	244.7	5.02
W6RRU8	NOT-Like (Yeast CCR4/NOT complex component) OS=Caenorhabditis elegans GN=ntl-4 PE=1 SV=1 - [W6RRU8_CAEEL]	6.00	5.29	4	4	4	4	794	90.0	5.94
A0A1X7RC97	Uncharacterized protein OS=Caenorhabditis elegans GN=CELE_T21F4.1 PE=4 SV=1 - [A0A1X7RC97_CAEEL]	5.97	9.79	1	3	3	3	327	35.9	5.96
Q93896	MonoCarboxylate Transporter family OS=Caenorhabditis elegans GN=mct-3 PE=1 SV=1 - [Q93896_CAEEL]	5.93	7.21	1	3	3	3	596	63.8	5.88
Q9N5E4	Uncharacterized protein OS=Caenorhabditis elegans GN=CELE_T02H6.11 PE=1 SV=1 - [Q9N5E4_CAEEL]	5.92	16.92	1	2	2	3	130	15.1	8.38
Q20779	obable cytochrome c oxidase subunit 6A, mitochondrial OS=Caenorhabditis elegans GN=tag-174 PE=3 SV=1 - [COX6A_CAEE	5.91	22.66	1	2	2	2	128	14.7	9.54
O17915	GTP-binding nuclear protein ran-1 OS=Caenorhabditis elegans GN=ran-1 PE=1 SV=1 - [RAN_CAEEL]	5.87	14.88	1	3	3	3	215	24.2	7.44
Q18801	GDP-mannose 4,6 dehydratase 1 OS=Caenorhabditis elegans GN=bre-1 PE=1 SV=3 - [GMD1_CAEEL]	5.85	6.27	1	2	2	2	399	44.5	6.42
O18000	Patterned Expression Site OS=Caenorhabditis elegans GN=pes-9 PE=1 SV=1 - [O18000_CAEEL]	5.84	5.07	1	2	2	2	473	52.5	5.38
Q22169	Translocon-associated protein subunit beta OS=Caenorhabditis elegans GN=trap-2 PE=1 SV=1 - [SSRB_CAEEL]	5.82	10.64	1	2	2	2	188	21.5	9.09
Q20641	Non-muscle MYosin OS=Caenorhabditis elegans GN=nmy-1 PE=1 SV=2 - [Q20641_CAEEL]	5.80	1.38	1	2	2	2	1963	229.2	5.72
Q09EE7	Uncharacterized protein OS=Caenorhabditis elegans GN=nsf-1 PE=1 SV=1 - [Q09EE7_CAEEL]	5.78	3.83	2	3	3	3	758	83.8	7.08
Q93413	Dicer Related Helicase OS=Caenorhabditis elegans GN=drh-3 PE=1 SV=3 - [Q93413_CAEEL]	5.77	2.41	1	2	2	2	1119	129.0	7.14
Q6BEV4	Ubiquitin Fusion Degradation (Yeast UFD homolog) OS=Caenorhabditis elegans GN=ufd-2 PE=1 SV=1 - [Q6BEV4_CAEEL]	5.76	2.55	3	2	2	2	979	113.1	5.57
Q9XU97	Uncharacterized protein OS=Caenorhabditis elegans GN=CELE_F44E5.1 PE=1 SV=1 - [Q9XU97_CAEEL]	5.73	29.49	1	2	2	2	78	8.9	10.27
Q18853	CYtochrome C OS=Caenorhabditis elegans GN=cyc-1 PE=1 SV=1 - [Q18853_CAEEL]	5.72	9.12	1	2	2	2	285	30.9	8.57
Q93235	Sodium/potassium-transporting ATPase subunit beta-1 OS=Caenorhabditis elegans GN=nkb-1 PE=1 SV=1 - [AT1B1_CAEEL]	5.71	14.69	1	3	4	4	320	36.6	7.58
Q9U3Q6	UDP-glucuronosyltransferase OS=Caenorhabditis elegans GN=ugt-22 PE=1 SV=1 - [Q9U3Q6_CAEEL]	5.70	7.30	1	3	3	3	534	60.2	8.00
O62107	UDP-GALactose 4-Epimerase OS=Caenorhabditis elegans GN=gale-1 PE=1 SV=1 - [O62107_CAEEL]	5.68	9.22	2	2	2	2	347	37.7	6.24
Q2EEM5	USO1 (Yeast transport protein) homolog OS=Caenorhabditis elegans GN=uso-1 PE=1 SV=1 - [Q2EEM5_CAEEL]	5.68	2.97	2	2	2	2	875	99.3	4.97
Q8T3D2	Uncharacterized protein OS=Caenorhabditis elegans GN=CELE_T08G11.1 PE=1 SV=1 - [Q8T3D2_CAEEL]	5.63	1.57	2	4	4	4	3185	356.3	6.34
Q23044	Uncharacterized protein OS=Caenorhabditis elegans GN=CELE_T22B7.7 PE=1 SV=1 - [Q23044_CAEEL]	5.62	8.40	1	3	3	3	393	44.4	8.46
O44738	Uncharacterized protein OS=Caenorhabditis elegans GN=CELE_F57B10.5 PE=1 SV=1 - [O44738_CAEEL]	5.54	6.40	1	1	1	2	203	23.2	5.82
O02158	Uncharacterized protein OS=Caenorhabditis elegans GN=CELE_T09B4.8 PE=1 SV=3 - [O02158_CAEEL]	5.53	5.18	1	2	2	2	444	48.6	8.32
Q17339	Female germline-specific tumor suppressor gld-1 OS=Caenorhabditis elegans GN=gld-1 PE=1 SV=1 - [GLD1_CAEEL]	5.46	6.05	1	3	3	3	463	50.6	8.21
P09588	Histone H2A OS=Caenorhabditis elegans GN=his-3 PE=1 SV=2 - [H2A_CAEEL]	5.42	12.60	4	1	2	2	127	13.4	10.70
Q965S8	Eukaryotic translation initiation factor 3 subunit I OS=Caenorhabditis elegans GN=eif-3.I PE=3 SV=2 - [EIF3I_CAEEL]	5.41	5.81	1	2	2	2	327	36.8	5.54
Q22508	Uncharacterized protein OS=Caenorhabditis elegans GN=tag-18 PE=1 SV=1 - [Q22508_CAEEL]	5.36	10.45	1	2	2	2	201	24.4	9.72
Q09930	GTP-binding protein drn-1 (Fragment) OS=Caenorhabditis elegans GN=drn-1 PE=1 SV=3 - [DIRA_CAEEL]	5.28	18.26	1	2	2	2	219	23.8	7.21
P34438	Uncharacterized protein F44E2.8 OS=Caenorhabditis elegans GN=F44E2.8 PE=4 SV=2 - [YL58_CAEEL]	5.27	9.50	1	2	2	2	242	27.2	8.88
G5EEP9	FERM domain (Protein4.1-ezrin-radixin-moesin) family OS=Caenorhabditis elegans GN=frm-1 PE=1 SV=1 - [G5EEP9_CAEEL]	5.20	4.99	4	3	3	3	4530	512.2	6.43
Q22814	Glutathione S-Transferase OS=Caenorhabditis elegans GN=gst-13 PE=1 SV=1 - [Q22814_CAEEL]	5.19	16.35	1	3	3	3	208	24.3	5.52
P91857	RAB family OS=Caenorhabditis elegans GN=rab-5 PE=1 SV=1 - [P91857_CAEEL]	5.18	12.02	1	2	2	2	208	22.8	7.77
O16298	Uncharacterized protein OS=Caenorhabditis elegans GN=hpo-18 PE=1 SV=1 - [O16298_CAEEL]	5.18	33.33	2	2	2	2	54	6.0	10.17
Q22308	DEAD boX helicase homolog OS=Caenorhabditis elegans GN=ddx-19 PE=1 SV=2 - [Q22308_CAEEL]	5.18	3.91	5	2	2	2	1022	108.2	8.57
P90992	MItochondrial Solute Carrier OS=Caenorhabditis elegans GN=misc-1 PE=1 SV=2 - [P90992_CAEEL]	5.15	7.84	1	2	2	2	306	33.3	9.76
Q9N456	GLutaRedoXin OS=Caenorhabditis elegans GN=glrx-10 PE=1 SV=1 - [Q9N456_CAEEL]	5.15	22.86	1	2	2	2	105	11.3	8.28
P15796	Histone H1.2 OS=Caenorhabditis elegans GN=hil-2 PE=1 SV=3 - [H12_CAEEL]	5.14	6.81	1	1	1	2	191	19.8	10.81
P34388	Probable 40S ribosomal protein S9, mitochondrial OS=Caenorhabditis elegans GN=mrps-9 PE=3 SV=2 - [RT09_CAEEL]	5.13	10.46	1	3	3	3	392	43.7	9.58
Q9BL86	Mitochondrial Ribosomal Protein, Large OS=Caenorhabditis elegans GN=mrpl-38 PE=1 SV=1 - [Q9BL86_CAEEL]	5.08	7.75	1	3	3	3	413	48.0	7.52

Q17430	SART-3/p110 homolog OS=Caenorhabditis elegans GN=sart-3 PE=1 SV=1 - [Q17430_CAEEL]	5.05	4.07	1	2	2	2	836	95.4	6.86
O02155	Uncharacterized protein OS=Caenorhabditis elegans GN=CELE_T09B4.5 PE=1 SV=1 - [O02155_CAEEL]	5.02	12.23	1	3	3	3	327	36.4	11.49
M1ZJY1	SR Protein Kinase OS=Caenorhabditis elegans GN=spk-1 PE=1 SV=1 - [M1ZJY1_CAEEL]	5.02	4.20	2	2	2	2	619	70.0	5.67
Q9TXQ1	Tankyrase-like protein OS=Caenorhabditis elegans GN=tank-1 PE=2 SV=1 - [TNKS1_CAEEL]	4.99	2.46	1	5	5	5	2276	254.4	8.62
Q95YB2	Acyl CoA DeHydrogenase OS=Caenorhabditis elegans GN=acdh-9 PE=1 SV=1 - [Q95YB2_CAEEL]	4.99	7.82	1	2	2	2	409	45.2	7.09
Q17512	Uncharacterized protein OS=Caenorhabditis elegans GN=B0491.5 PE=1 SV=1 - [Q17512_CAEEL]	4.98	10.09	1	2	2	2	327	36.4	9.13
Q23424	Transmembrane GTPase fzo-1 OS=Caenorhabditis elegans GN=fzo-1 PE=3 SV=1 - [FZO1_CAEEL]	4.94	2.33	1	2	2	2	774	87.4	6.51
G8JXY9	Uncharacterized protein OS=Caenorhabditis elegans GN=B0416.5 PE=1 SV=1 - [G8JXY9_CAEEL]	4.93	5.39	2	2	2	2	464	50.4	7.17
O16520	Eukaryotic peptide chain release factor subunit 1 OS=Caenorhabditis elegans GN=erfa-1 PE=3 SV=2 - [ERF1_CAEEL]	4.92	4.11	1	2	2	2	559	62.1	7.37
O61815	RNA helicase OS=Caenorhabditis elegans GN=B0511.6 PE=1 SV=2 - [O61815_CAEEL]	4.91	4.23	1	2	2	2	544	61.2	9.00
Q21966	ASpartyl Protease OS=Caenorhabditis elegans GN=asp-4 PE=1 SV=1 - [Q21966_CAEEL]	4.89	6.76	1	2	2	2	444	49.2	6.55
Q9N384	Galectin OS=Caenorhabditis elegans GN=lec-6 PE=1 SV=1 - [Q9N384_CAEEL]	4.88	22.60	1	2	2	2	146	16.0	6.68
O01925	Lysophospholipid acyltransferase 5 OS=Caenorhabditis elegans GN=mboa-6 PE=1 SV=2 - [MBOA5_CAEEL]	4.88	5.29	1	2	2	2	473	54.1	8.65
Q5WRM0	Uncharacterized protein OS=Caenorhabditis elegans GN=CELE_K06G5.1 PE=1 SV=1 - [Q5WRM0_CAEEL]	4.87	4.70	2	2	2	2	489	52.7	4.86
Q93576	Nucleoside diphosphate kinase OS=Caenorhabditis elegans GN=ndk-1 PE=1 SV=1 - [Q93576_CAEEL]	4.85	16.99	1	2	2	2	153	17.1	7.50
Q20950	Uncharacterized protein OS=Caenorhabditis elegans GN=CELE_F57F5.1 PE=1 SV=2 - [Q20950_CAEEL]	4.85	7.98	1	2	2	2	351	38.3	5.90
Q9GZH3	Inosine-5'-monophosphate dehydrogenase OS=Caenorhabditis elegans GN=T22D1.3 PE=3 SV=2 - [IMDH_CAEEL]	4.85	5.43	3	2	2	2	534	58.1	7.40
H2L0E5	MonoCarboxylate Transporter family OS=Caenorhabditis elegans GN=mct-4 PE=1 SV=1 - [H2L0E5_CAEEL]	4.85	4.14	3	2	2	2	677	73.7	5.20
Q9U1Y6	DeHydrogenases, Short chain OS=Caenorhabditis elegans GN=dhs-24 PE=1 SV=3 - [Q9U1Y6_CAEEL]	4.84	8.33	1	2	2	2	384	43.1	8.54
P91350	Nuclear Pore complex Protein OS=Caenorhabditis elegans GN=npp-6 PE=1 SV=1 - [P91350_CAEEL]	4.81	1.22	2	2	2	2	1562	176.0	5.55
Q21776	Mitotic checkpoint serine/threonine-protein kinase bub-1 OS=Caenorhabditis elegans GN=bub-1 PE=1 SV=1 - [BUB1_CAEEL]	4.81	2.23	1	2	2	2	987	112.0	5.66
H2KYN3	Acyl CoA DeHydrogenase OS=Caenorhabditis elegans GN=acdh-13 PE=1 SV=1 - [H2KYN3_CAEEL]	4.80	3.24	1	2	2	2	617	67.9	6.38
Q17698	Uncharacterized protein OS=Caenorhabditis elegans GN=C06A8.3 PE=1 SV=1 - [Q17698_CAEEL]	4.76	13.25	1	1	1	2	151	15.9	8.13
G8JYE5	Uncharacterized protein OS=Caenorhabditis elegans GN=CELE_ZK546.14 PE=1 SV=1 - [G8JYE5_CAEEL]	4.76	8.15	2	3	3	3	454	52.2	9.57
Q9U2Q9	Glycogen synthase kinase-3 OS=Caenorhabditis elegans GN=gsk-3 PE=1 SV=1 - [GSK3_CAEEL]	4.75	6.08	1	2	2	2	362	40.9	8.82
H2L0I7	Heavy chain, Unconventional Myosin OS=Caenorhabditis elegans GN=hum-8 PE=1 SV=2 - [H2L0I7_CAEEL]	4.74	1.98	3	2	2	2	1265	144.9	8.88
G5EBZ4	Protein let-418 OS=Caenorhabditis elegans GN=let-418 PE=1 SV=1 - [LE418_CAEEL]	4.74	1.53	1	2	2	2	1829	209.0	5.20
Q95ZS5	Uncharacterized protein OS=Caenorhabditis elegans GN=CELE_F56A8.3 PE=1 SV=1 - [Q95ZS5_CAEEL]	4.74	6.53	2	2	2	2	444	49.4	9.64
Q8IG19	ALdehyde deHydrogenase OS=Caenorhabditis elegans GN=alh-1 PE=1 SV=1 - [Q8IG19_CAEEL]	4.73	10.66	2	4	4	4	422	45.6	6.39
Q09444	Probable ubiquitin carboxyl-terminal hydrolase ubh-4 OS=Caenorhabditis elegans GN=ubh-4 PE=3 SV=2 - [UBH4_CAEEL]	4.71	7.79	1	2	2	2	321	37.1	4.98
O16517	ATP synthase subunit OS=Caenorhabditis elegans GN=atp-4 PE=1 SV=1 - [O16517_CAEEL]	4.67	17.05	1	2	2	2	129	13.9	9.50
G5EEB4	SZY-20B OS=Caenorhabditis elegans GN=szy-20 PE=1 SV=1 - [G5EEB4_CAEEL]	4.65	12.91	7	3	3	4	457	52.3	8.68
Q966C7	Transaldolase OS=Caenorhabditis elegans GN=tald-1 PE=1 SV=1 - [Q966C7_CAEEL]	4.64	10.97	2	2	2	2	319	35.3	6.44
Q19722	Asparagine--tRNA ligase, cytoplasmic OS=Caenorhabditis elegans GN=nrs-1 PE=3 SV=1 - [SYNC_CAEEL]	4.61	3.49	1	2	2	2	545	61.1	6.33
P34559	Probable enoyl-CoA hydratase, mitochondrial OS=Caenorhabditis elegans GN=ech-6 PE=3 SV=1 - [ECHM_CAEEL]	4.60	10.76	1	2	2	2	288	31.2	8.38
O45451	Glutathione S-Transferase OS=Caenorhabditis elegans GN=gst-38 PE=1 SV=1 - [O45451_CAEEL]	4.60	11.00	1	2	2	2	209	23.7	6.23
A5Z2U5	Uncharacterized protein OS=Caenorhabditis elegans GN=C27A7.5 PE=1 SV=1 - [A5Z2U5_CAEEL]	4.59	3.40	4	2	2	2	647	71.5	5.68
G5ECK7	Dynein Light Intermediate chain OS=Caenorhabditis elegans GN=dli-1 PE=1 SV=1 - [G5ECK7_CAEEL]	4.58	6.58	2	2	2	2	441	48.4	5.99
Q9U3C8	Defective in cullin neddylation protein 1 OS=Caenorhabditis elegans GN=dcn-1 PE=1 SV=2 - [DCN1_CAEEL]	4.56	13.90	1	2	2	3	295	34.1	5.30
Q8WQB3	AdaPtin, Gamma chain (Clathrin associated complex) OS=Caenorhabditis elegans GN=apg-1 PE=1 SV=1 - [Q8WQB3_CAEEL	4.56	4.22	1	3	3	3	829	91.6	6.33
P34563	Eukaryotic translation initiation factor 5A-1 OS=Caenorhabditis elegans GN=iff-1 PE=1 SV=1 - [IF5A1_CAEEL]	4.56	16.15	2	2	2	2	161	17.9	5.30
G5EEW3	Major Sperm Protein OS=Caenorhabditis elegans GN=msp-74 PE=4 SV=1 - [G5EEW3_CAEEL]	4.55	35.06	17	2	2	2	77	8.7	5.11
P39055	Dynamin OS=Caenorhabditis elegans GN=dyn-1 PE=1 SV=3 - [DYN1_CAEEL]	4.51	3.01	1	2	2	2	830	93.3	7.42
Q18040	Probable ornithine aminotransferase, mitochondrial OS=Caenorhabditis elegans GN=oatr-1 PE=3 SV=3 - [OAT_CAEEL]	4.49	6.64	1	2	2	2	422	46.4	8.47
Q17994	Aspartate aminotransferase OS=Caenorhabditis elegans GN=got-2.2 PE=1 SV=1 - [Q17994_CAEEL]	4.47	7.25	2	2	2	2	414	45.6	9.16
O45148	DihydroLipoamide S-SuccinylTransferase OS=Caenorhabditis elegans GN=dlst-1 PE=1 SV=2 - [O45148_CAEEL]	4.46	7.13	1	2	2	2	463	49.8	9.09
Q9N4A7	Protein SEC13 homolog OS=Caenorhabditis elegans GN=npp-20 PE=3 SV=1 - [SEC13_CAEEL]	4.45	11.50	1	3	3	3	313	34.7	7.02
Q18476	Uncharacterized protein OS=Caenorhabditis elegans GN=C35A5.8 PE=1 SV=3 - [Q18476_CAEEL]	4.43	3.28	1	3	3	3	1096	125.0	5.49
Q21351	tpase-activating protein SH3 (Three) domain-Binding Protein OS=Caenorhabditis elegans GN=gtbp-1 PE=1 SV=2 - [Q21351_C	4.42	5.92	1	2	2	2	473	52.4	9.52
Q27464	Glucose-6-phosphate 1-dehydrogenase OS=Caenorhabditis elegans GN=gspd-1 PE=3 SV=1 - [G6PD_CAEEL]	4.39	3.83	1	2	2	2	522	60.2	8.03
G5EDY2	Tryptophanyl(W) Amino-acyl tRNA Synthetase OS=Caenorhabditis elegans GN=wars-1 PE=1 SV=1 - [G5EDY2_CAEEL]	4.37	12.47	1	3	3	3	417	47.2	6.47
Q9XXD4	ve H/ACA ribonucleoprotein complex subunit 2-like protein OS=Caenorhabditis elegans GN=Y48A6B.3 PE=3 SV=1 - [NHP2_C	4.35	12.27	1	2	2	2	163	18.1	6.79
Q17941	Serine/threonine-protein kinase akt-1 OS=Caenorhabditis elegans GN=akt-1 PE=1 SV=2 - [AKT1_CAEEL]	4.32	4.44	1	2	2	2	541	62.2	5.95
Q9XVR8	Putative H/ACA ribonucleoprotein complex subunit 3 OS=Caenorhabditis elegans GN=nola-3 PE=3 SV=2 - [NOP10_CAEEL]	4.28	26.56	1	2	2	2	64	7.5	10.17
Q18994	Eukaryotic Initiation Factor OS=Caenorhabditis elegans GN=eif-2bepsilon PE=1 SV=1 - [Q18994_CAEEL]	4.24	3.00	1	2	2	2	666	75.5	4.97
Q95YD8	socitrate dehydrogenase [NAD] subunit, mitochondrial OS=Caenorhabditis elegans GN=idhg-2 PE=1 SV=1 - [Q95YD8_CAEEL	4.21	8.31	1	1	2	2	373	40.8	7.39
Q18212	Spliceosome RNA helicase DDX39B homolog OS=Caenorhabditis elegans GN=hel-1 PE=2 SV=1 - [DX39B_CAEEL]	4.18	4.24	1	2	2	2	425	48.5	5.80

Q10664	al specificity mitogen-activated protein kinase kinase mek-2 OS=Caenorhabditis elegans GN=mek-2 PE=1 SV=1 - [MEK2_CAE	4.16	4.65	1	2	2	2	387	42.8	5.73
O16303	DNaJ domain (Prokaryotic heat shock protein) OS=Caenorhabditis elegans GN=dnj-19 PE=1 SV=1 - [O16303_CAEEL]	4.15	2.51	1	1	1	2	439	47.5	7.12
G5EF05	Uncharacterized protein OS=Caenorhabditis elegans GN=CELE_W07E11.1 PE=1 SV=1 - [G5EF05_CAEEL]	4.14	1.29	2	2	2	2	2175	240.7	7.25
A0A0K3AWK1	P-GlycoProtein related OS=Caenorhabditis elegans GN=pgp-2 PE=1 SV=1 - [A0A0K3AWK1_CAEEL]	4.14	1.74	4	2	2	2	1265	140.4	7.62
B2FDA8	Structural maintenance of chromosomes protein 3 OS=Caenorhabditis elegans GN=smc-3 PE=1 SV=1 - [SMC3_CAEEL]	4.14	2.06	1	2	2	2	1261	146.4	7.36
Q9U367	Uncharacterized protein OS=Caenorhabditis elegans GN=CELE_T16G1.9 PE=1 SV=1 - [Q9U367_CAEEL]	4.13	5.50	1	4	4	4	945	105.0	6.73
Q21986	erminal acetyltransferase B complex subunit NAA25 homolog OS=Caenorhabditis elegans GN=cra-1 PE=1 SV=3 - [NAA25_CAE	4.12	3.55	1	3	3	3	958	109.2	6.84
P42168	Casein kinase I isoform alpha OS=Caenorhabditis elegans GN=kin-19 PE=3 SV=1 - [KC1A_CAEEL]	4.09	6.74	3	2	2	2	341	39.0	9.55
O76743	ATP-dependent RNA helicase glh-4 OS=Caenorhabditis elegans GN=glh-4 PE=1 SV=2 - [GLH4_CAEEL]	4.09	2.42	3	1	2	2	1156	120.6	5.44
P90901	Intermediate filament protein ifa-1 OS=Caenorhabditis elegans GN=ifa-1 PE=1 SV=2 - [IFA1_CAEEL]	4.08	2.96	2	1	2	2	575	66.5	6.38
O01833	Histone H1.5 OS=Caenorhabditis elegans GN=hil-5 PE=2 SV=3 - [H15_CAEEL]	4.06	9.78	1	2	2	2	225	23.4	10.81
Q9GYF1	Troponin I 2 OS=Caenorhabditis elegans GN=unc-27 PE=2 SV=2 - [TNNI2_CAEEL]	4.04	7.44	1	1	1	2	242	27.5	5.53
Q9NAG4	Member of AAA family binding CED-4 OS=Caenorhabditis elegans GN=mac-1 PE=1 SV=2 - [Q9NAG4_CAEEL]	4.02	3.20	1	2	2	2	813	88.9	6.64
Q23256	WD repeat-containing protein wdr-5.3 OS=Caenorhabditis elegans GN=wdr-5.3 PE=3 SV=1 - [WDR53_CAEEL]	3.98	4.19	1	2	2	2	501	54.4	8.72
Q8IA49	Malate dehydrogenase OS=Caenorhabditis elegans GN=mdh-1 PE=1 SV=1 - [Q8IA49_CAEEL]	3.97	10.26	2	2	2	2	273	29.1	9.09
Q02335	TPR repeat-containing protein ZK370.8 OS=Caenorhabditis elegans GN=ZK370.8 PE=4 SV=3 - [YOL8_CAEEL]	3.97	3.51	1	2	2	2	569	64.8	6.44
O61820	Eukaryotic translation initiation factor 3 subunit E OS=Caenorhabditis elegans GN=eif-3.E PE=3 SV=1 - [EIF3E_CAEEL]	3.97	4.40	1	2	2	2	432	50.7	6.15
E2JKY8	DyNactin Complex component OS=Caenorhabditis elegans GN=dnc-1 PE=1 SV=1 - [E2JKY8_CAEEL]	3.97	1.84	5	2	2	2	1088	123.6	5.41
Q7KPW7	Mitochondrial Ribosomal Protein, Large OS=Caenorhabditis elegans GN=mrpl-1 PE=1 SV=1 - [Q7KPW7_CAEEL]	3.96	6.90	1	2	2	2	377	42.7	9.28
Q9TYY8	Ubiquitin carboxyl-terminal hydrolase OS=Caenorhabditis elegans GN=CELE_H34C03.2 PE=1 SV=1 - [Q9TYY8_CAEEL]	3.92	2.78	2	2	2	2	900	102.6	5.76
Q19103	SKI (Yeast SuperKIller) Helicase homolog OS=Caenorhabditis elegans GN=skih-2 PE=1 SV=3 - [Q19103_CAEEL]	3.91	1.66	1	2	2	2	1266	142.3	5.96
G5ECG7	Defecation Suppressor of Clk-1 OS=Caenorhabditis elegans GN=dsc-4 PE=1 SV=1 - [G5ECG7_CAEEL]	3.84	2.69	1	2	2	2	892	101.0	7.02
O16997	Splicing Factor ThreeB (3b) Subunit homolog OS=Caenorhabditis elegans GN=sftb-2 PE=1 SV=1 - [O16997_CAEEL]	3.82	3.16	1	2	2	2	602	68.3	5.44
G5EGA5	Delta(12) fatty acid desaturase fat-2 OS=Caenorhabditis elegans GN=fat-2 PE=2 SV=1 - [FAT2_CAEEL]	3.81	6.12	2	2	2	3	376	43.4	7.21
A0A0K3ARH7	Serine Palmitoyl Transferase famiLy OS=Caenorhabditis elegans GN=sptl-1 PE=1 SV=1 - [A0A0K3ARH7_CAEEL]	3.80	10.92	2	2	2	2	174	19.4	5.58
Q94261	COP9/Signalosome and eIF3 complex-shared subunit 1 OS=Caenorhabditis elegans GN=cif-1 PE=1 SV=1 - [EIF3M_CAEEL]	3.77	3.85	1	2	2	2	390	44.1	5.76
P31161	Superoxide dismutase [Mn] 1, mitochondrial OS=Caenorhabditis elegans GN=sod-2 PE=1 SV=1 - [SODM1_CAEEL]	3.76	9.50	2	2	2	2	221	24.5	7.94
Q19246	DeHydrogenases, Short chain OS=Caenorhabditis elegans GN=dhs-25 PE=1 SV=2 - [Q19246_CAEEL]	3.74	15.32	1	2	2	2	248	25.5	8.98
W6SBK6	TaLiN OS=Caenorhabditis elegans GN=tln-1 PE=1 SV=1 - [W6SBK6_CAEEL]	3.74	1.52	4	2	2	2	1511	161.9	6.10
Q9U2G0	ative U5 small nuclear ribonucleoprotein 200 kDa helicase OS=Caenorhabditis elegans GN=snrp-200 PE=3 SV=1 - [U520_CAE	3.67	1.21	1	3	3	3	2145	243.7	6.21
O61708	Prion-like-(Q/N-rich)-domain-bearing protein OS=Caenorhabditis elegans GN=pqn-59 PE=1 SV=2 - [O61708_CAEEL]	3.65	5.48	1	2	2	3	712	77.1	6.11
Q45EJ8	Lin-5 (Five) Interacting protein OS=Caenorhabditis elegans GN=lfi-1 PE=1 SV=1 - [Q45EJ8_CAEEL]	3.65	1.51	3	2	2	2	2117	242.3	6.06
Q11190	n transfer flavoprotein-ubiquinone oxidoreductase, mitochondrial OS=Caenorhabditis elegans GN=let-721 PE=3 SV=2 - [ETFD_	3.61	4.19	1	2	2	2	597	65.3	6.73
L8E6W9	Uncharacterized protein OS=Caenorhabditis elegans GN=CELE_F55F10.1 PE=1 SV=1 - [L8E6W9_CAEEL]	3.61	0.71	2	3	3	3	4362	491.5	5.11
G5EET8	Protein Up-regulated in Daf-2(Gf) OS=Caenorhabditis elegans GN=pud-1.2 PE=1 SV=1 - [G5EET8_CAEEL]	3.56	7.95	1	1	1	1	151	17.2	6.80
O62246	Coatomer subunit epsilon OS=Caenorhabditis elegans GN=cope-1 PE=3 SV=1 - [COPE_CAEEL]	3.55	4.11	1	1	1	1	292	32.8	5.31
A0A168H9W7	Uncharacterized protein OS=Caenorhabditis elegans GN=lin-40 PE=1 SV=1 - [A0A168H9W7_CAEEL]	3.54	3.95	4	2	2	2	735	81.5	5.95
O62198	Uncharacterized protein OS=Caenorhabditis elegans GN=CELE_F32A11.1 PE=1 SV=1 - [O62198_CAEEL]	3.51	5.64	2	2	2	3	319	35.5	10.01
Q18885	Transcription factor BTF3 homolog OS=Caenorhabditis elegans GN=icd-1 PE=1 SV=1 - [BTF3_CAEEL]	3.51	17.39	1	2	2	3	161	17.5	8.76
P90790	Uncharacterized protein OS=Caenorhabditis elegans GN=CELE_D2030.3 PE=1 SV=1 - [P90790_CAEEL]	3.47	4.72	1	2	2	2	508	56.6	7.44
O62360	DNaJ domain (Prokaryotic heat shock protein) OS=Caenorhabditis elegans GN=dnj-17 PE=1 SV=2 - [O62360_CAEEL]	3.47	2.89	1	1	2	2	485	56.8	5.88
Q5TKA3	Fatty Acid CoA Synthetase family OS=Caenorhabditis elegans GN=acs-1 PE=1 SV=1 - [Q5TKA3_CAEEL]	3.46	2.77	3	1	1	1	578	65.3	6.96
O45552	ACetyl-CoA Acyltransferase 2 homolog OS=Caenorhabditis elegans GN=acaa-2 PE=1 SV=2 - [O45552_CAEEL]	3.39	5.08	1	1	1	1	394	41.3	8.98
B6VQ85	Uncharacterized protein OS=Caenorhabditis elegans GN=C23H4.6 PE=1 SV=1 - [B6VQ85_CAEEL]	3.39	1.47	5	1	2	2	1087	126.3	8.18
Q8ITY2	Phosphoenolypyruvate CarboxyKinase OS=Caenorhabditis elegans GN=pck-1 PE=1 SV=1 - [Q8ITY2_CAEEL]	3.38	2.22	3	1	1	1	541	60.6	6.54
B6GUQ2	Uncharacterized protein OS=Caenorhabditis elegans GN=CELE_Y48A6C.6 PE=4 SV=1 - [B6GUQ2_CAEEL]	3.36	2.85	1	1	1	1	421	48.8	5.06
O18216	RNA-binding protein pno-1 OS=Caenorhabditis elegans GN=pno-1 PE=3 SV=1 - [PNO1_CAEEL]	3.36	5.05	1	1	1	1	277	30.7	8.09
Q11067	Probable protein disulfide-isomerase A6 OS=Caenorhabditis elegans GN=tag-320 PE=3 SV=1 - [PDIA6_CAEEL]	3.33	10.68	1	3	3	3	440	47.7	6.16
Q22993	Phosphoethanolamine MethylTransferase OS=Caenorhabditis elegans GN=pmt-2 PE=1 SV=1 - [Q22993_CAEEL]	3.33	5.95	1	2	2	2	437	49.7	5.92
Q21473	AQuaPorin or aquaglyceroporin related OS=Caenorhabditis elegans GN=aqp-7 PE=1 SV=2 - [Q21473_CAEEL]	3.31	5.15	1	1	1	1	291	31.9	8.31
P90789	dehydrogenase [ubiquinone] 1 beta subcomplex subunit 7 OS=Caenorhabditis elegans GN=D2030.4 PE=3 SV=1 - [NDUB7_C	3.26	13.01	1	1	1	1	123	14.4	8.18
P34696	Heat shock protein Hsp-16.1/Hsp-16.11 OS=Caenorhabditis elegans GN=hsp-16.1 PE=2 SV=1 - [HSP11_CAEEL]	3.23	10.34	1	1	1	1	145	16.2	5.58
O02642	te--CoA ligase [ADP/GDP-forming] subunit alpha, mitochondrial OS=Caenorhabditis elegans GN=sucl-2 PE=1 SV=1 - [O02642_	3.21	3.74	1	1	1	1	321	33.3	9.31
Q9U2K8	ABC transporter, class E OS=Caenorhabditis elegans GN=abce-1 PE=1 SV=1 - [Q9U2K8_CAEEL]	3.21	4.10	1	2	2	2	610	69.0	8.25
K8ESK7	Uncharacterized protein OS=Caenorhabditis elegans GN=CELE_R13H4.2 PE=1 SV=1 - [K8ESK7_CAEEL]	3.19	11.46	3	2	2	2	192	23.8	9.48
P91136	Nucleolar complex protein 3 homolog OS=Caenorhabditis elegans GN=C37H5.5 PE=3 SV=3 - [NOC3L_CAEEL]	3.17	1.54	1	1	1	1	778	88.9	8.81
Q22078	Nuclear Pore complex Protein OS=Caenorhabditis elegans GN=npp-2 PE=1 SV=1 - [Q22078_CAEEL]	3.14	2.34	1	1	1	1	598	68.0	5.26

O44887	Innexin OS=Caenorhabditis elegans GN=inx-13 PE=1 SV=1 - [O44887_CAEEL]	3.12	3.90	1	1	1	1	385	45.1	6.95
Q95QU0	PCI domain-containing protein 2 homolog OS=Caenorhabditis elegans GN=C27F2.10 PE=3 SV=1 - [PCID2_CAEEL]	3.09	2.91	1	1	1	1	413	47.1	6.60
O02086	HAIF transporter (PGP related) OS=Caenorhabditis elegans GN=haf-2 PE=1 SV=2 - [O02086_CAEEL]	3.09	2.20	1	1	1	1	773	86.5	7.17
P35603	AP-2 complex subunit mu OS=Caenorhabditis elegans GN=dpy-23 PE=2 SV=2 - [AP2M_CAEEL]	3.04	3.40	1	1	1	1	441	50.3	9.50
O61793	Uncharacterized protein OS=Caenorhabditis elegans GN=CELE_R12E2.13 PE=1 SV=1 - [O61793_CAEEL]	3.02	6.80	1	1	1	1	206	22.8	6.74
G5EBS7	Choline/EthanolaminePhosphoTransferase OS=Caenorhabditis elegans GN=cept-1 PE=1 SV=1 - [G5EBS7_CAEEL]	3.01	3.94	2	1	1	2	381	42.8	5.80
O17607	RuvB-like 1 OS=Caenorhabditis elegans GN=ruvb-1 PE=1 SV=2 - [RUVB1_CAEEL]	3.01	5.25	1	1	1	1	476	52.5	5.97
P47209	T-complex protein 1 subunit epsilon OS=Caenorhabditis elegans GN=cct-5 PE=2 SV=1 - [TCPE_CAEEL]	2.99	3.69	2	2	2	2	542	59.4	5.59
Q86B39	Protein argonaute OS=Caenorhabditis elegans GN=alg-2 PE=1 SV=1 - [Q86B39_CAEEL]	2.98	2.69	4	1	1	1	891	99.5	8.98
Q23295	Mitochondrial Processing Peptidase Beta OS=Caenorhabditis elegans GN=mppb-1 PE=1 SV=2 - [Q23295_CAEEL]	2.97	2.18	1	1	1	1	458	51.1	5.62
Q20870	DAZ protein 1 OS=Caenorhabditis elegans GN=daz-1 PE=2 SV=3 - [DAZ1_CAEEL]	2.97	2.20	1	1	1	1	499	56.1	9.19
H2L0C0	ndoplasmic Membrane protein Complex (Yeast EMC) homolog OS=Caenorhabditis elegans GN=emc-1 PE=1 SV=1 - [H2L0C0_	2.93	2.64	2	2	2	2	946	104.7	6.52
Q18864	Surfeit locus protein 4 homolog OS=Caenorhabditis elegans GN=sft-4 PE=2 SV=1 - [SURF4_CAEEL]	2.93	3.61	1	1	1	1	277	31.8	8.51
Q95Y71	Probable 39S ribosomal protein L45, mitochondrial OS=Caenorhabditis elegans GN=mrpl-45 PE=3 SV=3 - [RM45_CAEEL]	2.90	3.08	1	1	1	1	357	41.3	9.07
Q18983	Uncharacterized protein OS=Caenorhabditis elegans GN=CELE_D2045.2 PE=1 SV=3 - [Q18983_CAEEL]	2.90	0.88	1	1	1	1	1810	200.7	6.10
Q21732	Uncharacterized protein OS=Caenorhabditis elegans GN=CELE_R04F11.2 PE=1 SV=1 - [Q21732_CAEEL]	2.89	10.28	1	1	1	1	107	12.3	8.94
Q10576	Prolyl 4-hydroxylase subunit alpha-1 OS=Caenorhabditis elegans GN=dpy-18 PE=1 SV=2 - [P4HA1_CAEEL]	2.87	2.86	1	1	1	1	559	63.9	6.23
O61818	Mitochondrial Ribosomal Protein, Small OS=Caenorhabditis elegans GN=mrps-30 PE=1 SV=2 - [O61818_CAEEL]	2.86	2.68	1	1	1	1	523	60.1	6.38
Q9U1V9	DNaJ domain (Prokaryotic heat shock protein) OS=Caenorhabditis elegans GN=dnj-29 PE=1 SV=1 - [Q9U1V9_CAEEL]	2.85	4.65	2	3	3	3	752	86.5	5.22
Q22647	chondrial import inner membrane translocase subunit TIM50 OS=Caenorhabditis elegans GN=scpl-4 PE=3 SV=1 - [TIM50_CA	2.84	7.96	1	2	2	2	452	51.3	7.17
Q8MXI1	Ubiquinol-Cytochrome c oxidoReductase complex OS=Caenorhabditis elegans GN=ucr-11 PE=1 SV=1 - [Q8MXI1_CAEEL]	2.84	17.86	1	1	1	1	56	6.7	9.54
Q19202	Apyrase apy-1 OS=Caenorhabditis elegans GN=apy-1 PE=1 SV=2 - [APY1_CAEEL]	2.83	3.10	1	1	1	1	355	40.3	6.30
Q09522	Probable dimethyladenosine transferase OS=Caenorhabditis elegans GN=E02H1.1 PE=3 SV=2 - [DIM1_CAEEL]	2.82	5.52	1	1	1	1	308	34.6	9.83
Q8I4G9	DAB (Drosophila disabled) homolog OS=Caenorhabditis elegans GN=dab-1 PE=1 SV=3 - [Q8I4G9_CAEEL]	2.82	2.74	4	1	1	1	474	51.4	7.94
O45087	DYnein Chain, light Intermediate OS=Caenorhabditis elegans GN=dyci-1 PE=1 SV=1 - [O45087_CAEEL]	2.80	2.49	1	1	1	1	643	72.0	5.33
O17214	Probable fumarate hydratase, mitochondrial OS=Caenorhabditis elegans GN=fum-1 PE=3 SV=1 - [FUMH_CAEEL]	2.80	2.79	1	1	1	1	501	53.6	7.96
O17586	Proteasome subunit alpha type-6 OS=Caenorhabditis elegans GN=pas-1 PE=1 SV=1 - [PSA6_CAEEL]	2.78	5.28	1	1	1	1	246	27.0	8.09
V6CLW5	Na+/K+ ATPase, Beta subunit OS=Caenorhabditis elegans GN=nkb-3 PE=1 SV=1 - [V6CLW5_CAEEL]	2.76	9.90	2	1	2	2	303	34.2	7.11
Q18813	Galectin OS=Caenorhabditis elegans GN=C53D6.7 PE=1 SV=3 - [Q18813_CAEEL]	2.76	4.82	1	1	1	1	311	35.5	8.69
Q65ZB1	PeriLIpiN homolog OS=Caenorhabditis elegans GN=plin-1 PE=1 SV=1 - [Q65ZB1_CAEEL]	2.76	3.38	3	1	1	1	385	42.5	5.41
P34669	chyl-diphosphooligosaccharide--protein glycosyltransferase subunit 3 OS=Caenorhabditis elegans GN=ZK686.3 PE=3 SV=2 - [(	2.73	2.94	1	1	1	1	340	38.8	9.20
O45444	C-type LECtin OS=Caenorhabditis elegans GN=clec-63 PE=1 SV=1 - [O45444_CAEEL]	2.72	3.16	1	1	1	1	411	44.5	5.02
V6CK98	Suppressor Of Clr OS=Caenorhabditis elegans GN=soc-2 PE=1 SV=1 - [V6CK98_CAEEL]	2.72	2.37	3	1	1	1	549	61.6	6.55
Q9U2L2	Uncharacterized protein OS=Caenorhabditis elegans GN=mlt-3 PE=1 SV=2 - [Q9U2L2_CAEEL]	2.70	3.32	1	1	1	1	392	43.1	6.62
Q9XX18	Mitochondrial Ribosomal Protein, Large OS=Caenorhabditis elegans GN=mrpl-22 PE=1 SV=1 - [Q9XX18_CAEEL]	2.69	3.85	1	1	1	1	260	30.5	9.17
Q19661	Uncharacterized protein OS=Caenorhabditis elegans GN=CELE_F21C10.9 PE=1 SV=3 - [Q19661_CAEEL]	2.68	4.10	1	1	1	1	317	36.4	5.95
Q8MNS3	Uncharacterized protein OS=Caenorhabditis elegans GN=CELE_F42G9.1 PE=1 SV=1 - [Q8MNS3_CAEEL]	2.68	5.33	2	2	2	2	469	51.0	4.34
A0A0K3AWA7	Uncharacterized protein OS=Caenorhabditis elegans GN=C29F7.2 PE=1 SV=1 - [A0A0K3AWA7_CAEEL]	2.68	4.26	2	1	1	1	305	35.1	4.93
P34652	Calnexin OS=Caenorhabditis elegans GN=cnx-1 PE=1 SV=1 - [CALX_CAEEL]	2.67	2.10	1	1	1	1	619	69.2	4.64
Q4W5T6	Uncharacterized protein OS=Caenorhabditis elegans GN=CELE_H20J04.9 PE=1 SV=1 - [Q4W5T6_CAEEL]	2.67	5.60	1	1	1	1	268	30.9	7.91
Q19302	Ribosome assembly factor mrt4 OS=Caenorhabditis elegans GN=CELE_F10E7.5 PE=1 SV=1 - [Q19302_CAEEL]	2.66	7.27	1	1	1	1	220	25.2	9.58
Q09436	Zinc finger protein mex-6 OS=Caenorhabditis elegans GN=mex-6 PE=1 SV=3 - [MEX6_CAEEL]	2.64	5.35	1	2	2	2	467	52.4	8.95
Q9BKQ9	CDGSH Iron Sulfur Domain protein homolog OS=Caenorhabditis elegans GN=cisd-3.2 PE=1 SV=1 - [Q9BKQ9_CAEEL]	2.63	19.87	1	2	2	2	156	16.8	9.48
O01789	Structural maintenance of chromosomes protein 1 OS=Caenorhabditis elegans GN=him-1 PE=1 SV=4 - [SMC1_CAEEL]	2.62	0.79	1	1	1	1	1262	144.1	7.59
P55954	Cytochrome c oxidase subunit 5A, mitochondrial OS=Caenorhabditis elegans GN=cco-2 PE=1 SV=2 - [COX5A_CAEEL]	2.62	13.79	1	2	2	2	174	20.1	6.21
A0A1I6CMB1	Uncharacterized protein OS=Caenorhabditis elegans GN=CELE_W02H5.2 PE=1 SV=1 - [A0A1I6CMB1_CAEEL]	2.62	2.26	1	1	1	1	620	69.8	9.45
P28548	Casein kinase II subunit beta OS=Caenorhabditis elegans GN=kin-10 PE=1 SV=2 - [CSK2B_CAEEL]	2.62	5.13	1	1	1	1	234	26.4	5.16
Q20060	Structural maintenance of chromosomes protein 4 OS=Caenorhabditis elegans GN=smc-4 PE=1 SV=1 - [SMC4_CAEEL]	2.60	1.48	1	2	2	2	1549	176.5	8.00
Q19642	Centromere/kinetochore protein zw10 homolog OS=Caenorhabditis elegans GN=czw-1 PE=1 SV=1 - [ZW10_CAEEL]	2.60	4.76	1	3	3	3	778	87.9	5.87
Q17361	Ubiquitin carboxyl-terminal hydrolase 14 OS=Caenorhabditis elegans GN=usp-14 PE=2 SV=2 - [UBP14_CAEEL]	2.60	4.70	1	2	2	2	489	55.8	5.52
Q9GS00	COP9 signalosome complex subunit 1 OS=Caenorhabditis elegans GN=csn-1 PE=1 SV=1 - [CSN1_CAEEL]	2.59	1.50	1	1	1	1	601	68.4	5.62
G4RZC3	ALdehyde deHydrogenase OS=Caenorhabditis elegans GN=alh-6 PE=1 SV=1 - [G4RZC3_CAEEL]	2.58	3.07	3	1	1	1	423	46.0	6.27
O01812	Fatty acid-binding protein homolog 6 OS=Caenorhabditis elegans GN=lbp-6 PE=1 SV=1 - [FABP6_CAEEL]	2.58	9.63	1	1	1	1	135	15.6	7.40
O17687	P (Human Nuclear Autoantigenic Sperm Protein) homolog OS=Caenorhabditis elegans GN=nasp-2 PE=1 SV=1 - [O17687_CA	2.57	1.72	1	1	1	1	582	62.9	4.25
Q09249	Uncharacterized protein C16C10.3 OS=Caenorhabditis elegans GN=C16C10.3 PE=4 SV=1 - [YQ53_CAEEL]	2.57	0.97	1	1	1	1	1032	117.0	9.03
H2KZX4	Uncharacterized protein OS=Caenorhabditis elegans GN=CELE_T13C2.6 PE=1 SV=1 - [H2KZX4_CAEEL]	2.57	1.21	2	1	1	1	909	101.1	5.20
Q9NAB1	Glutathione S-Transferase OS=Caenorhabditis elegans GN=gst-29 PE=1 SV=1 - [Q9NAB1_CAEEL]	2.57	4.78	1	1	1	1	209	23.6	5.87

A0A131MBC3	Uncharacterized protein OS=Caenorhabditis elegans GN=tag-10 PE=1 SV=1 - [A0A131MBC3_CAEEL]	2.55	4.33	4	1	1	1	439	49.9	5.10
Q8TA83	DnaJ homolog dnj-10 OS=Caenorhabditis elegans GN=dnj-10 PE=3 SV=1 - [DNJ10_CAEEL]	2.54	2.63	1	1	1	1	456	50.1	8.79
Q23449	26S proteasome non-ATPase regulatory subunit 8 OS=Caenorhabditis elegans GN=rpn-12 PE=3 SV=1 - [PSMD8_CAEEL]	2.54	5.20	1	1	1	1	250	28.8	5.83
G5EEZ4	3E324 OS=Caenorhabditis elegans GN=3E324 PE=1 SV=1 - [G5EEZ4_CAEEL]	2.52	3.66	1	1	1	1	383	43.2	4.78
Q19554	ABC transporter, class F OS=Caenorhabditis elegans GN=abcf-1 PE=1 SV=1 - [Q19554_CAEEL]	2.52	3.86	1	2	2	2	622	69.1	6.33
Q9NEM5	Uncharacterized protein OS=Caenorhabditis elegans GN=CELE_Y111B2A.3 PE=1 SV=1 - [Q9NEM5_CAEEL]	2.51	2.09	1	1	1	1	718	82.5	5.08
Q5H9M9	Heat Shock Protein OS=Caenorhabditis elegans GN=hsp-25 PE=1 SV=1 - [Q5H9M9_CAEEL]	2.50	22.22	3	2	2	2	108	12.4	6.30
Q21549	Glutathione synthetase OS=Caenorhabditis elegans GN=gss-1 PE=1 SV=2 - [Q21549_CAEEL]	2.50	4.69	1	2	2	2	490	55.4	6.39
P90786	Piwi-like protein OS=Caenorhabditis elegans GN=prg-1 PE=1 SV=1 - [P90786_CAEEL]	2.48	3.16	1	1	1	4	824	93.8	8.44
Q6AW08	Conserved Oligomeric Golgi (COG) Component OS=Caenorhabditis elegans GN=cogc-3 PE=1 SV=1 - [Q6AW08_CAEEL]	2.47	2.02	1	1	1	1	794	89.6	5.54
Q21432	Zinc metalloproteinase nas-11 OS=Caenorhabditis elegans GN=nas-11 PE=1 SV=2 - [NAS11_CAEEL]	2.47	3.63	1	2	2	2	579	65.0	8.19
Q9U1Z6	ABHydrolase Domain containing homolog OS=Caenorhabditis elegans GN=abhd-3.1 PE=1 SV=1 - [Q9U1Z6_CAEEL]	2.46	3.73	1	1	1	1	375	42.1	6.54
Q9U2X0	Protein arginine N-methyltransferase 1 OS=Caenorhabditis elegans GN=prmt-1 PE=1 SV=1 - [ANM1_CAEEL]	2.46	3.45	1	1	1	1	348	39.7	5.71
P55956	Aspartic protease 3 OS=Caenorhabditis elegans GN=asp-3 PE=1 SV=3 - [ASP3_CAEEL]	2.45	5.03	1	1	1	1	398	43.4	5.88
V6CLE7	HAIF transporter (PGP related) OS=Caenorhabditis elegans GN=haf-4 PE=1 SV=1 - [V6CLE7_CAEEL]	2.45	1.66	2	1	1	1	721	80.6	6.96
Q19371	Yeast SEC homolog OS=Caenorhabditis elegans GN=sec-24.1 PE=1 SV=1 - [Q19371_CAEEL]	2.44	1.24	1	1	1	1	1126	121.0	6.83
Q21525	Uncharacterized protein OS=Caenorhabditis elegans GN=CELE_M05D6.6 PE=1 SV=1 - [Q21525_CAEEL]	2.44	9.32	1	1	1	1	161	18.3	9.74
O62137	Acyl-coenzyme A oxidase OS=Caenorhabditis elegans GN=acox-1.2 PE=1 SV=2 - [O62137_CAEEL]	2.43	3.33	3	2	2	2	661	75.0	7.87
Q7JLB0	Reticulon-like protein OS=Caenorhabditis elegans GN=ret-1 PE=1 SV=1 - [Q7JLB0_CAEEL]	2.41	4.41	7	1	1	1	204	22.6	7.88
Q9N3D9	NADH Ubiquinone oxidoreductase Fe-S protein OS=Caenorhabditis elegans GN=nduf-5 PE=1 SV=1 - [Q9N3D9_CAEEL]	2.40	10.74	1	1	1	1	121	14.0	8.57
Q9XWU2	Protein SUP-1 OS=Caenorhabditis elegans GN=sup-1 PE=1 SV=1 - [SUP1_CAEEL]	2.40	13.59	1	1	1	1	103	11.3	7.50
Q94218	Ufm1-specific protease OS=Caenorhabditis elegans GN=odr-8 PE=1 SV=1 - [UFSP_CAEEL]	2.40	2.21	1	1	1	1	589	66.5	7.43
Q22947	RH (Rhesus) antigen Related OS=Caenorhabditis elegans GN=rhr-1 PE=1 SV=1 - [Q22947_CAEEL]	2.40	2.81	1	1	1	1	463	51.1	6.42
Q9NEQ0	Omega-3 fatty acid desaturase fat-1 OS=Caenorhabditis elegans GN=fat-1 PE=2 SV=1 - [FAT1_CAEEL]	2.38	3.23	1	1	1	1	402	46.5	6.62
A0A0M7RFF6	MonoCarboxylate Transporter family OS=Caenorhabditis elegans GN=mct-2 PE=1 SV=1 - [A0A0M7RFF6_CAEEL]	2.38	3.06	2	1	1	1	392	42.7	8.15
Q9TYK1	ble H/ACA ribonucleoprotein complex subunit 1-like protein OS=Caenorhabditis elegans GN=Y66H1A.4 PE=3 SV=2 - [GAR1_C	2.37	6.56	1	1	1	1	244	24.6	10.93
Q21770	Argonaute protein wago-1 OS=Caenorhabditis elegans GN=wago-1 PE=1 SV=1 - [WAGO1_CAEEL]	2.36	1.27	1	1	1	1	945	105.4	8.46
G5ED29	Ataxin-2 homolog OS=Caenorhabditis elegans GN=atx-2 PE=1 SV=1 - [ATX2_CAEEL]	2.36	1.56	2	1	1	1	959	106.0	6.54
G5EDZ7	CaDmium Responsive OS=Caenorhabditis elegans GN=cdr-4 PE=1 SV=1 - [G5EDZ7_CAEEL]	2.35	6.14	1	1	1	1	277	32.0	8.13
O17805	COLlagen OS=Caenorhabditis elegans GN=col-98 PE=1 SV=1 - [O17805_CAEEL]	2.34	2.95	1	1	1	1	305	29.5	10.01
Q22161	Sideroflexin OS=Caenorhabditis elegans GN=sfxn-1.5 PE=1 SV=1 - [Q22161_CAEEL]	2.33	2.78	1	1	1	1	324	35.5	9.22
G5EBH3	SMAll OS=Caenorhabditis elegans GN=sma-1 PE=1 SV=1 - [G5EBH3_CAEEL]	2.33	0.78	4	2	2	2	3953	458.0	6.34
O44652	CYtochrome P450 family OS=Caenorhabditis elegans GN=cyp-35b3 PE=1 SV=1 - [O44652_CAEEL]	2.33	2.00	1	1	1	1	499	56.6	6.76
Q21154	MICOS complex subunit MIC27 OS=Caenorhabditis elegans GN=moma-1 PE=2 SV=1 - [MOMA1_CAEEL]	2.32	12.94	1	2	2	2	201	22.1	7.74
O76630	ndoplasmic Membrane protein Complex (Yeast EMC) homolog OS=Caenorhabditis elegans GN=emc-2 PE=1 SV=1 - [O76630_	2.32	4.42	1	1	1	1	294	33.3	6.01
G5EGP4	V-type proton ATPase subunit a OS=Caenorhabditis elegans GN=vha-6 PE=1 SV=1 - [G5EGP4_CAEEL]	2.31	2.08	1	1	1	1	865	98.5	6.86
Q2L6U9	Uncharacterized protein OS=Caenorhabditis elegans GN=CELE_R07E3.1 PE=1 SV=1 - [Q2L6U9_CAEEL]	2.30	4.31	2	1	1	1	348	39.3	5.90
O18218	NanOS related OS=Caenorhabditis elegans GN=nos-3 PE=1 SV=2 - [O18218_CAEEL]	2.30	4.50	2	2	2	2	867	95.6	7.56
Q9XX15	ibosomal RNA small subunit methyltransferase nep-1 OS=Caenorhabditis elegans GN=Y39A1A.14 PE=3 SV=1 - [NEP1_CAEE	2.29	11.26	1	2	2	2	231	25.7	8.85
Q23483	Uncharacterized protein OS=Caenorhabditis elegans GN=CELE_ZK418.5 PE=1 SV=2 - [Q23483_CAEEL]	2.28	5.33	1	1	1	1	225	25.0	8.92
O62333	IMportin Beta family OS=Caenorhabditis elegans GN=imb-2 PE=1 SV=1 - [O62333_CAEEL]	2.27	2.86	3	1	1	1	419	46.8	4.74
P34462	V-type proton ATPase subunit D OS=Caenorhabditis elegans GN=vha-14 PE=3 SV=1 - [VATD_CAEEL]	2.26	13.62	1	2	2	2	257	28.8	9.22
Q20898	Translational repressor ifet-1 OS=Caenorhabditis elegans GN=ifet-1 PE=1 SV=2 - [IFET1_CAEEL]	2.26	2.50	1	1	1	1	761	83.9	8.82
P91303	Probable V-type proton ATPase subunit G OS=Caenorhabditis elegans GN=vha-10 PE=3 SV=1 - [VATG_CAEEL]	2.26	12.70	1	1	1	1	126	14.5	9.60
G5EBU8	Uncharacterized protein OS=Caenorhabditis elegans GN=CELE_T12D8.9 PE=1 SV=1 - [G5EBU8_CAEEL]	2.26	2.03	2	1	1	1	1181	137.1	5.90
Q94269	Carboxypeptidase OS=Caenorhabditis elegans GN=CELE_K10C2.1 PE=1 SV=2 - [Q94269_CAEEL]	2.26	1.94	1	3	3	3	2314	256.3	5.82
Q21693	Eukaryotic translation initiation factor 4E-2 OS=Caenorhabditis elegans GN=ife-2 PE=1 SV=1 - [IF4E2_CAEEL]	2.25	7.02	1	1	1	1	228	25.7	6.14
Q5FC71	CDGSH Iron Sulfur Domain protein homolog OS=Caenorhabditis elegans GN=cisd-1 PE=1 SV=1 - [Q5FC71_CAEEL]	2.25	13.59	2	1	1	1	103	11.3	8.65
Q21307	al specificity mitogen-activated protein kinase kinase mek-1 OS=Caenorhabditis elegans GN=mek-1 PE=1 SV=2 - [MEK1_CAE	2.24	3.46	1	1	1	1	347	39.4	6.46
G5EC65	Anion exchange protein OS=Caenorhabditis elegans GN=abts-1 PE=1 SV=1 - [G5EC65_CAEEL]	2.23	1.43	3	1	1	1	1119	124.6	6.54
Q20117	Glutamate--cysteine ligase OS=Caenorhabditis elegans GN=gcs-1 PE=1 SV=2 - [GSH1_CAEEL]	2.23	1.99	1	1	1	1	654	74.3	6.71
Q22135	Uncharacterized protein OS=Caenorhabditis elegans GN=CELE_T04A8.6 PE=1 SV=1 - [Q22135_CAEEL]	2.23	7.17	1	2	2	2	307	33.9	10.76
P52899	uvate dehydrogenase E1 component subunit alpha, mitochondrial OS=Caenorhabditis elegans GN=T05H10.6 PE=3 SV=1 - [O	2.22	3.27	2	1	1	1	397	43.8	7.46
Q9NES9	Uncharacterized protein OS=Caenorhabditis elegans GN=CELE_Y39B6A.3 PE=1 SV=1 - [Q9NES9_CAEEL]	2.22	9.30	2	1	1	1	129	14.2	8.85
Q23125	Mitoferrin OS=Caenorhabditis elegans GN=W02B12.9 PE=3 SV=1 - [MFRN_CAEEL]	2.21	9.29	1	2	2	2	312	34.1	8.29
Q9N3F8	RAL (Ras-related GTPase) homolog OS=Caenorhabditis elegans GN=ral-1 PE=1 SV=1 - [Q9N3F8_CAEEL]	2.21	6.57	2	1	1	1	213	24.0	8.05
P41842	Tetratricopeptide repeat-containing protein trd-1 OS=Caenorhabditis elegans GN=trd-1 PE=2 SV=2 - [TTC27_CAEEL]	2.21	1.69	1	1	1	1	771	87.9	5.59

P34712	Multidrug resistance protein pgp-1 OS=Caenorhabditis elegans GN=pgp-1 PE=1 SV=2 - [PGP1_CAEEL]	2.20	0.76	1	1	1	1	1321	145.0	7.85
O76687	Uncharacterized protein OS=Caenorhabditis elegans GN=CELE_M01G5.3 PE=1 SV=2 - [O76687_CAEEL]	2.20	3.39	1	1	1	1	384	44.4	9.01
O44568	Probable peptide chain release factor 1, mitochondrial OS=Caenorhabditis elegans GN=W03F8.3 PE=3 SV=1 - [RF1M_CAEEL	2.20	1.80	1	1	1	1	389	43.5	6.76
Q23494	Uncharacterized protein OS=Caenorhabditis elegans GN=CELE_ZK430.7 PE=1 SV=1 - [Q23494_CAEEL]	2.20	2.93	1	1	1	1	444	50.8	9.44
Q9BLA0	Uncharacterized protein OS=Caenorhabditis elegans GN=C36E6.1 PE=1 SV=1 - [Q9BLA0_CAEEL]	2.18	2.03	1	1	1	1	641	69.9	7.12
P36573	32 kDa beta-galactoside-binding lectin OS=Caenorhabditis elegans GN=lec-1 PE=1 SV=1 - [LEC1_CAEEL]	2.18	13.26	2	3	3	3	279	31.8	6.60
Q22006	Pre-rRNA-processing protein pro-1 OS=Caenorhabditis elegans GN=pro-1 PE=2 SV=1 - [PRO1_CAEEL]	2.18	2.65	1	1	1	1	529	58.5	6.05
Q18390	Uncharacterized protein OS=Caenorhabditis elegans GN=C33F10.12 PE=3 SV=1 - [Q18390_CAEEL]	2.17	7.40	2	2	2	2	392	42.6	9.39
Q9XU67	RBD (RNA binding domain) protein OS=Caenorhabditis elegans GN=rbd-1 PE=1 SV=1 - [Q9XU67_CAEEL]	2.17	3.21	2	2	2	2	872	98.0	7.50
Q93618	nc finger CCCH domain-containing protein 15 homolog OS=Caenorhabditis elegans GN=F27D4.4 PE=3 SV=3 - [ZC3HF_CAEE	2.17	2.67	1	1	1	1	374	43.3	5.54
P24890	Cytochrome b OS=Caenorhabditis elegans GN=ctb-1 PE=1 SV=2 - [CYB_CAEEL]	2.17	2.70	1	1	1	1	370	42.6	8.12
Q23624	Uncharacterized protein OS=Caenorhabditis elegans GN=CELE_ZK829.7 PE=1 SV=2 - [Q23624_CAEEL]	2.17	2.96	1	1	1	1	372	40.2	5.60
O16785	Paralyzed arrest at two-fold protein 6 OS=Caenorhabditis elegans GN=pat-6 PE=1 SV=1 - [PARV_CAEEL]	2.17	4.00	1	1	1	1	375	43.0	6.43
Q9U2Z1	Yeast SEC homolog OS=Caenorhabditis elegans GN=sec-23 PE=1 SV=2 - [Q9U2Z1_CAEEL]	2.17	1.71	1	1	1	1	821	89.6	7.21
Q20719	ADH dehydrogenase [ubiquinone] flavoprotein 2, mitochondrial OS=Caenorhabditis elegans GN=F53F4.10 PE=3 SV=1 - [NDU	2.16	4.60	1	1	1	1	239	26.2	7.02
P91390	-diphosphooligosaccharide--protein glycosyltransferase subunit 2 OS=Caenorhabditis elegans GN=ostd-1 PE=3 SV=1 - [RPN2	2.16	3.21	1	1	1	1	280	31.1	9.03
H2L0J4	Uncharacterized protein OS=Caenorhabditis elegans GN=CELE_T07D3.9 PE=1 SV=2 - [H2L0J4_CAEEL]	2.16	2.53	2	1	1	1	396	45.2	8.46
Q7Z072	TropoNin T OS=Caenorhabditis elegans GN=tnt-2 PE=1 SV=1 - [Q7Z072_CAEEL]	2.16	3.97	2	1	2	2	428	48.7	4.88
X5M8Y9	Nuclear pore protein OS=Caenorhabditis elegans GN=npp-13 PE=1 SV=1 - [X5M8Y9_CAEEL]	2.15	1.37	2	1	1	1	655	73.3	6.51
Q21080	Uncharacterized protein OS=Caenorhabditis elegans GN=CELE_K01C8.1 PE=1 SV=2 - [Q21080_CAEEL]	2.14	2.40	1	1	1	1	499	53.8	8.09
O45380	lpha,alpha-trehalose-phosphate synthase [UDP-forming] 2 OS=Caenorhabditis elegans GN=tps-2 PE=2 SV=3 - [TPS2_CAEEL	2.14	0.90	1	1	1	1	1229	139.8	6.44
Q20448	Zinc finger putative Transcription Factor family OS=Caenorhabditis elegans GN=ztf-7 PE=1 SV=1 - [Q20448_CAEEL]	2.14	3.75	1	2	2	2	614	71.1	5.27
Q9NF11	Hypoxanthine PhosphoRibosylTransferase homolog OS=Caenorhabditis elegans GN=hprt-1 PE=1 SV=1 - [Q9NF11_CAEEL]	2.13	6.07	1	1	1	1	214	23.8	5.25
O02141	Uncharacterized protein OS=Caenorhabditis elegans GN=C46G7.2 PE=1 SV=1 - [O02141_CAEEL]	2.13	4.40	1	1	1	1	250	30.0	9.67
Q9XWP7	Eukaryotic Initiation Factor OS=Caenorhabditis elegans GN=eif-3.j PE=1 SV=1 - [Q9XWP7_CAEEL]	2.13	4.72	1	1	1	1	212	23.0	4.87
Q23520	Glutathione S-Transferase OS=Caenorhabditis elegans GN=gst-30 PE=1 SV=1 - [Q23520_CAEEL]	2.12	4.23	1	1	1	1	213	24.2	8.53
M1ZJV8	Uncharacterized protein OS=Caenorhabditis elegans GN=CELE_Y69A2AR.16 PE=1 SV=1 - [M1ZJV8_CAEEL]	2.12	1.94	2	1	1	1	929	105.4	4.93
Q7JP52	Heat Shock Protein OS=Caenorhabditis elegans GN=hsp-17 PE=1 SV=1 - [Q7JP52_CAEEL]	2.12	14.19	2	2	2	3	148	17.4	6.39
D9PTP5	Sorting NeXin OS=Caenorhabditis elegans GN=snx-6 PE=1 SV=1 - [D9PTP5_CAEEL]	2.12	3.35	1	1	1	1	478	54.7	5.62
Q19706	Eukaryotic translation initiation factor 3 subunit G OS=Caenorhabditis elegans GN=eif-3.G PE=3 SV=2 - [EIF3G_CAEEL]	2.10	9.16	1	2	2	2	262	29.9	6.34
Q21444	Conserved oligomeric Golgi complex subunit 2 OS=Caenorhabditis elegans GN=cogc-2 PE=2 SV=1 - [COG2_CAEEL]	2.10	2.50	1	1	1	1	681	78.5	5.64
Q8I129	Cell-death-Related Nuclease OS=Caenorhabditis elegans GN=crn-3 PE=1 SV=2 - [Q8I129_CAEEL]	2.09	1.61	2	1	1	1	870	100.2	6.38
G5EET3	CaDmium Responsive OS=Caenorhabditis elegans GN=cdr-6 PE=1 SV=1 - [G5EET3_CAEEL]	2.09	6.14	1	1	1	1	277	31.6	7.88
P48727	Serine/threonine-protein phosphatase PP1-beta OS=Caenorhabditis elegans GN=gsp-2 PE=2 SV=1 - [GLC7B_CAEEL]	2.09	3.00	1	1	1	1	333	37.8	6.76
Q95PZ1	ASH (Klarsicht/ANC-1/Syne Homology) Domain Protein OS=Caenorhabditis elegans GN=kdp-1 PE=1 SV=1 - [Q95PZ1_CAEE	2.09	6.21	1	1	1	1	145	15.5	9.51
V6CJC4	Prion-like-(Q/N-rich)-domain-bearing protein OS=Caenorhabditis elegans GN=pqn-22 PE=1 SV=1 - [V6CJC4_CAEEL]	2.09	2.57	20	1	1	1	506	56.2	8.09
O61856	Acetolactate synthase-like protein OS=Caenorhabditis elegans GN=T26C12.1 PE=3 SV=2 - [ILVBL_CAEEL]	2.08	5.63	1	3	3	3	640	69.2	8.73
G3MU66	ALdehyde deHydrogenase OS=Caenorhabditis elegans GN=alh-7 PE=1 SV=1 - [G3MU66_CAEEL]	2.08	6.83	2	1	1	1	205	22.2	7.65
Q94216	DNaJ domain (Prokaryotic heat shock protein) OS=Caenorhabditis elegans GN=dnj-11 PE=1 SV=1 - [Q94216_CAEEL]	2.07	2.04	1	1	1	1	589	69.4	8.92
O62183	Ref/ALY RNA export adaptor family OS=Caenorhabditis elegans GN=aly-2 PE=1 SV=1 - [O62183_CAEEL]	2.07	3.96	1	1	1	1	227	24.5	10.96
O01532	ASpartyl Protease OS=Caenorhabditis elegans GN=asp-5 PE=1 SV=1 - [O01532_CAEEL]	2.07	4.07	1	1	1	1	393	42.0	6.06
O02109	Uncharacterized protein OS=Caenorhabditis elegans GN=CELE_W08F4.3 PE=1 SV=1 - [O02109_CAEEL]	2.06	8.77	1	1	1	1	228	25.6	9.33
P49197	40S ribosomal protein S21 OS=Caenorhabditis elegans GN=rps-21 PE=1 SV=1 - [RS21_CAEEL]	2.06	9.09	1	1	1	1	88	9.7	7.14
Q9N306	Uncharacterized protein OS=Caenorhabditis elegans GN=CELE_Y61A9LA.10 PE=1 SV=4 - [Q9N306_CAEEL]	2.06	0.95	1	1	1	1	1055	120.4	6.65
Q9U1Q2	Glucose-6-phosphate isomerase OS=Caenorhabditis elegans GN=gpi-1 PE=1 SV=1 - [Q9U1Q2_CAEEL]	2.05	2.72	2	1	1	1	551	61.1	6.37
G5ED95	Tyrosine--tRNA ligase OS=Caenorhabditis elegans GN=yars-1 PE=1 SV=1 - [G5ED95_CAEEL]	2.05	1.25	1	1	1	1	722	80.6	6.02
Q22003	Uncharacterized protein OS=Caenorhabditis elegans GN=CELE_R166.2 PE=1 SV=1 - [Q22003_CAEEL]	2.05	2.91	1	1	1	1	618	71.2	5.77
Q9XVL7	ne zipper, EF-hand, TransMembrane mitochondrial protein) homolog OS=Caenorhabditis elegans GN=letm-1 PE=1 SV=1 - [Q9	2.04	1.95	2	1	1	1	770	87.2	6.44
Q19752	FarNesylTransferase, Beta subunit OS=Caenorhabditis elegans GN=fntb-1 PE=1 SV=1 - [Q19752_CAEEL]	2.04	5.24	1	2	2	2	401	45.1	5.26
P90983	Ribosomal Protein, Small subunit OS=Caenorhabditis elegans GN=rps-29 PE=1 SV=2 - [P90983_CAEEL]	2.03	12.50	1	1	1	1	56	6.5	10.17
Q95QS4	Uncharacterized protein OS=Caenorhabditis elegans GN=C34E10.10 PE=1 SV=1 - [Q95QS4_CAEEL]	2.02	6.47	1	1	1	1	232	26.2	9.76
P34475	Tubulin gamma chain OS=Caenorhabditis elegans GN=tbg-1 PE=2 SV=1 - [TBG_CAEEL]	2.02	2.03	1	1	1	1	444	49.9	6.32
G5EDU3	PhosphoLipase D OS=Caenorhabditis elegans GN=pld-1 PE=1 SV=1 - [G5EDU3_CAEEL]	2.02	1.12	1	1	1	1	1427	163.4	7.50
G5EBV6	PGL-3 OS=Caenorhabditis elegans GN=pgl-3 PE=1 SV=1 - [G5EBV6_CAEEL]	2.01	1.30	1	1	1	1	693	74.8	5.19
Q19989	GOLGi associated coiled-coil protein homolog OS=Caenorhabditis elegans GN=golg-2 PE=1 SV=4 - [Q19989_CAEEL]	2.01	0.75	1	1	1	1	928	105.5	5.19
Q19329	Pre-rRNA-processing protein TSR1 homolog OS=Caenorhabditis elegans GN=tag-151 PE=3 SV=1 - [TSR1_CAEEL]	2.01	2.04	1	1	1	1	785	89.2	5.82
O18180	Mitochondrial Ribosomal Protein, Large OS=Caenorhabditis elegans GN=mrpl-12 PE=1 SV=1 - [O18180_CAEEL]	2.01	6.94	1	1	1	1	173	18.4	8.29

Q02328	Huntington interacting protein related 1 OS=Caenorhabditis elegans GN=hipr-1 PE=3 SV=3 - [SLAP2_CAEEL]	2.01	1.40	1	1	1	1	927	104.4	6.13
O44451	ate dehydrogenase E1 component subunit beta, mitochondrial OS=Caenorhabditis elegans GN=pdhb-1 PE=1 SV=2 - [ODPB_C	2.00	2.27	1	1	1	1	352	38.1	6.01
Q8MXD9	Uncharacterized protein OS=Caenorhabditis elegans GN=CELE_E02D9.1 PE=1 SV=1 - [Q8MXD9_CAEEL]	2.00	4.13	2	1	1	1	315	33.1	8.47
Q9N592	Yeast PRP (Splicing factor) related OS=Caenorhabditis elegans GN=prp-31 PE=1 SV=1 - [Q9N592_CAEEL]	1.99	2.38	1	1	1	1	504	55.6	6.37
O44144	PERMeable eggshell OS=Caenorhabditis elegans GN=perm-4 PE=1 SV=2 - [O44144_CAEEL]	1.99	4.23	1	1	1	1	331	34.9	6.23
O17003	N-alpha-AcetylTransferase C complex subunit OS=Caenorhabditis elegans GN=natc-1 PE=1 SV=2 - [O17003_CAEEL]	1.99	1.13	1	1	1	1	799	91.4	5.47
P91494	Uncharacterized protein OS=Caenorhabditis elegans GN=CELE_T23H2.3 PE=1 SV=3 - [P91494_CAEEL]	1.99	1.10	1	1	1	1	1001	111.9	7.59
O16215	Uncharacterized protein OS=Caenorhabditis elegans GN=CELE_F17A9.5 PE=1 SV=1 - [O16215_CAEEL]	1.99	3.55	1	1	1	1	451	50.1	6.48
O02220	Mitochondrial Ribosomal Protein, Large OS=Caenorhabditis elegans GN=mrpl-34 PE=1 SV=1 - [O02220_CAEEL]	1.97	8.47	1	1	1	1	189	21.4	9.99
O45218	Alkyldihydroxyacetonephosphate synthase OS=Caenorhabditis elegans GN=ads-1 PE=2 SV=1 - [ADAS_CAEEL]	1.97	1.34	1	1	1	1	597	66.5	6.83
Q8I7L2	Uncharacterized protein OS=Caenorhabditis elegans GN=hpo-10 PE=1 SV=1 - [Q8I7L2_CAEEL]	1.96	1.72	2	1	1	1	641	72.1	9.73
P34384	Uncharacterized protein F02A9.4b OS=Caenorhabditis elegans GN=F02A9.4 PE=4 SV=3 - [YLPD_CAEEL]	1.96	3.30	1	1	1	1	394	44.4	6.27
Q93314	Uncharacterized protein OS=Caenorhabditis elegans GN=C31E10.6 PE=1 SV=1 - [Q93314_CAEEL]	1.95	3.55	1	2	2	2	591	68.3	8.41
B5BM32	Nuclear eXport Factor OS=Caenorhabditis elegans GN=nxf-1 PE=1 SV=1 - [B5BM32_CAEEL]	1.95	2.46	2	1	1	1	609	68.5	7.06
Q20375	Serine palmitoyltransferase 2 OS=Caenorhabditis elegans GN=sptl-2 PE=3 SV=1 - [SPTC2_CAEEL]	1.94	1.61	1	1	1	1	558	62.7	7.18
Q27492	DNA-directed RNA polymerase subunit beta OS=Caenorhabditis elegans GN=rpc-2 PE=1 SV=2 - [Q27492_CAEEL]	1.93	1.13	1	1	1	1	1154	129.0	8.72
Q7Z0X2	Uncharacterized protein OS=Caenorhabditis elegans GN=CELE_F32B4.4 PE=1 SV=1 - [Q7Z0X2_CAEEL]	1.93	1.51	3	1	1	1	663	72.1	10.71
P30625	cAMP-dependent protein kinase regulatory subunit OS=Caenorhabditis elegans GN=kin-2 PE=1 SV=3 - [KAPR_CAEEL]	1.93	2.73	1	1	1	1	366	41.4	5.17
Q21925	ProstaGlandin E Synthase homolog OS=Caenorhabditis elegans GN=pges-2 PE=1 SV=1 - [Q21925_CAEEL]	1.93	3.17	1	1	1	1	347	40.3	8.79
Q17574	Putative methionine synthase reductase OS=Caenorhabditis elegans GN=tag-165 PE=3 SV=1 - [MTRR_CAEEL]	1.92	4.55	1	2	2	2	682	76.8	5.76
O45293	Probable N-acetylgalactosaminyltransferase 8 OS=Caenorhabditis elegans GN=gly-8 PE=1 SV=1 - [GALT8_CAEEL]	1.92	2.61	1	1	1	1	421	48.3	6.55
Q21454	Probable 39S ribosomal protein L35, mitochondrial OS=Caenorhabditis elegans GN=mrpl-35 PE=3 SV=2 - [RM35_CAEEL]	1.92	5.06	1	1	1	1	158	18.7	9.77
O01816	YTochrome B OS=Caenorhabditis elegans GN=cytb-5.2 PE=1 SV=1 - [O01816_CAEEL]	1.91	9.22	1	1	1	1	141	15.6	4.70
Q19813	COLlagen OS=Caenorhabditis elegans GN=col-140 PE=1 SV=1 - [Q19813_CAEEL]	1.91	3.11	1	1	1	1	289	28.7	5.72
V6CM00	Uncharacterized protein OS=Caenorhabditis elegans GN=CELE_Y34D9A.7 PE=1 SV=1 - [V6CM00_CAEEL]	1.91	4.01	2	1	1	1	299	34.6	5.59
P91430	Ubiquitin-like modifier-activating enzyme 5 OS=Caenorhabditis elegans GN=uba-5 PE=1 SV=1 - [UBA5_CAEEL]	1.90	2.86	1	1	1	1	419	46.5	5.10
Q19753	Uncharacterized protein F23B12.7 OS=Caenorhabditis elegans GN=F23B12.7 PE=3 SV=1 - [YU0O_CAEEL]	1.90	1.15	1	1	1	1	953	108.4	5.59
O17005	Mitochondrial Ribosomal Protein, Large OS=Caenorhabditis elegans GN=mrpl-4 PE=1 SV=1 - [O17005_CAEEL]	1.90	3.01	1	1	1	1	299	34.3	8.38
Q9BL64	Uncharacterized protein OS=Caenorhabditis elegans GN=CELE_Y54H5A.2 PE=1 SV=2 - [Q9BL64_CAEEL]	1.90	3.47	1	1	1	1	375	41.5	5.03
O16521	NADH-cytochrome b5 reductase OS=Caenorhabditis elegans GN=hpo-19 PE=1 SV=1 - [O16521_CAEEL]	1.89	2.59	1	1	1	1	309	34.7	8.05
G5EFK4	ADP-Ribosylation Factor related OS=Caenorhabditis elegans GN=arf-3 PE=1 SV=1 - [G5EFK4_CAEEL]	1.89	6.11	2	1	1	1	180	20.6	6.95
O61742	26S proteasome non-ATPase regulatory subunit 4 OS=Caenorhabditis elegans GN=rpn-10 PE=2 SV=2 - [PSMD4_CAEEL]	1.89	4.34	1	1	1	1	346	37.3	4.65
P91020	Uncharacterized protein OS=Caenorhabditis elegans GN=C07D8.6 PE=1 SV=1 - [P91020_CAEEL]	1.89	2.52	1	1	1	1	317	35.2	5.66
Q19519	Related to yeast Vacuolar Protein Sorting factor OS=Caenorhabditis elegans GN=vps-36 PE=1 SV=2 - [Q19519_CAEEL]	1.88	3.39	1	1	1	1	383	42.7	6.76
O44156	Proteasome subunit alpha type-1 OS=Caenorhabditis elegans GN=pas-6 PE=1 SV=1 - [PSA1_CAEEL]	1.88	11.92	1	2	2	2	260	28.3	7.02
G5EC24	Tyrosine-protein phosphatase non-receptor type ptp-2 OS=Caenorhabditis elegans GN=ptp-2 PE=1 SV=1 - [PTP2_CAEEL]	1.88	2.25	1	1	1	1	668	76.7	8.18
Q09289	Proteasomal ubiquitin receptor ADRM1 homolog OS=Caenorhabditis elegans GN=C56G2.7 PE=3 SV=2 - [ADRM1_CAEEL]	1.88	5.61	1	2	2	2	374	39.7	4.92
Q18803	Probable ATP synthase subunit g 2, mitochondrial OS=Caenorhabditis elegans GN=asg-2 PE=3 SV=1 - [ATPL2_CAEEL]	1.87	8.40	1	1	1	1	131	14.8	9.73
O02207	OXA mitochondrial inner membrane insertase homolog OS=Caenorhabditis elegans GN=oxa-1 PE=1 SV=2 - [O02207_CAEEL]	1.87	2.19	1	1	1	1	366	40.5	9.80
Q21555	Uncharacterized protein OS=Caenorhabditis elegans GN=CELE_M18.3 PE=1 SV=1 - [Q21555_CAEEL]	1.87	2.74	1	1	1	1	475	53.0	6.05
Q9U2V9	Methionine aminopeptidase 2 OS=Caenorhabditis elegans GN=map-2 PE=1 SV=1 - [Q9U2V9_CAEEL]	1.86	5.41	3	2	2	2	444	49.3	6.43
Q17391	Cullin-3 OS=Caenorhabditis elegans GN=cul-3 PE=1 SV=2 - [CUL3_CAEEL]	1.86	1.54	1	1	1	1	777	90.2	7.58
Q9GYQ7	EXPOrtin (Nuclear export receptor) OS=Caenorhabditis elegans GN=xpo-3 PE=1 SV=3 - [Q9GYQ7_CAEEL]	1.86	1.26	2	1	1	1	950	105.6	5.63
Q22020	Uncharacterized protein OS=Caenorhabditis elegans GN=CELE_R53.5 PE=1 SV=1 - [Q22020_CAEEL]	1.86	4.11	1	1	1	1	219	24.1	8.65
P34601	Uncharacterized protein ZK1098.2 OS=Caenorhabditis elegans GN=ZK1098.2 PE=4 SV=3 - [YO62_CAEEL]	1.85	0.92	1	1	1	1	650	74.2	7.49
O16486	N-alpha-AcetylTransferase C complex subunit OS=Caenorhabditis elegans GN=natc-2 PE=1 SV=1 - [O16486_CAEEL]	1.85	6.47	1	1	1	1	278	31.9	6.39
B6VQ78	P-GlycoProtein related OS=Caenorhabditis elegans GN=pgp-5 PE=1 SV=1 - [B6VQ78_CAEEL]	1.85	1.03	6	1	1	1	1160	130.1	6.46
O76369	Uncharacterized protein OS=Caenorhabditis elegans GN=CELE_F56H1.3 PE=4 SV=2 - [O76369_CAEEL]	1.85	1.02	1	1	1	1	1174	135.2	8.56
P50432	Serine hydroxymethyltransferase OS=Caenorhabditis elegans GN=mel-32 PE=1 SV=2 - [GLYC_CAEEL]	1.83	9.27	1	4	4	4	507	55.7	8.53
O45941	UPF0375 protein Y45F10C.2 OS=Caenorhabditis elegans GN=Y45F10C.2 PE=1 SV=1 - [U375E_CAEEL]	1.83	10.26	2	1	1	1	117	12.8	4.89
Q8MPW1	DEgenerin Like OS=Caenorhabditis elegans GN=del-6 PE=1 SV=2 - [Q8MPW1_CAEEL]	1.83	1.95	2	1	1	1	513	59.7	6.28
Q9XWJ6	Uncharacterized protein OS=Caenorhabditis elegans GN=CELE_Y51H1A.3 PE=1 SV=1 - [Q9XWJ6_CAEEL]	1.83	4.02	2	1	1	1	199	23.5	6.95
Q9N3F0	Ribosome production factor 2 homolog OS=Caenorhabditis elegans GN=Y54E10A.10 PE=3 SV=1 - [RPF2_CAEEL]	1.82	4.71	1	1	1	1	297	33.4	9.76
P30632	ATPase asna-1 OS=Caenorhabditis elegans GN=asna-1 PE=1 SV=1 - [ASNA_CAEEL]	1.82	3.80	1	1	1	1	342	37.5	4.93
Q86NE0	ASpartyl Protease OS=Caenorhabditis elegans GN=asp-2 PE=1 SV=1 - [Q86NE0_CAEEL]	1.82	2.80	2	1	1	1	429	46.6	5.20
G5EF37	Paralysed Arrest at Two-fold OS=Caenorhabditis elegans GN=pat-10 PE=1 SV=1 - [G5EF37_CAEEL]	1.82	4.35	1	1	1	1	161	18.5	4.31
P27798	Calreticulin OS=Caenorhabditis elegans GN=crt-1 PE=3 SV=1 - [CALR_CAEEL]	1.82	1.77	1	1	1	1	395	45.6	4.70

Q7KPV0	G protein, Subunit Alpha OS=Caenorhabditis elegans GN=gsa-1 PE=1 SV=1 - [Q7KPV0_CAEEL]	1.82	6.35	6	1	2	2	378	44.4	7.14
O44410	Ribosomal RNA-processing protein 8 OS=Caenorhabditis elegans GN=T07A9.8 PE=3 SV=1 - [RRP8_CAEEL]	1.81	6.71	1	1	1	1	343	39.4	9.45
Q19775	Protein phosphatase ppm-1 OS=Caenorhabditis elegans GN=ppm-1 PE=1 SV=1 - [PPM1_CAEEL]	1.81	3.63	1	1	1	1	468	51.9	5.17
D1MN68	G Protein, Beta subunit OS=Caenorhabditis elegans GN=gpb-1 PE=1 SV=1 - [D1MN68_CAEEL]	1.81	6.54	2	1	1	1	153	16.7	4.88
O17966	DNA topoisomerase 1 OS=Caenorhabditis elegans GN=top-1 PE=2 SV=1 - [TOP1_CAEEL]	1.81	0.99	1	1	1	1	806	94.0	8.98
P52717	Uncharacterized serine carboxypeptidase F41C3.5 OS=Caenorhabditis elegans GN=F41C3.5 PE=1 SV=1 - [YUW5_CAEEL]	1.80	2.13	1	1	1	1	469	53.6	6.70
I2HA98	PhenylAlanine Hydroxylase OS=Caenorhabditis elegans GN=pah-1 PE=1 SV=1 - [I2HA98_CAEEL]	1.78	2.21	2	1	1	1	453	51.8	6.07
Q21551	MICOS complex subunit MIC19 OS=Caenorhabditis elegans GN=chch-3 PE=3 SV=1 - [CHCH3_CAEEL]	1.77	7.69	1	1	1	1	169	19.2	5.54
G5EFH7	Arf-1 Guanine nucleotide Exchange Factor homolog OS=Caenorhabditis elegans GN=agef-1 PE=1 SV=1 - [G5EFH7_CAEEL]	1.77	2.38	2	2	2	2	1594	179.1	5.53
Q18674	Ribosomal RNA processing protein 1 homolog OS=Caenorhabditis elegans GN=C47E12.7 PE=3 SV=2 - [RRP1_CAEEL]	1.77	2.27	1	1	1	1	397	45.8	9.28
Q18508	Protein mel-28 OS=Caenorhabditis elegans GN=mel-28 PE=1 SV=2 - [MEL28_CAEEL]	1.77	0.50	1	1	1	1	1784	200.7	5.20
Q20143	unctional purine biosynthetic protein adenosine-3 OS=Caenorhabditis elegans GN=CELE_F38B6.4 PE=1 SV=3 - [Q20143_CAE	1.77	0.92	1	1	1	1	975	105.9	6.62
O61208	Gamma-tubulin complex component OS=Caenorhabditis elegans GN=gip-1 PE=1 SV=2 - [O61208_CAEEL]	1.76	1.46	2	1	1	1	891	100.2	8.59
Q95PZ4	Xeroderma Pigmentosum complementation group B) related OS=Caenorhabditis elegans GN=xpb-1 PE=1 SV=1 - [Q95PZ4_C	1.75	1.77	1	1	1	1	789	89.5	8.65
Q9N431	Uncharacterized protein OS=Caenorhabditis elegans GN=CELE_Y38C1AA.7 PE=1 SV=1 - [Q9N431_CAEEL]	1.74	10.06	1	1	1	1	159	16.8	5.80
Q93871	Equilibrative Nucleoside Transporter OS=Caenorhabditis elegans GN=ent-2 PE=1 SV=1 - [Q93871_CAEEL]	1.74	1.78	1	1	1	1	450	50.5	6.60
N1NVB5	K+/Cl-Cotransporter OS=Caenorhabditis elegans GN=kcc-1 PE=1 SV=1 - [N1NVB5_CAEEL]	1.73	2.22	5	2	2	2	1035	114.3	6.98
O62347	SLC (SoLute Carrier) homolog OS=Caenorhabditis elegans GN=slc-25a21 PE=1 SV=3 - [O62347_CAEEL]	1.72	3.81	1	1	1	1	289	31.8	9.33
Q10051	Pre-mRNA-processing factor 19 OS=Caenorhabditis elegans GN=prp-19 PE=3 SV=2 - [PRP19_CAEEL]	1.72	1.63	1	1	1	1	492	53.2	6.06
Q10941	Putative UDP-glucuronosyltransferase ugt-46 OS=Caenorhabditis elegans GN=ugt-46 PE=1 SV=1 - [UGT46_CAEEL]	1.72	3.39	3	2	2	2	531	60.2	7.28
G5EDJ3	Equilibrative Nucleoside Transporter OS=Caenorhabditis elegans GN=ent-1 PE=1 SV=1 - [G5EDJ3_CAEEL]	1.71	1.80	1	1	1	1	445	50.0	8.65
P34552	Apoptosis-linked gene 2-interacting protein X 1 OS=Caenorhabditis elegans GN=alx-1 PE=2 SV=3 - [ALXA_CAEEL]	1.70	1.36	1	1	1	1	882	98.2	6.58
Q9U2G5	Uncharacterized protein OS=Caenorhabditis elegans GN=mrp-7 PE=1 SV=3 - [Q9U2G5_CAEEL]	1.70	0.92	1	1	1	1	1525	170.9	6.44
Q9N5R9	FACT complex subunit spt-16 OS=Caenorhabditis elegans GN=spt-16 PE=3 SV=1 - [SPT16_CAEEL]	1.70	1.94	1	2	2	2	1030	116.8	5.77
G5EFP8	ACyLtransferase-like OS=Caenorhabditis elegans GN=acl-7 PE=1 SV=1 - [G5EFP8_CAEEL]	1.69	1.49	1	1	1	1	671	76.7	8.12
Q9XUJ7	Serine/threonine-protein kinase dkf-1 OS=Caenorhabditis elegans GN=dkf-1 PE=1 SV=1 - [DKF1_CAEEL]	1.69	1.66	1	1	1	1	722	80.9	7.23
P34342	Ran GTPase-activating protein 2 OS=Caenorhabditis elegans GN=ran-2 PE=4 SV=3 - [RGP2_CAEEL]	1.69	1.67	1	2	2	2	960	105.6	5.07
Q9XVS2	Uncharacterized protein OS=Caenorhabditis elegans GN=C25A1.4 PE=1 SV=1 - [Q9XVS2_CAEEL]	1.68	3.74	1	1	1	4	454	49.3	9.31
Q20086	Putative UDP-glucuronosyltransferase ugt-58 OS=Caenorhabditis elegans GN=ugt-58 PE=3 SV=2 - [UGT58_CAEEL]	1.68	4.13	1	2	2	2	533	61.1	8.02
Q9N599	Proteasome subunit alpha type-4 OS=Caenorhabditis elegans GN=pas-3 PE=3 SV=2 - [PSA4_CAEEL]	1.68	3.60	1	1	1	1	250	28.3	7.09
Q17561	Ref/ALY RNA export adaptor family OS=Caenorhabditis elegans GN=aly-1 PE=1 SV=1 - [Q17561_CAEEL]	1.67	4.04	1	1	1	1	223	24.3	11.33
Q9NAG2	Mitochondrial Ribosomal Protein, Large OS=Caenorhabditis elegans GN=mrpl-20 PE=1 SV=1 - [Q9NAG2_CAEEL]	1.67	5.24	1	1	1	1	191	22.6	9.23
O62178	Receptor Mediated Endocytosis OS=Caenorhabditis elegans GN=rme-8 PE=1 SV=3 - [O62178_CAEEL]	1.67	2.07	3	4	4	4	2271	258.2	6.76
Q19317	Putative neurobeachin homolog OS=Caenorhabditis elegans GN=sel-2 PE=2 SV=3 - [NBEA_CAEEL]	1.67	0.44	1	1	1	1	2507	281.0	5.91
G5EBK1	Na(+)/H(+) exchanger protein 7 OS=Caenorhabditis elegans GN=pbo-4 PE=1 SV=1 - [PBO4_CAEEL]	1.67	1.79	1	1	1	1	783	88.4	6.61
O01590	D (ACAD, acyl-CoA Dehydrogenase) Sequence homolog OS=Caenorhabditis elegans GN=acds-10 PE=1 SV=2 - [O01590_CA	1.67	1.52	1	1	1	1	985	111.6	8.63
Q9U3F8	Acid Alpha Glucosidase Relate OS=Caenorhabditis elegans GN=aagr-3 PE=1 SV=1 - [Q9U3F8_CAEEL]	1.67	1.21	2	1	1	1	910	103.7	5.95
Q17761	6-phosphogluconate dehydrogenase, decarboxylating OS=Caenorhabditis elegans GN=T25B9.9 PE=3 SV=2 - [6PGD_CAEEL]	1.67	2.27	1	1	1	1	484	53.2	6.90
Q9TXR4	MSH (MutS Homolog) family OS=Caenorhabditis elegans GN=msh-2 PE=1 SV=1 - [Q9TXR4_CAEEL]	1.66	1.18	1	1	1	1	849	95.8	5.83
Q9NA75	Trimeric intracellular cation channel type 1B.1 OS=Caenorhabditis elegans GN=Y57A10A.10 PE=1 SV=2 - [T38B1_CAEEL]	1.65	2.37	1	1	1	1	295	32.6	8.31
Q95QQ5	carboxamide ribonucleotide formylTransferase/IMP Cyclohydrolase homolog OS=Caenorhabditis elegans GN=atic-1 PE=1 SV=	1.65	3.37	3	1	1	1	386	42.3	6.62
Q9N4H4	Poly(ADP-ribose) polymerase pme-1 OS=Caenorhabditis elegans GN=pme-1 PE=2 SV=1 - [PME1_CAEEL]	1.64	1.27	1	1	1	1	945	107.9	7.97
Q8WTM6	Probable actin-related protein 2/3 complex subunit 2 OS=Caenorhabditis elegans GN=arx-4 PE=3 SV=1 - [ARPC2_CAEEL]	1.64	2.33	1	1	1	1	301	34.5	7.99
G5EG85	Spectrin beta chain OS=Caenorhabditis elegans GN=unc-70 PE=1 SV=1 - [G5EG85_CAEEL]	1.64	1.15	4	2	2	2	2257	262.1	5.47
P34529	Endoribonuclease dcr-1 OS=Caenorhabditis elegans GN=dcr-1 PE=1 SV=3 - [DCR1_CAEEL]	1.64	0.58	1	1	1	1	1910	218.3	5.69
O45628	Uncharacterized protein OS=Caenorhabditis elegans GN=CELE_H40L08.1 PE=1 SV=1 - [O45628_CAEEL]	1.63	2.03	1	1	1	1	444	51.1	5.66
G5EBW5	FErroChelatase-Like OS=Caenorhabditis elegans GN=fecl-1 PE=1 SV=1 - [G5EBW5_CAEEL]	1.63	6.42	1	2	2	2	374	42.7	8.85
Q965Q9	Uncharacterized protein OS=Caenorhabditis elegans GN=CELE_Y50D4C.3 PE=1 SV=1 - [Q965Q9_CAEEL]	1.63	2.98	1	1	1	1	605	66.4	9.76
P34722	Protein kinase C-like 1 OS=Caenorhabditis elegans GN=tpa-1 PE=1 SV=2 - [KPC1_CAEEL]	1.63	2.27	1	1	1	1	704	80.2	7.61
O02161	e mitochondrial import inner membrane translocase subunit tin-44 OS=Caenorhabditis elegans GN=tin-44 PE=3 SV=1 - [TIM44_	1.63	2.35	1	1	1	1	425	49.4	6.81
O62214	Uncharacterized protein OS=Caenorhabditis elegans GN=CELE_F33A8.4 PE=1 SV=1 - [O62214_CAEEL]	1.62	2.36	1	1	1	1	467	55.0	8.35
Q02331	Uncharacterized NTE family protein ZK370.4 OS=Caenorhabditis elegans GN=ZK370.4 PE=3 SV=3 - [YOL4_CAEEL]	1.62	0.89	1	1	1	1	1353	152.3	6.98
O17290	Uncharacterized protein OS=Caenorhabditis elegans GN=CELE_R52.5 PE=4 SV=1 - [O17290_CAEEL]	1.62	8.74	1	1	1	1	183	19.9	4.37
Q18164	Dihydropyrimidine dehydrogenase [NADP(+)] OS=Caenorhabditis elegans GN=dpyd-1 PE=3 SV=2 - [DPYD_CAEEL]	1.61	1.23	1	1	1	1	1059	115.2	6.46
G5EGB1	Galectin OS=Caenorhabditis elegans GN=lec-2 PE=1 SV=1 - [G5EGB1_CAEEL]	1.61	9.71	2	2	2	2	278	31.3	6.67
O76407	Uncharacterized protein OS=Caenorhabditis elegans GN=CELE_T10B5.3 PE=1 SV=3 - [O76407_CAEEL]	1.61	7.12	1	2	2	2	295	32.4	4.73
G5EDW8	Uncharacterized protein OS=Caenorhabditis elegans GN=CELE_VF13D12L.3 PE=1 SV=1 - [G5EDW8_CAEEL]	1.60	3.00	1	1	1	1	400	42.8	6.62

O18178	ne/threonine-protein phosphatase 2A regulatory subunit pptr-1 OS=Caenorhabditis elegans GN=pptr-1 PE=1 SV=4 - [2A51_CA	0.00	2.58	1	1	1	1	542	61.8	7.36
P53585	Probable ATP-citrate synthase OS=Caenorhabditis elegans GN=acly-1 PE=3 SV=1 - [ACLY_CAEEL]	0.00	1.54	1	1	1	1	1106	121.5	7.05
Q17334	Alcohol dehydrogenase 1 OS=Caenorhabditis elegans GN=sodh-1 PE=2 SV=2 - [ADH1_CAEEL]	0.00	2.01	1	1	1	1	349	37.7	6.52
P54216	Fructose-bisphosphate aldolase 1 OS=Caenorhabditis elegans GN=aldo-1 PE=1 SV=1 - [ALF1_CAEEL]	0.00	3.28	1	1	1	1	366	39.2	6.73
O01530	Aspartic protease 6 OS=Caenorhabditis elegans GN=asp-6 PE=3 SV=1 - [ASP6_CAEEL]	0.00	10.80	1	2	2	2	389	41.5	6.00
Q22230	Probable 2,4-dienoyl-CoA reductase 3 OS=Caenorhabditis elegans GN=decr-1.3 PE=3 SV=1 - [DECR_CAEEL]	0.00	4.21	2	1	1	1	309	33.1	8.40
Q17438	DnaJ homolog subfamily B member 1 OS=Caenorhabditis elegans GN=dnj-1 PE=1 SV=1 - [DNJ1_CAEEL]	0.00	3.49	1	1	1	1	401	46.6	9.04
Q19087	Aspartyl aminopeptidase OS=Caenorhabditis elegans GN=dnpp-1 PE=1 SV=1 - [DNPEP_CAEEL]	0.00	3.40	1	1	1	1	470	51.1	6.68
P49191	Putative fatty acid elongation protein 3 OS=Caenorhabditis elegans GN=elo-3 PE=1 SV=2 - [ELO3_CAEEL]	0.00	4.69	1	1	1	1	320	37.8	9.14
Q9XUP3	Eukaryotic translation initiation factor 3 subunit K OS=Caenorhabditis elegans GN=eif-3.K PE=2 SV=1 - [EIF3K_CAEEL]	0.00	8.75	1	1	1	1	240	27.0	6.92
Q09216	Putative endoplasmic reticulum metallopeptidase 1-A OS=Caenorhabditis elegans GN=B0495.7 PE=1 SV=2 - [ERP1A_CAEEL	0.00	1.45	1	1	1	1	895	102.1	7.02
Q19262	Exocyst complex component 3 OS=Caenorhabditis elegans GN=sec-6 PE=3 SV=2 - [EXOC3_CAEEL]	0.00	1.13	1	1	1	1	796	90.6	6.55
Q05036	Heat shock protein 110 OS=Caenorhabditis elegans GN=hsp-110 PE=3 SV=1 - [HS110_CAEEL]	0.00	2.19	1	1	1	1	776	86.8	5.44
Q18680	Probable inorganic pyrophosphatase 1 OS=Caenorhabditis elegans GN=pyp-1 PE=3 SV=4 - [IPYR_CAEEL]	0.00	8.41	1	2	2	2	428	46.4	6.16
O62415	Lysozyme-like protein 1 OS=Caenorhabditis elegans GN=lys-1 PE=2 SV=1 - [LYS1_CAEEL]	0.00	5.03	1	1	1	1	298	32.4	5.68
P34385	e methylcrotonoyl-CoA carboxylase beta chain, mitochondrial OS=Caenorhabditis elegans GN=F02A9.4 PE=3 SV=1 - [MCCB_	0.00	1.64	1	1	1	1	608	66.5	8.31
Q9XW10	Protein pad-1 OS=Caenorhabditis elegans GN=pad-1 PE=2 SV=2 - [PAD1_CAEEL]	0.00	0.33	1	1	1	1	2417	267.0	6.21
Q9TW67	ptide-N(4)-(N-acetyl-beta-glucosaminyl)asparagine amidase OS=Caenorhabditis elegans GN=png-1 PE=1 SV=1 - [NGLY1_CAE	0.00	3.14	1	2	2	2	606	69.1	7.03
P24892	NADH-ubiquinone oxidoreductase chain 4 OS=Caenorhabditis elegans GN=nduo-4 PE=3 SV=2 - [NU4M_CAEEL]	0.00	1.96	1	1	1	1	409	47.2	8.12
P91427	Probable phosphoglycerate kinase OS=Caenorhabditis elegans GN=pgk-1 PE=3 SV=1 - [PGK_CAEEL]	0.00	4.80	1	1	1	1	417	44.1	7.02
Q93841	utative 1-acyl-sn-glycerol-3-phosphate acyltransferase acl-1 OS=Caenorhabditis elegans GN=acl-1 PE=3 SV=2 - [PLC1_CAEE	0.00	4.58	1	1	1	1	262	29.6	9.01
Q9XW16	Profilin-1 OS=Caenorhabditis elegans GN=pfn-1 PE=2 SV=1 - [PROF1_CAEEL]	0.00	6.82	1	1	1	1	132	14.2	5.24
Q95XX1	Nicotinate phosphoribosyltransferase OS=Caenorhabditis elegans GN=nprt-1 PE=3 SV=3 - [PNCB_CAEEL]	0.00	1.60	1	1	1	1	562	63.3	6.64
P34305	Putative ATP-dependent RNA helicase rha-2 OS=Caenorhabditis elegans GN=rha-2 PE=3 SV=2 - [RHA2_CAEEL]	0.00	1.05	1	1	1	1	1148	129.9	5.82
Q9XVT0	Ribosome biogenesis regulatory protein homolog OS=Caenorhabditis elegans GN=rrbs-1 PE=3 SV=1 - [RRS1_CAEEL]	0.00	3.90	1	1	1	1	333	38.0	9.96
P91349	Spindle-defective protein 5 OS=Caenorhabditis elegans GN=spd-5 PE=1 SV=2 - [SPD5_CAEEL]	0.00	1.84	1	1	1	1	1198	135.1	5.29
O16259	Stress-induced-phosphoprotein 1 OS=Caenorhabditis elegans GN=sti-1 PE=1 SV=1 - [STIP1_CAEEL]	0.00	5.00	1	1	1	1	320	36.9	6.99
O01498	Signal transducing adapter molecule 1 OS=Caenorhabditis elegans GN=stam-1 PE=1 SV=2 - [STAM1_CAEEL]	0.00	4.16	1	1	1	1	457	50.8	5.29
O02495	Synaptobrevin-1 OS=Caenorhabditis elegans GN=snb-1 PE=1 SV=1 - [SYB1_CAEEL]	0.00	11.93	1	1	1	1	109	12.0	9.11
O17730	Putative thiosulfate sulfurtransferase mpst-1 OS=Caenorhabditis elegans GN=mpst-1 PE=3 SV=1 - [THT2_CAEEL]	0.00	3.05	1	1	1	1	328	36.9	6.89
Q09541	Tripeptidyl-peptidase 2 OS=Caenorhabditis elegans GN=tpp-2 PE=2 SV=1 - [TPP2_CAEEL]	0.00	0.80	1	1	1	1	1374	151.0	7.66
Q09278	Uncharacterized protein C45G9.5 OS=Caenorhabditis elegans GN=C45G9.5 PE=4 SV=1 - [YQI5_CAEEL]	0.00	5.38	1	1	1	1	316	36.0	5.34
P34304	Uncharacterized protein C06E1.9 OS=Caenorhabditis elegans GN=C06E1.9 PE=4 SV=2 - [YKQ9_CAEEL]	0.00	1.09	1	1	1	1	643	72.3	6.00
P34668	Putative ATP-dependent RNA helicase ZK686.2 OS=Caenorhabditis elegans GN=ZK686.2 PE=3 SV=2 - [YO12_CAEEL]	0.00	2.70	1	1	1	1	593	66.5	6.76
Q19905	UDP-glucose 6-dehydrogenase OS=Caenorhabditis elegans GN=sqv-4 PE=1 SV=1 - [UGDH_CAEEL]	0.00	4.78	1	2	2	2	481	52.7	6.32
Q17750	E3 UFM1-protein ligase 1 homolog OS=Caenorhabditis elegans GN=ufl-1 PE=3 SV=1 - [UFL1_CAEEL]	0.00	1.36	1	1	1	1	735	81.0	6.09
Q09299	Putative RNA-binding protein EEED8.10 OS=Caenorhabditis elegans GN=EEED8.10 PE=4 SV=3 - [YQOA_CAEEL]	0.00	2.17	1	1	1	1	738	81.1	7.52
A0A0M9JJ85	KETtiN (Drosophila actin-binding) homolog OS=Caenorhabditis elegans GN=ketn-1 PE=1 SV=1 - [A0A0M9JJ85_CAEEL]	0.00	0.46	12	1	1	1	3260	362.1	5.40
H2KZA5	Uncharacterized protein OS=Caenorhabditis elegans GN=CELE_R148.3 PE=1 SV=1 - [H2KZA5_CAEEL]	0.00	0.90	2	1	1	1	1115	122.9	5.07
O01882	ACyLtransferase-like OS=Caenorhabditis elegans GN=acl-14 PE=1 SV=3 - [O01882_CAEEL]	0.00	2.89	2	1	1	1	415	48.7	8.72
Q27GQ4	KiDINs220 (Vertebrate scaffold protein) homolog OS=Caenorhabditis elegans GN=kdin-1 PE=1 SV=3 - [Q27GQ4_CAEEL]	0.00	0.91	8	1	1	1	1433	159.6	5.91
G5EC72	ALP/Enigma encoding OS=Caenorhabditis elegans GN=alp-1 PE=1 SV=1 - [G5EC72_CAEEL]	0.00	3.06	5	1	1	1	425	45.9	8.62
Q86GU3	Uncharacterized protein OS=Caenorhabditis elegans GN=C32F10.8 PE=1 SV=1 - [Q86GU3_CAEEL]	0.00	4.86	2	1	1	1	350	38.7	7.71
Q9BL83	Related to yeast Vacuolar Protein Sorting factor OS=Caenorhabditis elegans GN=vps-4 PE=1 SV=4 - [Q9BL83_CAEEL]	0.00	2.56	1	1	1	1	430	48.1	6.76
O16376	Uncharacterized protein OS=Caenorhabditis elegans GN=CELE_M03F8.3 PE=1 SV=2 - [O16376_CAEEL]	0.00	1.34	2	1	1	1	744	87.9	5.26
Q58AU8	SAPK/ERK kinase OS=Caenorhabditis elegans GN=sek-4 PE=1 SV=2 - [Q58AU8_CAEEL]	0.00	11.81	1	1	1	2	364	40.4	7.66
Q86NC2	NADH Ubiquinone Oxidoreductase OS=Caenorhabditis elegans GN=nuo-2 PE=1 SV=1 - [Q86NC2_CAEEL]	0.00	4.10	1	1	1	1	268	30.8	8.47
V6CIR0	DYStrophin related OS=Caenorhabditis elegans GN=dys-1 PE=1 SV=1 - [V6CIR0_CAEEL]	0.00	15.44	9	1	1	1	149	16.2	5.36
Q19873	WD Repeat protein OS=Caenorhabditis elegans GN=wdr-46 PE=1 SV=1 - [Q19873_CAEEL]	0.00	2.41	1	1	1	1	580	65.8	9.31
Q22463	Suppressor of PAr-Two defect OS=Caenorhabditis elegans GN=spat-3 PE=1 SV=3 - [Q22463_CAEEL]	0.00	1.37	2	1	1	1	1092	120.4	9.20
P91175	SYF pre-mRNA splicing factor homolog OS=Caenorhabditis elegans GN=syf-1 PE=1 SV=1 - [P91175_CAEEL]	0.00	1.40	1	1	1	1	855	99.5	5.64
Q7Z121	C-type LECtin OS=Caenorhabditis elegans GN=clec-266 PE=1 SV=1 - [Q7Z121_CAEEL]	0.00	11.72	3	1	1	1	145	17.0	6.67
Q8I4K3	Uncharacterized protein OS=Caenorhabditis elegans GN=CELE_F30F8.9 PE=1 SV=1 - [Q8I4K3_CAEEL]	0.00	3.13	2	1	1	1	288	33.1	9.76
F3Y5P7	Glutamine(Q)-dependent NAD(+) Synthase OS=Caenorhabditis elegans GN=qns-1 PE=1 SV=1 - [F3Y5P7_CAEEL]	0.00	5.73	3	1	1	1	279	31.5	7.15
Q22090	PERMeable eggshell OS=Caenorhabditis elegans GN=perm-1 PE=1 SV=1 - [Q22090_CAEEL]	0.00	2.26	1	1	1	1	486	56.2	5.39
O44501	FarNesylTransferase, Alpha subunit OS=Caenorhabditis elegans GN=fnta-1 PE=1 SV=1 - [O44501_CAEEL]	0.00	3.35	1	1	1	1	328	38.4	5.50
Q95Y13	Nuclear Pore complex Protein OS=Caenorhabditis elegans GN=npp-8 PE=1 SV=1 - [Q95Y13_CAEEL]	0.00	8.03	1	2	2	2	411	44.9	5.64

P91457	Nuclear Pore complex Protein OS=Caenorhabditis elegans GN=npp-7 PE=1 SV=1 - [P91457_CAEEL]	0.00	1.48	1	1	1	1	1217	124.0	8.76
G5EEZ0	Uncharacterized protein OS=Caenorhabditis elegans GN=CELE_ZK1010.2 PE=1 SV=1 - [G5EEZ0_CAEEL]	0.00	3.21	1	1	1	1	374	42.9	8.00
V6CLN0	LIN-24 (Twenty-four) Like OS=Caenorhabditis elegans GN=lntl-1 PE=1 SV=1 - [V6CLN0_CAEEL]	0.00	8.53	3	1	1	1	129	14.6	5.12
Q7YZG8	AP complex subunit beta OS=Caenorhabditis elegans GN=apb-1 PE=1 SV=1 - [Q7YZG8_CAEEL]	0.00	1.93	2	1	1	1	827	90.3	5.21
Q17948	Protein Phosphatase 2A (Two A) Regulatory subunit OS=Caenorhabditis elegans GN=pptr-2 PE=1 SV=1 - [Q17948_CAEEL]	0.00	1.80	4	1	1	1	557	64.4	6.44
O17947	Uncharacterized protein OS=Caenorhabditis elegans GN=CELE_K10D11.4 PE=4 SV=3 - [O17947_CAEEL]	0.00	4.49	1	1	1	3	490	54.7	6.04
O76384	Yeast MMS related OS=Caenorhabditis elegans GN=mms-19 PE=1 SV=2 - [O76384_CAEEL]	0.00	1.08	1	1	1	1	922	105.3	5.27
Q22792	Uncharacterized protein OS=Caenorhabditis elegans GN=CELE_T25G3.3 PE=1 SV=2 - [Q22792_CAEEL]	0.00	1.56	1	1	1	1	513	58.6	5.71
Q20058	Uncharacterized protein OS=Caenorhabditis elegans GN=CELE_F35G12.12 PE=1 SV=3 - [Q20058_CAEEL]	0.00	2.44	1	1	1	1	491	55.8	5.35
Q2EEM4	PRotein arginine MethylTransferase OS=Caenorhabditis elegans GN=prmt-9 PE=1 SV=1 - [Q2EEM4_CAEEL]	0.00	3.46	2	1	1	1	347	40.0	5.59
G4S185	AANAT (Arylalkylamine N-AcetylTransferase) homolog OS=Caenorhabditis elegans GN=anat-1 PE=1 SV=2 - [G4S185_CAEEL	0.00	6.52	2	1	1	1	230	26.5	7.64
G5EG28	Eukaryotic Initiation Factor OS=Caenorhabditis elegans GN=eif-2bdelta PE=1 SV=1 - [G5EG28_CAEEL]	0.00	2.01	2	1	1	1	497	54.5	8.41
Q18037	Uncharacterized protein OS=Caenorhabditis elegans GN=C16A3.4 PE=1 SV=1 - [Q18037_CAEEL]	0.00	2.67	1	1	1	1	375	43.1	6.93
A0A131MCP7	UTX (Ubiquitously transcribed TPR on X) homolog OS=Caenorhabditis elegans GN=utx-1 PE=1 SV=1 - [A0A131MCP7_CAEEL	0.00	1.67	2	2	2	2	1141	131.2	6.37
O01917	Uncharacterized protein OS=Caenorhabditis elegans GN=CELE_F23H11.5 PE=1 SV=1 - [O01917_CAEEL]	0.00	8.33	1	1	1	1	96	11.0	10.17
G5ECC9	Uncharacterized protein OS=Caenorhabditis elegans GN=CELE_K11B4.1 PE=1 SV=1 - [G5ECC9_CAEEL]	0.00	3.77	1	1	1	1	424	48.2	5.38
Q20329	Uncharacterized protein OS=Caenorhabditis elegans GN=CELE_F42C5.9 PE=1 SV=1 - [Q20329_CAEEL]	0.00	2.72	1	1	1	1	552	61.9	5.88
M1Z882	Nuclear Pore complex Protein OS=Caenorhabditis elegans GN=npp-8 PE=1 SV=1 - [M1Z882_CAEEL]	0.00	4.79	1	2	2	2	376	43.0	8.02
A0A0K3AW63	Uncharacterized protein OS=Caenorhabditis elegans GN=emb-4 PE=1 SV=1 - [A0A0K3AW63_CAEEL]	0.00	2.39	2	1	1	1	419	49.1	7.39
A0A0K3ARM6	Uncharacterized protein OS=Caenorhabditis elegans GN=lin-23 PE=1 SV=1 - [A0A0K3ARM6_CAEEL]	0.00	2.30	4	1	1	1	610	69.7	7.65
Q9XXA2	Microtubule End Binding Protein OS=Caenorhabditis elegans GN=ebp-2 PE=1 SV=1 - [Q9XXA2_CAEEL]	0.00	6.35	1	1	1	1	299	33.0	5.07
O44991	LiPid Depleted OS=Caenorhabditis elegans GN=lpd-6 PE=1 SV=2 - [O44991_CAEEL]	0.00	1.92	1	1	1	1	573	63.7	10.11
O18687	Uncharacterized protein OS=Caenorhabditis elegans GN=CELE_F26E4.6 PE=1 SV=2 - [O18687_CAEEL]	0.00	34.52	1	1	1	1	84	9.3	11.05
Q9N3I1	Uncharacterized protein OS=Caenorhabditis elegans GN=CELE_Y51H7C.13 PE=1 SV=2 - [Q9N3I1_CAEEL]	0.00	4.36	1	1	1	1	344	36.1	5.55
Q22101	Uncharacterized protein OS=Caenorhabditis elegans GN=CELE_T02G5.7 PE=1 SV=1 - [Q22101_CAEEL]	0.00	4.87	1	1	1	1	390	40.7	5.38
Q8MXT6	COLlagen OS=Caenorhabditis elegans GN=col-106 PE=1 SV=1 - [Q8MXT6_CAEEL]	0.00	5.41	1	1	1	1	296	28.5	5.44
Q9TYV3	Uncharacterized protein OS=Caenorhabditis elegans GN=CELE_W07E6.2 PE=1 SV=1 - [Q9TYV3_CAEEL]	0.00	2.96	1	1	1	1	473	52.3	6.87
Q9N3D0	al peptidase complex catalytic subunit SEC11 OS=Caenorhabditis elegans GN=CELE_Y54E10BR.5 PE=1 SV=1 - [Q9N3D0_CA	0.00	4.37	1	1	1	1	183	20.6	8.12
Q95ZI4	Galectin OS=Caenorhabditis elegans GN=lec-3 PE=1 SV=1 - [Q95ZI4_CAEEL]	0.00	4.56	4	1	1	1	285	32.4	7.36
Q18032	Uncharacterized protein OS=Caenorhabditis elegans GN=C15H9.9 PE=1 SV=1 - [Q18032_CAEEL]	0.00	8.03	1	1	1	1	137	14.5	6.71
Q9GYI1	Uncharacterized protein OS=Caenorhabditis elegans GN=CELE_F29B9.11 PE=1 SV=1 - [Q9GYI1_CAEEL]	0.00	26.14	1	1	1	1	88	9.9	10.36
A0A131MB92	Fatty Acid CoA Synthetase family OS=Caenorhabditis elegans GN=acs-22 PE=1 SV=1 - [A0A131MB92_CAEEL]	0.00	2.48	3	1	1	1	525	58.4	7.23
O01755	daPtin, Mu/medium chain (Clathrin associated complex) OS=Caenorhabditis elegans GN=apm-1 PE=1 SV=2 - [O01755_CAEE	0.00	2.82	1	1	1	1	426	48.6	8.15
D2Y8W3	Uncharacterized protein OS=Caenorhabditis elegans GN=CELE_K10H10.13 PE=4 SV=1 - [D2Y8W3_CAEEL]	0.00	10.00	1	1	1	1	190	22.5	7.30
P91198	Protein Phosphatase Four Regulatory subunit OS=Caenorhabditis elegans GN=ppfr-2 PE=1 SV=1 - [P91198_CAEEL]	0.00	3.97	1	1	1	1	378	42.2	4.94
G5EFC4	Cell Division Cycle related OS=Caenorhabditis elegans GN=cdc-5l PE=1 SV=1 - [G5EFC4_CAEEL]	0.00	1.72	1	1	1	1	755	85.6	8.57
O17244	Transporter of SR proteins OS=Caenorhabditis elegans GN=tsr-1 PE=1 SV=3 - [O17244_CAEEL]	0.00	1.58	1	1	1	1	949	107.0	6.34
Q9XWG2	Uncharacterized protein OS=Caenorhabditis elegans GN=CELE_Y63D3A.7 PE=1 SV=2 - [Q9XWG2_CAEEL]	0.00	9.78	1	1	1	1	92	10.2	9.88
Q2AAB7	Uncharacterized protein OS=Caenorhabditis elegans GN=CELE_Y92H12A.5 PE=1 SV=1 - [Q2AAB7_CAEEL]	0.00	0.58	2	1	1	1	1375	157.3	6.11
O01483	INtegrator complex SubuniT 1 homolog OS=Caenorhabditis elegans GN=inst-1 PE=1 SV=2 - [O01483_CAEEL]	0.00	0.69	1	1	1	1	1884	213.6	6.71
Q9XW20	Uncharacterized protein OS=Caenorhabditis elegans GN=CELE_Y18D10A.11 PE=1 SV=1 - [Q9XW20_CAEEL]	0.00	4.03	1	1	1	1	248	27.9	4.89
G5EEQ8	Splicing Factor ThreeB (3b) Subunit homolog OS=Caenorhabditis elegans GN=sftb-1 PE=1 SV=1 - [G5EEQ8_CAEEL]	0.00	0.83	1	1	1	1	1322	147.2	6.34
O17869	CyCliN K OS=Caenorhabditis elegans GN=ccnk-1 PE=1 SV=2 - [O17869_CAEEL]	0.00	2.78	1	1	1	1	252	29.0	7.11
Q9XUU8	Uncharacterized protein OS=Caenorhabditis elegans GN=CELE_K05C4.5 PE=1 SV=3 - [Q9XUU8_CAEEL]	0.00	2.04	1	1	1	1	686	76.1	7.72
Q9XXI9	DNA helicase OS=Caenorhabditis elegans GN=mcm-2 PE=1 SV=1 - [Q9XXI9_CAEEL]	0.00	1.14	1	1	1	1	881	99.3	5.10
A0A0M7RE22	Uncharacterized protein OS=Caenorhabditis elegans GN=CELE_Y45G12B.3 PE=1 SV=1 - [A0A0M7RE22_CAEEL]	0.00	3.09	2	1	1	1	291	32.3	9.22
Q18183	Uncharacterized protein OS=Caenorhabditis elegans GN=C25H3.8 PE=1 SV=4 - [Q18183_CAEEL]	0.00	0.47	1	1	1	1	2350	266.4	7.53
Q9GYR5	Uncharacterized protein OS=Caenorhabditis elegans GN=C23G10.8 PE=1 SV=1 - [Q9GYR5_CAEEL]	0.00	1.71	1	1	1	1	937	106.6	6.24
Q17557	Yeast Suppressor of Cytochrome Oxidase deficiency) homolog OS=Caenorhabditis elegans GN=sco-1 PE=1 SV=1 - [Q17557_C	0.00	2.88	1	1	1	1	312	35.6	8.35
G5ECZ3	CELF35-1 OS=Caenorhabditis elegans GN=CELE_F35H10.10 PE=1 SV=1 - [G5ECZ3_CAEEL]	0.00	0.43	1	1	1	1	1404	157.8	6.54
G5EDP6	Flavin-containing monooxygenase OS=Caenorhabditis elegans GN=fmo-1 PE=1 SV=1 - [G5EDP6_CAEEL]	0.00	1.31	1	1	1	1	533	61.2	7.37
G5EC53	Lysophospholipid acyltransferase OS=Caenorhabditis elegans GN=mboa-3 PE=1 SV=1 - [G5EC53_CAEEL]	0.00	1.25	1	1	1	1	480	55.2	9.09

Table S2. Statistics of data collection and model refinement.

**Data collection**	
Space group	*C* 2
Cell dimensions	
a , *b* , *c* (Å)	40.274 163.515 229.132
α, β, γ (°)	90.00 90.46 90.00
Resolution (Å)	50–2.4 (2.44-2.4)
R_{merge} [a]	0.129 (0.762)
$I/\sigma I$	10.9 (1.5)
$CC_{1/2}$ [b]	1.0 (0.775)
Completeness (%)	95.3 (97.0)
Redundancy	5.2 (4.9)
**Refinement**	
Resolution (Å)	50-2.4 (2.46-2.4)
No. reflections	54870 (3921)
R_{work} / R_{free} [c]	0.253 (0.343) / 0.285 (0.337)
No. atoms	
Protein	9280
Ligand/ion	18
Water	26
Mean *B* (Å)	
Protein	66.8
Ligand/ion	68.4
Water	42.5
r.m.s. deviations	
Bond lengths (Å)	0.003
Bond angles (°)	0.7
Ramachandran analysis	
Favored region (%)	97.9
Allowed region (%)	2.1
Outliers (%)	0

The numbers in parentheses represent values for the highest resolution shell.

[a] $R_{merge} = \sum|I_i - I_m|/\sum I_i$, where Ii is the intensity of the measured reflection and I_m is the mean intensity of all symmetry related reflections.

[b] $CC_{1/2}$ is the correlation coefficient of the half datasets.

[c] $R_{work} = \Sigma||F_{obs}| - |F_{calc}||/\Sigma|F_{obs}|$, where F_{obs} and F_{calc} are observed and calculated structure factors.

$R_{free} = \Sigma_T||F_{obs}| - |F_{calc}||/\Sigma_T|F_{obs}|$, where T is a test data set of about 4 % of the total reflections randomly chosen and set aside prior to refinement.

Table S3. C. elegans strains used in this study.

# Strains	Marker	Genotype
**N2**	-	Ancestral N2 Bristol strain; "wild-type"
**YC0156**	GFP::SAO-1	*yc09*[GFP::3XFlag::*sao-1*]
**YC0272**	SAO-1:: RFP	*yc011*[*sao-1*::TagRFP::3XFlag]
**YC0711**	PGL-1:: RFP; GFP::SAO-1	*pgl-1*::3XFlag::TagRFP; *yc09*[GFP::3XFlag::*sao-1*]
**MD701**	CED-1::GFP	*bcls39*[*lim-7p*::*ced-1*::GFP + *lin-15*(+)]
**YC0571**	CED-1::GFP	*yc012*[*sao-1 △C*::3XFlag]; *bcls39*[*lim-7p*::*ced-1*::GFP + *lin-15*(+)]
**YC0576**	CED-1::GFP	*yc013*[*sao-1p*::BFP::3XFlag]; *bcls39*[*lim-7p*::*ced-1*::GFP + *lin-15*(+)]
**YC0627**	SAO-1 (187-189-2A)::RFP; CED-1::GFP	*yc014*[*sao-1* (187-189-2A)::TagRFP::3XFlag]; *bcls39*[*lim-7p*::*ced-1*::GFP + *lin-15*(+)]
**YC0634**	SAO-1 (199-201-3A)::RFP; CED-1::GFP	*yc015*[*sao-1* (199-201-3A)::TagRFP]::3XFlag; *bcls39*[*lim-7p*::*ced-1*::GFP + *lin-15*(+)]
**YC0655**	SAO-1 (187-201-5A)::RFP; CED-1::GFP	*yc016*[*sao-1* (187-201-5A)::TagRFP::3XFlag]; lim-7p::ced-1::GFP + lin-15(+)
**YC0698**	SAO-1::RFP; CED-1::GFP	*yc017*[Ex[*pie-1p*::*sao-1*::TagRFP::3Xflag::*sao-1* 3'*utr*]]; yc012[*sao-1 △C*]; *bcls39*[*lim-7p*::*ced-1*::GFP + *lin-15*(+)]
**YC0710**	SAO-1::RFP; SAO-1P::BFP; CED-1::GFP	*yc017*[Ex[*pie-1p*::*sao-1*::TagRFP::3Xflag::*sao-1* 3'*utr*]];yc013 [*sao-1p*::BFP]; *bcls39*[*lim-7p*::*ced-1*::GFP + *lin-15*(+)]
**OLS400**	DLC-1::GFP	*aarSi1*[*dlc-1p*::*dlc-1*::GFP *cb-unc-119*(+)]
**YC0690**	SAO-1P::RFP; DLC-1::GFP	*yc018*[*sao-1p*::TagRFP::3XFlag]; *aarSi1*[*dlc-1p*::*dlc-1*::GFP *cb-unc-119*(+)]
**YC0568**	SAO-1::RFP; DLC-1::GFP	*yc011*[*sao-1*::TagRFP::3XFlag]; *aarSi1*[*dlc-1p*::*dlc-1*::GFP *cb-unc-119*(+)]
**YC0474**	DLC-1::GFP	*yc012*[*sao-1 △C*::3XFlag]; *aarSi1*[*dlc-1p*::*dlc-1*::GFP *cb-unc-119*(+)]
**YC0653**	SAO-1::RFP; DLC-1::GFP	*yc017*[Ex[*pie-1p*::*sao-1*::TagRFP::3XFlag::*sao-1* 3'*utr*]]; *yc012*[*sao-1 △C*]; *aarSi1*[*dlc-1p*::*dlc-1*::GFP *cb-unc-119*(+)]
**YC0631**	SAO-1::RFP; SAO-1P::BFP; DLC-1::GFP	*yc017*[Ex[*pie-1p*::*sao-1*::TagRFP::3Xflag::*sao-1* 3'*utr*]]; *yc013*[*sao-1p*::BFP::3XFlag]; *aarSi1*[*dlc-1p*::*dlc-1*::GFP *cb-unc-119*(+)]
**YC0633**	SAO-1 (187-189-2A)::RFP; DLC-1::GFP	*yc014*[*sao-1*(187-189-2A)::TagRFP::3XFlag]; *aarSi1*[*dlc-1p*::*dlc-1*::GFP *cb-unc-119*(+)]
**YC0626**	SAO-1 (199-201-3A)::RFP; DLC-1::GFP	*yc015*[*sao-1* (199-201-3A)::TagRFP::3XFlag]; *aarSi1*[*dlc-1p*::*dlc-1*::GFP *cb-unc-119*(+)]
**YC0652**	SAO-1 (187-201-5A)::RFP; DLC-1::GFP	*yc016*[*sao-1* (187-201-5A)::TagRFP::3XFlag]; *aarSi1*[*dlc-1p*::*dlc-1*::GFP *cb-unc-119*(+)]
**opIs219**	CED-4::GFP	*opIs219*[*ced-4p*::*ced-4*::GFP *unc-119(+)]*
**YC0569**	SAO-1P::BFP; CED-4::GFP	*yc013*[*sao-1p*::BFP::3XFlag]; *opIs219*[*ced-4p*::*ced-4*::GFP *unc-119(+)]*
**YC0687**	SAO-1::RFP; SAO-1P::BFP; CED-4::GFP	*yc014*[*sao-1*(187-189-2A)::TagRFP::3XFlag]; *opIs219*[*ced-4p*::*ced-4*::GFP *unc-119(+)]*
**YC0688**	SAO-1 (187-189-2A)::RFP; CED-4::GFP	*yc015*[*sao-1* (199-201-3A)::TagRFP::3XFlag]; *opIs219*[*ced-4p*::*ced-4*::GFP *unc-119(+)]*
**YC0689**	SAO-1 (199-201-3A)::RFP; CED-4::GFP	*yc016*[*sao-1* (187-201-5A)::TagRFP::3XFlag]; *opIs219*[*ced-4p*::*ced-4*::GFP *unc-119(+)]*
**YC0700**	RFP::CED-9	*yc019*[TagRFP::*ced-9*::3XFlag]
**KX110**	CED-1::GFP	*ced-9(n1653) mab-5(mu14)*III; *bcls39* V

Table S4. Primers used in this study.

Primers for pDD162 construction	Sequence (5'-3')
sao-1-N-F	GTTCTTGTGCATCGCGCCGTGTTTTAGAGCTAGAAATAGCAAGT
sao-1-N-R	CAAGACATCTCGCAATAGG
sao-1-C-F	CGTGTTCTTTCCGAGTTGCTGTTTTAGAGCTAGAAATAGCAAGT
sao-1-C-R	CAAGACATCTCGCAATAGG
ced-9-F	GCGTATCGGCGACGAACGAGTTTTAGAGCTAGAAATAG
ced-9-R	CAAGACATCTCGCAATAGG
**Primers for pDD282 construction**	**Sequence (5'-3')**
gfp::sao-1-P1	ACGTTGTAAAACGACGGCCAGTCGCCGGCAAATGAAACGCAACA AGTC
gfp::sao-1-P2	TCCAGTGAACAATTCTTCTCCTTTACTCATGGCTCCATCCGTATTT TGATGTCACTAG
gfp::sao-1-P3	CGTGATTACAAGGATGACGATGACAAGAGAATGCACAAGAACGG AAAC
gfp::sao-1-P4	TCACACAGGAAACAGCTATGACCATGTTATGCTAGGGACAAGTC ATCAC
sao-1p::bfp-P1	CGTTGTAAAACGACGGCCAGTCGCCGGCA AATGAAACGCAACAAGTC
sao-1p::bfp-P2	GTGACATCAAAATACCGGATGGAGCCATGTCCGAACTCATCAAG G
sao-1p::bfp-P3	GATGACGATGACAAGAGAATGCACAAGAACGGAAAC
sao-1p::bfp-P4	GCTATGACCATGTTATGTTGATGATAACCATTTG
sao-1△C-P1	ACGTTGTAAAACGACGGCCAGTCGCCGGCAAATGAAACGCAACA AGTC
sao-1△C-P2	CAGCACATCCCACAGGAGAATCTGTACTTTCAATC
sao-1△C-P3	CTGTGGGATGTGCTGATG
sao-1△C-P4	TCACACAGGAAACAGCTATGACCATGTTATGCTAGGGACAAGTC ATCAC
**Primers for pDD284 construction**	**Sequence (5'-3')**
sao-1::rfp-P1	CGTTGTAAAACGACGGCCAGTCGCCGGCA TTTCCCACCATTCCTTCC
sao-1::rfp-P2	CATCGATGCTCCTGAGGCTCCCGATGCTCCGTTGATGATAACCAT TTGTCCCAAGAGTTCCGAAAGAACACGAGAAGCC
sao-1::rfp-P3	CGTGATTACAAGGATGACGATGACAAGAGATAAATCTTCTCTTCA TTTTC
sao-1::rfp-P4	GGAAACAGCTATGACCATGTTATCGATTTC TCCTGGATCGGATGAAAT
sao-1(187-189-2A)::rfp-P1	CGTTGTAAAACGACGGCCAGTCGCCGGCA TTTCCCACCATTCCTTCC
sao-1(187-189-2A)::rfp-P2	ATGCT GCC GCCGACCAAGTTGTGATGA
sao-1(187-189-2A)::rfp-P3	GTCGGCGGCAGCATTAGCGTGCTGCATCT
sao-1(187-189-2A)-rfp-P4	GGAAACAGCTATGACCATGTTATCGATTTC TCCTGGATCGGATGAAAT
sao-1(199-201-3A)::rfp-P1	CGTTGTAAAACGACGGCCAGTCGCCGGCA TTTCCCACCATTCCTTCC
sao-1(199-201-3A)::rfp-P2	GTGATGAAGAGCGTCGAGGCTGCAGCA
sao-1(199-201-3A)::rfp-P3	CTAACCTCAACTGGCTCTGCTGCAGC
sao-1(199-201-3A)::rfp-P4	GGAAACAGCTATGACCATGTTATCGATTTC TCCTGGATCGGATGAAAT
sao-1(187-201-5A)::rfp-P1	CGTTGTAAAACGACGGCCAGTCGCCGGCA TTTCCCACCATTCCTTCC
sao-1(187-201-5A)::rfp-P2	GTGATGAAGAGCGTCGAGGCTGCAGCA
sao-1(187-201-5A)::rfp-P3	CTAACCTCAACTGGCTCTGCTGCAGC

sao-1(187-201-5A)::rfp-P4	GGAAACAGCTATGACCATGTTATCGATTTC TCCTGGATCGGATGAAAT
rfp::ced-9-P1	GGCCAGTCGCCGGCACACAGTCCGAGGCAAAGACG
rfp::ced-9-P2	TCCCTTGGAGACCATCTAAAATTTTTATTCGTT
rfp::ced-9-P3	GACGATGACAAGAGAATGACACGCTGCACGGCG
rfp::ced-9-P4	CTATGACCATGTTATTTTCTGCCACCTTTCTGG
**Primers for PCFJ90 construction**	**Sequence (5'-3')**
PCFJ90-F	CAACTTTATTATACATAGTTGA
PCFJ90-R	CAACTTTTCTATACAAAG
pie-1p-F	TGTATAGAAAAGTTGCACATACAGGTGACGCAGAGG
pie-1p-R	ATGGTCTCCAAGGGAGAG
sao-1-F	ATGCACAAGAACGGAAACAATG
sao-1-R	CATCGATGCTCCTGAGGCTCCCGATGCTCCGTTGATGATAACCAT TTGTCCCAAGAGTTCCGAAAGAACACGAGAAGCC
rfp-F	ATGGTCTCCAAGGGAGAG
sao-1 3’utr-R	TGTATAATAAAGTTGCATGTCACAAATGAACTC
