# Supplementary material for: Interaction between DLC-1 and SAO-1 facilitates CED-4 translocation during apoptosis in the Caenorhabditis elegans germline
Source: Cell Death Discov. 2022 Nov 3;8:441. doi: 10.1038/s41420-022-01233-9 (PMC9630320; doi:10.1038/s41420-022-01233-9)
Supplement: Supplementary file 2 — Supplemental Figures [file 41420_2022_1233_MOESM2_ESM.pdf]

Supplemental Figures

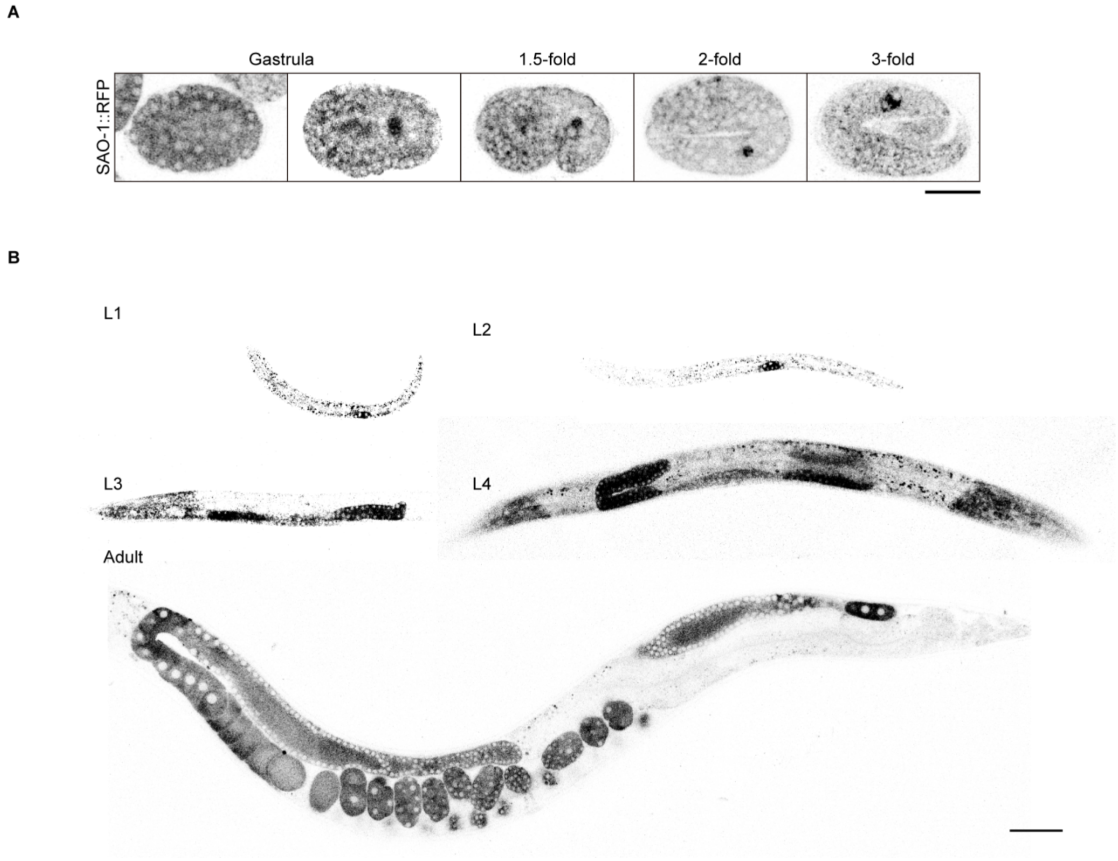

Figure S1. The expression of SAO-1::RFP in *C. elegans* primordial germ cells and germline.

(A) Representative confocal images of the expression of SAO-1::RFP in the embryos at the gastrula stage, 1.5-fold stage, 2-fold stage and 3-fold stage. Scale bar, 20 μm.

(B) Representative confocal images of the expression of SAO-1::RFP in worms at L1, L2, L3, L4 and Adult stage. Scale bar, 50 μm.

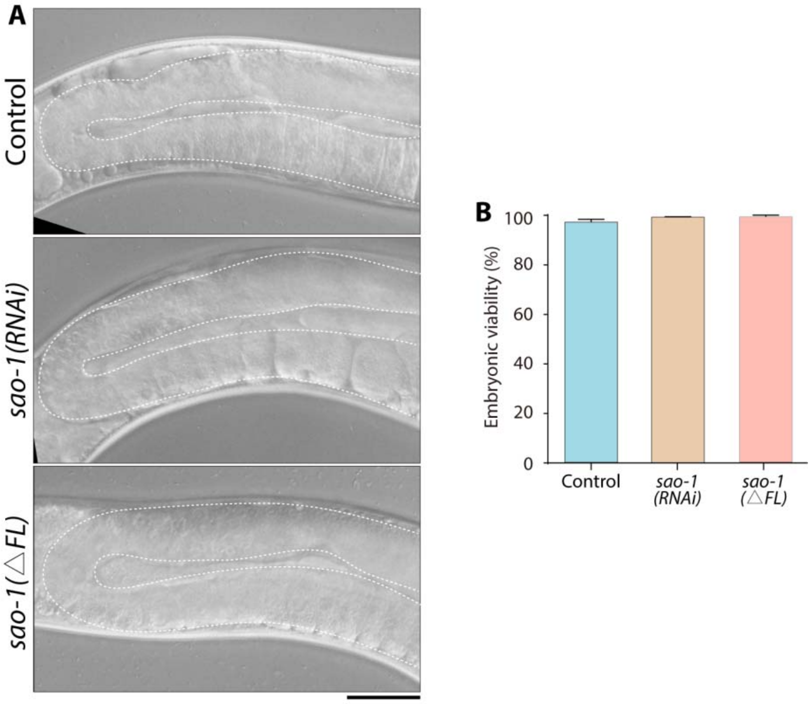

Figure S2. SAO-1 depletion does not affect germline morphology and embryonic viability.

(A) Representative DIC images of germline in control, *sao-1(RNAi),* and *sao-1(△FL)*. Scale bar, 50 μm.

(B) Quantification of embryonic viability in control (96.62%, n=9), *sao-1(RNAi)* (98.93%, n=9), and *sao-1(△FL)* (99.36%, n=9).

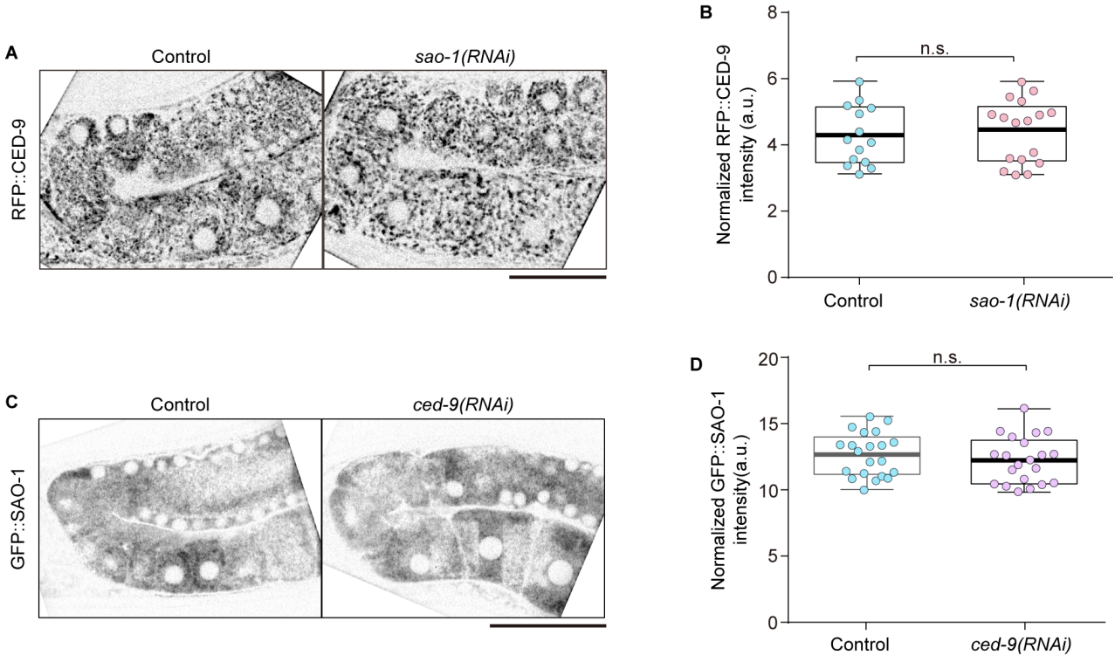

Figure S3. SAO-1 do not regulate the expression and subcellular localization of CED-9.

(A) Representative confocal images of RFP::CED-9 in control and *sao-1(RNAi).*

(B) Quantification of normalized RFP::CED-9 intensity in (A).

(C) Representative confocal images of GFP::SAO-1 in control and *ced-9(RNAi)*.

(D) Quantification of normalized GFP::SAO-1 intensity in (C).

Each dot in the chart represents a single worm. Data were analyzed by two-tailed Student's t-test; n.s., not significant; a.u., arbitrary unit; Scale bar, 50 μm.

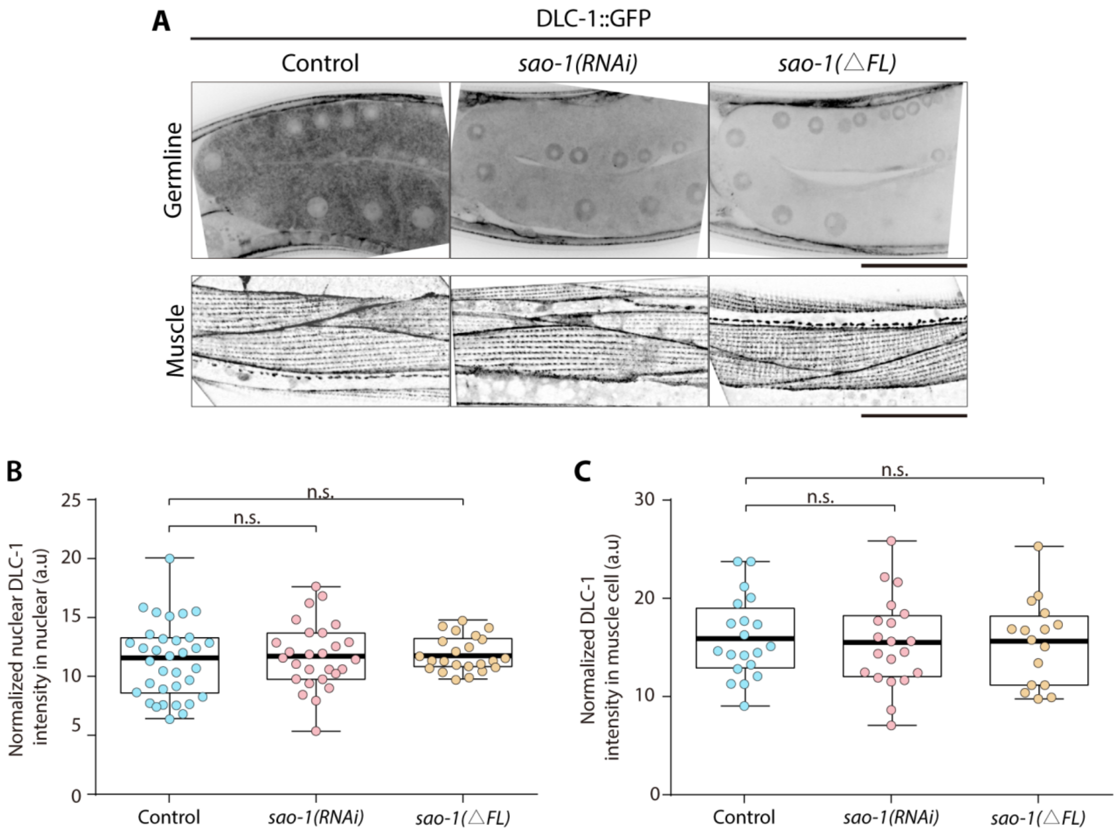

Figure S4. SAO-1 does not regulate DLC-1 in muscle cells and in the nucleus of germ cells.

(A) Representative confocal images of DLC-1::GFP in the nucleus of germ cells and muscle cells in control , *sao-1(RNAi),* and *sao-1(△FL)* mutant.

(B) Quantification of normalized DLC-1::GFP intensity in the nucleus of (A).

(C) Quantification of normalized DLC-1::GFP intensity in the muscle cells of (A).

Each dot in the chart represents a single worm. Data were analyzed by two-tailed Student's t-test; n.s., not significant; a.u., arbitrary unit; Scale bar, 50 μm.

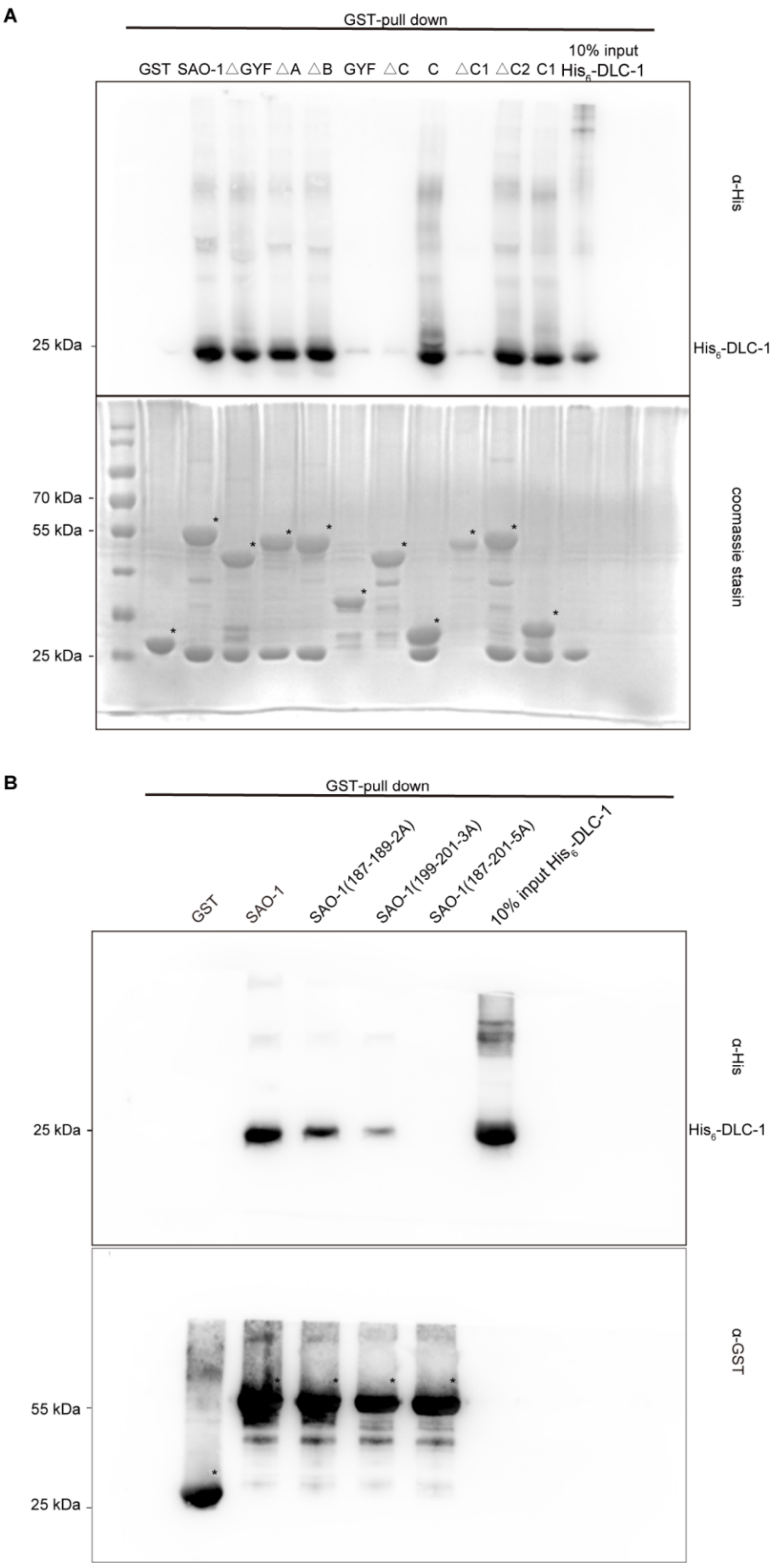

Figure S5. Full length uncropped original western blots figure.

(A) Full blots of Figure 4D. Western blot analysis of DLC-1 binding to purified GST-

tag fusion proteins (GST, GST-SAO-1, GST-△GYF(△12-73), GST-△A(△102-122), GST-△B(△167-181), GST-GYF(1-73), GST-△C(△182-226), GST-C(182-226), GST-△C1(△182-205), GST-△C2(△206-226), GST-C1 (182-205)) using His antibody (top). GST-tag fusion proteins were visualized by Coomassie blue staining (bottom). Asterisks denote GST-tag fusion proteins.

(B) Full blots of Figure 5B. Western blot analysis of DLC-1 binding to purified GST, GST-SAO-1, GST-SAO-1(187-189-2A), GST-SAO-1(199-201-3A), GST-SAO-1(187-201-5A) using His antibody (top). GST-tag fusion proteins were visualized using GST antibody (bottom). Asterisks denote GST-tag fusion proteins.
