# Supplementary figures and images for: Interaction between DLC-1 and SAO-1 facilitates CED-4 translocation during apoptosis in the Caenorhabditis elegans germline
Source: Cell Death Discov. 2022 Nov 3;8:441. doi: 10.1038/s41420-022-01233-9 (PMC9630320; doi:10.1038/s41420-022-01233-9)

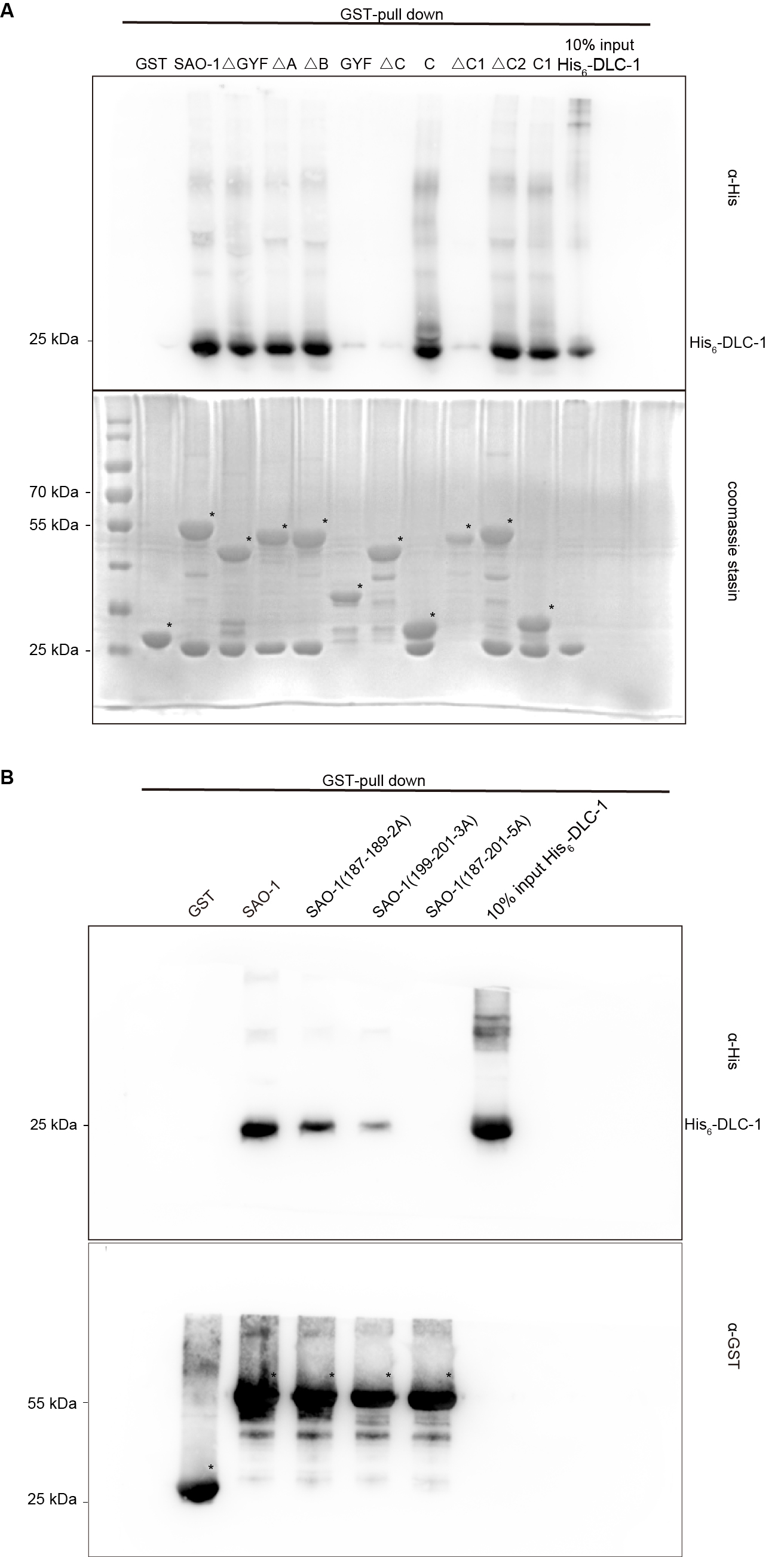

Supplement: Supplementary file 3 — Original Data File [file 41420_2022_1233_MOESM3_ESM.jpg]
